# Supplementary material for: MCE domain proteins: conserved inner membrane lipid-binding proteins required for outer membrane homeostasis
Source: Sci Rep. 2017 Aug 17;7:8608. doi: 10.1038/s41598-017-09111-6 (PMC5561183; doi:10.1038/s41598-017-09111-6)
Supplement: Supplementary file 1 — Supplementary Information [file 41598_2017_9111_MOESM1_ESM.pdf]

MCE domain proteins: conserved inner membrane lipid-binding proteins required for outer membrane homeostasis

Georgia L. Isom[1], Nathaniel J. Davies[2], Zhi-Soon Chong[3], Jack A. Bryant[1], Mohammed Jamshad[1], Maria Sharif[1], Adam F. Cunningham[1], Timothy J. Knowles[1], Shu-Sin Chng[3], Jeffrey A. Cole[1], Ian R. Henderson[1*]

1. Institute of Microbiology and Infection, University of Birmingham
2. Institute of Immunology and Immunotherapy, University of Birmingham
3. Department of Chemistry, National University of Singapore
* Corresponding author: I.R.Henderson@bham.ac.uk

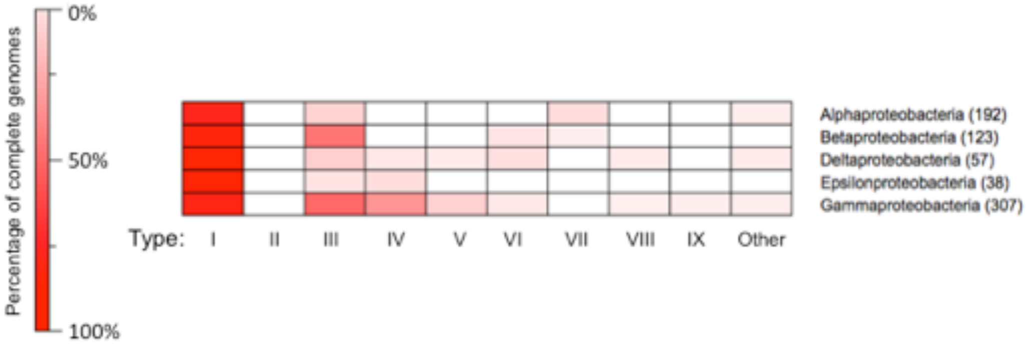

Supplementary figure S1. A heat map showing the distribution of MCE protein architectures across the major classes of Proteobacteria. For architecture information see supplementary table 1. The colours are based on percentages ranging from 0% (white) to 100% (bright red). The classes are displayed on the right of the heat map and the number of species is in brackets.

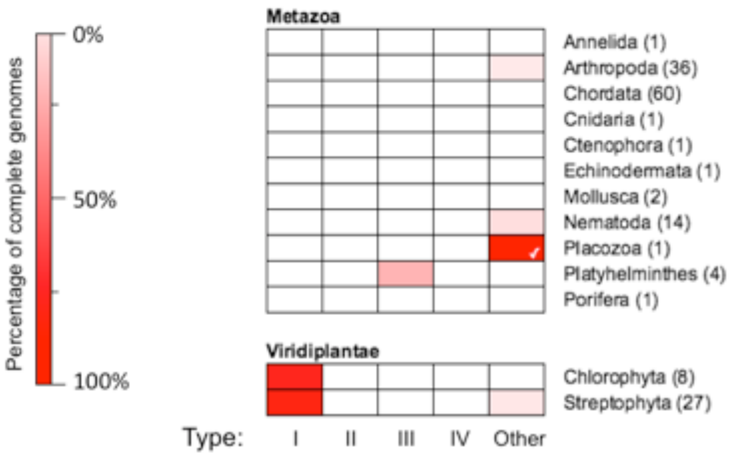

Supplementary figure S2. A heat map showing the distribution of MCE proteins across Metazoa and Viridiplantae. The colours are based on percentages ranging from 0% (white) to 100% (bright red). The classes are displayed on the right of the heat map and the number of species is in brackets.

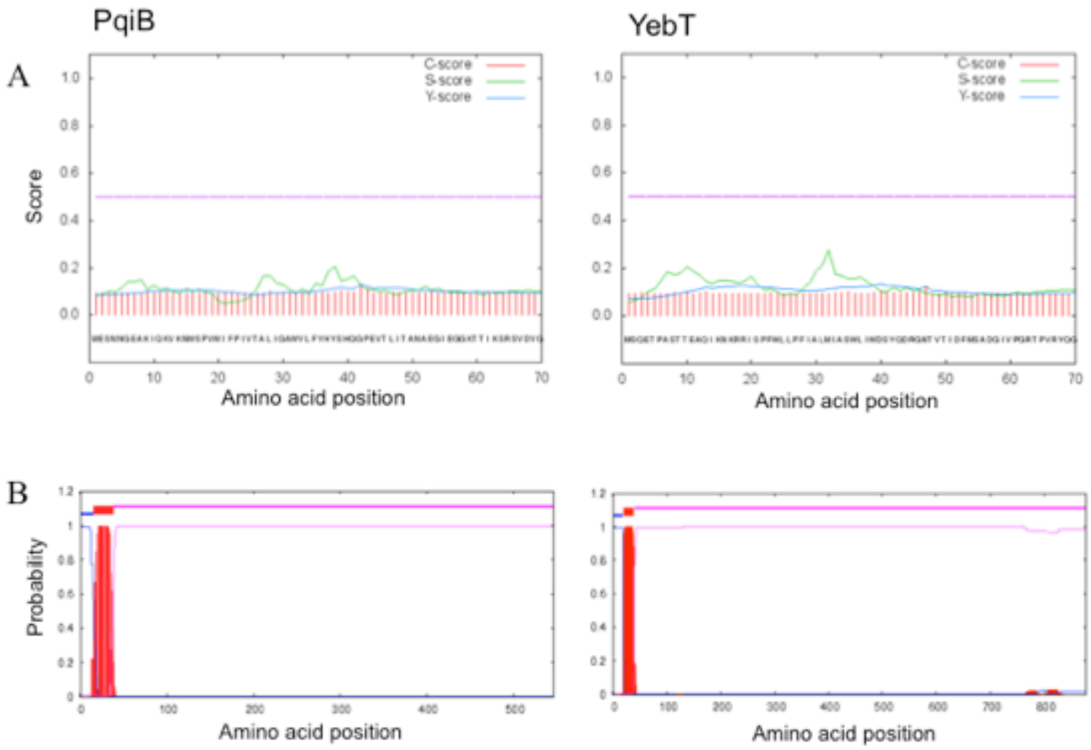

Supplementary figure S3. A) Cleavable signal peptide predictions of PqiB and YebT using SignalP 4.1 server and B) the transmembrane helix predictions of PqiB and YebT using the TMHMM server.

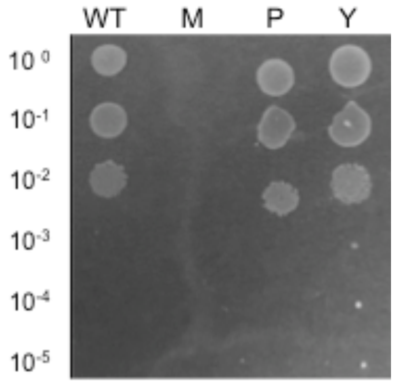

Supplementary figure S4. Dilutions of the WT strain and *ΔmlaD*, *ΔpqiAB* and *ΔyebST* deletion strains on LB agar supplemented with 0.5% SDS and 1.1 mM EDTA. The plates were prepared as described previously.

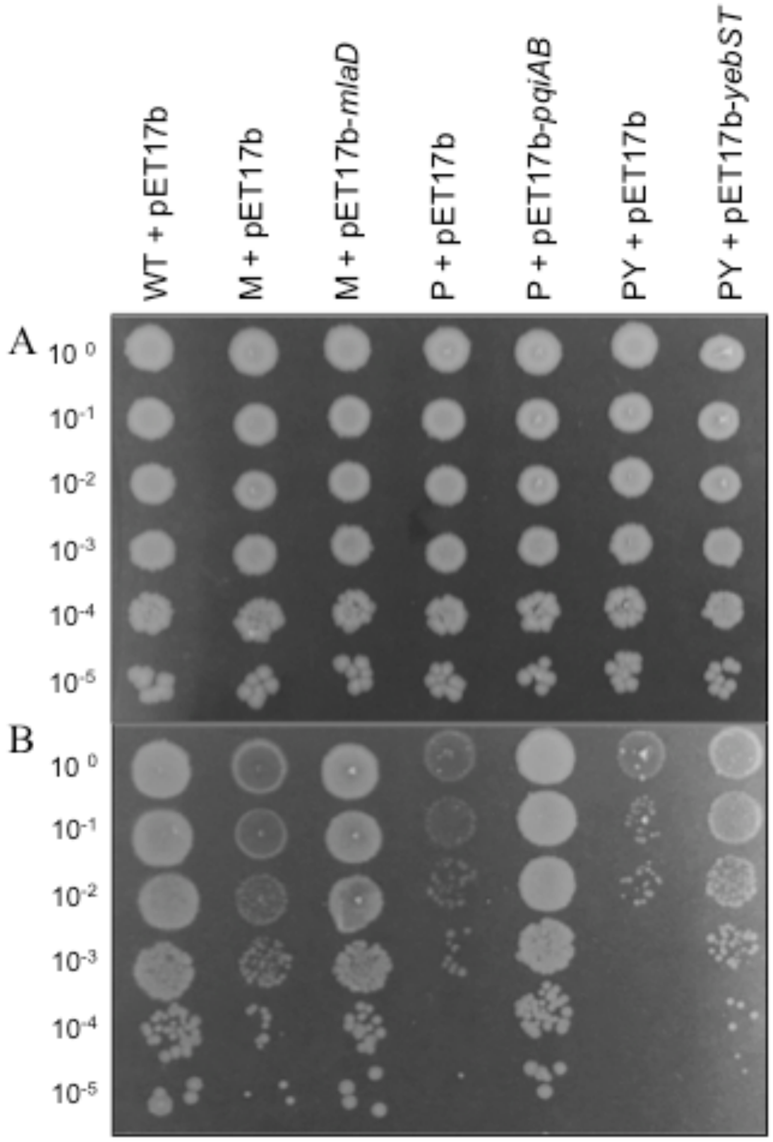

Supplementary figure S5. Complementation of the phenotypes of *mlaD*, *pqiAB* and *pqiAB yebST* mutants on 1% lauryl sulfobetaine: (A) LB agar, (B) 1% lauryl sulfobetaine. The plates were prepared as described previously.

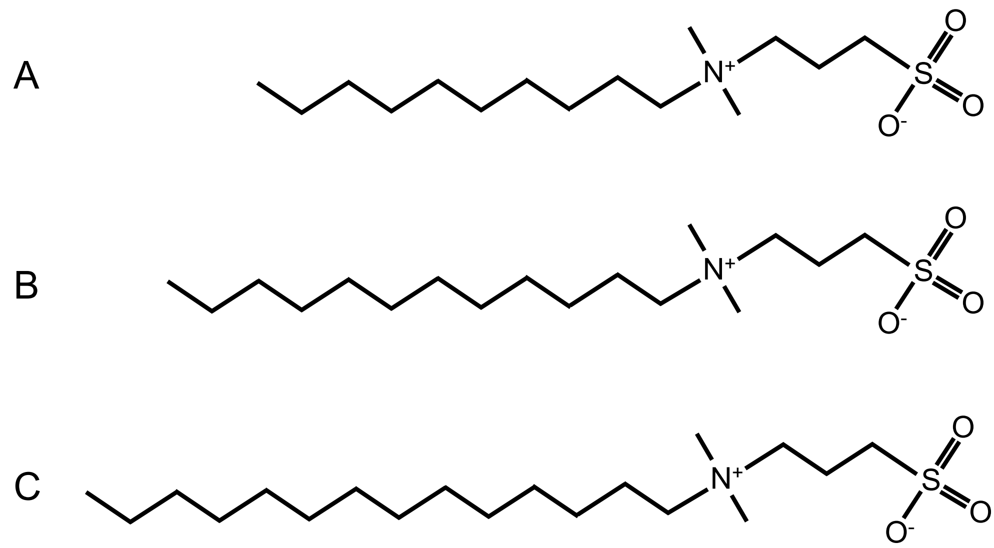

Supplementary figure S6. Chemical structures of A) caprylyl sulfobetaine B) lauryl sulfobetaine and C) myristyl sulfobetaine.

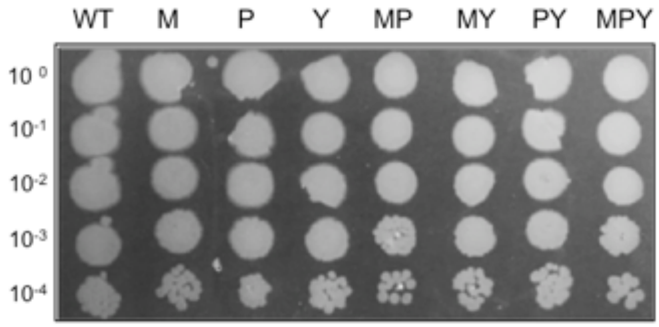

Supplementary figure S7. Dilutions of the WT strain and all mutants on LB agar supplemented with 1% SDS. The plates were prepared as described previously.

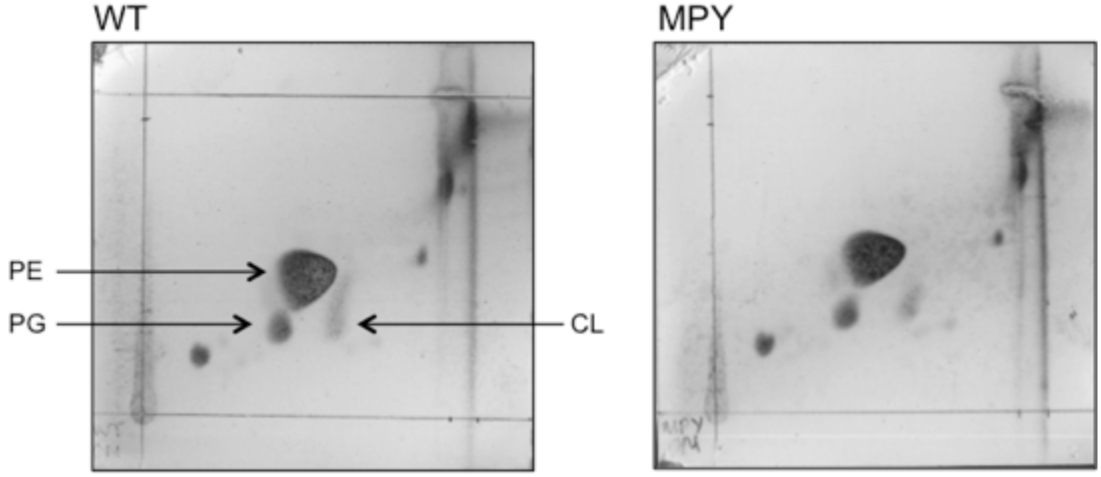

Supplementary figure S8. Outer membrane lipid profiles of the parent strain and the *mlaD pqiAB yebST* mutant. Lipids were extracted from outer membranes and separated by TLC in 65:25:4 chloroform:methanol:water (direction 1) and 80:12:15:4 chloroform:methanol: acetic acid:water (direction 2).

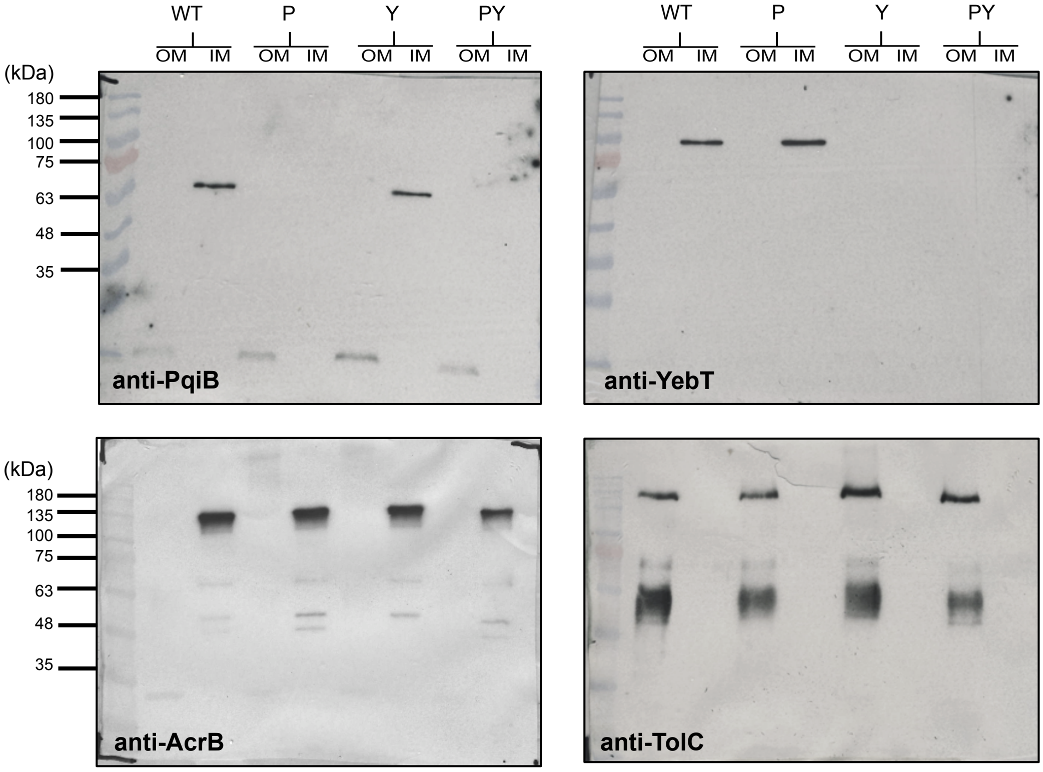

Supplementary figure S9. Full western blots to identify the locations of PqiB and YebT in *E. coli* K-12 BW25113.

Supplementary table S1: Architectures for MCE proteins

Architecture no.	No. proteins	Description
1	11012	MCE
2	2718	MCE, DUF3407
3	2026	MCE, MCE, MCE
4	1042	MCE, MCE, MCE, MCE, MCE, MCE, MCE
5	236	MCE, MCE, MCE, MCE, MCE, MCE
6	125	MCE, MCE
7	35	MCE, DUF330
8	20	MCE, MCE, MCE, MCE
9	15	MCE, MCE, MCE, MCE, MCE
10	8	MCE, MCE, DUF3407
11	7	MCE, MCE, MCE, MCE, MCE, MCE, MCE, MCE
12	4	MCE, DUF3407, MCE
13	4	MCE, MCE, MCE, DUF330
14	2	MCE, AP2
15	2	PqiA, PqiA, MCE, MCE, MCE, DUF330
16	2	PqiA, MCE, MCE
17	2	MCE, PLU-1
18	2	MCE, OmpA
19	1	MCE, MCE, MCE, MCE, Molybdopterin
20	1	PqiA, PqiA, MCE, MCE, MCE, MCE, MCE, MCE, MCE, Nol1_Nop2_Fmu, Methyltransf_31, Methyltransf_18, Methyltransf_26, Nol1_Nop2_Fmu, Nol1_Nop2_Fmu_2
21	1	MCE, MCE, MCE, MCE, MCE, MCE, MCE, MCE, MCE, MCE, MCE, MCE, MCE
22	1	MCE, DUF4455
23	1	OTCace_N, OTCace, MCE
24	1	DUF3407, MCE
25	1	MCE, Inositol_P

26	1	Permease, MCE, Tol_Tol_Ttg2, STAS_2, BolA
27	1	MCE, Bacillus_HBL
28	1	MCE, RTX
29	1	MCE, Serine_rich
30	1	MCE, MCE, MCE, MCE, PqiA, MCE, MCE, MCE
31	1	MCE, DUF3407, MCE, DUF3407
32	1	MCE, DUF3407, DUF3407
33	1	PqiA, MCE, MCE, MCE, MCE, MCE, Nol1_Nop2_Fmu, Nol1_Nop2_Fmu_2
34	1	MCE, LXG
35	1	MCE, GCN5L1
36	1	ABC_tran, MCE
37	1	NDUFA12, MCE, DUF2155
38	1	MreC, MCE, MCE

Supplementary table S2: IDs for proteins used in cluster analysis

Uniprot ID	Arch	Phylum	Class	Species
tr|W7QLA0|W7QLA0_9ALTE	3	Proteobacteria	Gammaproteobacteria	Catenovulum agarivorans
tr|Q1JX22|Q1JX22_DESAC	3	Proteobacteria	Deltaproteobacteria	Desulfuromonas acetoxidans
tr|Q2BQQ8|Q2BQQ8_NEPCE	3	Proteobacteria	Gammaproteobacteria	Neptuniibacter caesariensis
tr|B5JP82|B5JP82_9BACT	3	Verrucomicrobia	Verrucomicrobiae	Verrucomicrobiae bacterium DG1235
tr|C4L8N7|C4L8N7_TOLAT	3	Proteobacteria	Gammaproteobacteria	Tolumonas auensis
tr|Q0HUV4|Q0HUV4_SHESR	3	Proteobacteria	Gammaproteobacteria	Shewanella sp. MR-7
tr|R1GTU0|R1GTU0_9GAMM	3	Proteobacteria	Gammaproteobacteria	Grimontia indica
tr|G2J562|G2J562_PSEUL	3	Proteobacteria	Betaproteobacteria	Pseudogulbenkiania sp. NH8B
tr|K1IQ67|K1IQ67_9GAMM	3	Proteobacteria	Gammaproteobacteria	Aeromonas veronii
tr|A0A085APC5|A0A085APC5_9GAMM	3	Proteobacteria	Gammaproteobacteria	Halomonas sp. KO116
tr|A0A090PG68|A0A090PG68_9VIBR	3	Proteobacteria	Gammaproteobacteria	Vibrio ponticus
tr|I9P2X1|I9P2X1_9ALTE	3	Proteobacteria	Gammaproteobacteria	Alishewanella agri
tr|D4ZEJ0|D4ZEJ0_SHEVD	3	Proteobacteria	Gammaproteobacteria	Shewanella violacea
tr|A3Y6F5|A3Y6F5_9GAMM	3	Proteobacteria	Gammaproteobacteria	Marinomonas sp. MED121
tr|W1RZL0|W1RZL0_9GAMM	3	Proteobacteria	Gammaproteobacteria	Marinomonas profundimaris
tr|A0A084IRA0|A0A084IRA0_9GAMM	3	Proteobacteria	Gammaproteobacteria	Salinisphaera hydrothermalis
tr|F7NS36|F7NS36_9GAMM	3	Proteobacteria	Gammaproteobacteria	Rheinheimera sp. A13L
tr|D8KBC3|D8KBC3_NITWC	3	Proteobacteria	Gammaproteobacteria	Nitrosococcus watsonii
tr|K2K842|K2K842_9GAMM	3	Proteobacteria	Gammaproteobacteria	Idiomarina xiamenensis
tr|B4WYV8|B4WYV8_9GAMM	3	Proteobacteria	Gammaproteobacteria	Alcanivorax sp. DG881
tr|Q47ZJ5|Q47ZJ5_COLP3	3	Proteobacteria	Gammaproteobacteria	Colwellia psychrerythraea
tr|K6XBP4|K6XBP4_9ALTE	3	Proteobacteria	Gammaproteobacteria	Glaciecola arctica
tr|A0A0A0DWT9|A0A0A0DWT9_9SPIO	3	Spirochaetes	Spirochaetia	Spirochaeta sp. JC202
tr|H5TE19|H5TE19_9ALTE	3	Proteobacteria	Gammaproteobacteria	Glaciecola punicea

tr|A0LD19|A0LD19_MAGSM	3	Proteobacteria	Alphaproteobacteria	Magnetococcus marinus
tr|A3UYZ0|A3UYZ0_VIBSP	3	Proteobacteria	Gammaproteobacteria	Vibrio splendidus
tr|A0A066TDG6|A0A066TDG6_9GAMM	3	Proteobacteria	Gammaproteobacteria	Gilliamella apicola
tr|Q5QYR9|Q5QYR9_IDILO	3	Proteobacteria	Gammaproteobacteria	Idiomarina loihiensis
tr|A4C4S4|A4C4S4_9GAMM	3	Proteobacteria	Gammaproteobacteria	Pseudoalteromonas tunicata
tr|A0A0A0DA86|A0A0A0DA86_9PROT	3	Proteobacteria	Alphaproteobacteria	Inquilinus limosus
tr|I3Y552|I3Y552_THIV6	3	Proteobacteria	Gammaproteobacteria	Thiocystis violascens
tr|Q21EW5|Q21EW5_SACD2	3	Proteobacteria	Gammaproteobacteria	Saccharophagus degradans
tr|G8APC9|G8APC9_AZOBR	3	Proteobacteria	Alphaproteobacteria	Azospirillum brasilense
tr|F1VUW1|F1VUW1_9BURK	3	Proteobacteria	Betaproteobacteria	Oxalobacteraceae bacterium IMCC9480
tr|K0EE59|K0EE59_ALTMB	3	Proteobacteria	Gammaproteobacteria	Alteromonas macleodii
tr|A0A077ZKM6|A0A077ZKM6_TRITR	15	Nematoda	Enoplea	Trichuris trichiura
tr|A0A074W1C1|A0A074W1C1_9NEIS	3	Proteobacteria	Betaproteobacteria	Snodgrassella alvi
tr|V9HKU2|V9HKU2_9NEIS	3	Proteobacteria	Betaproteobacteria	Simonsiella muelleri
tr|Q0EYY4|Q0EYY4_9PROT	3	Proteobacteria	Zetaproteobacteria	Mariprofundus ferrooxydans
tr|G8QKZ3|G8QKZ3_AZOSU	3	Proteobacteria	Betaproteobacteria	Azospira oryzae
tr|I3BPC0|I3BPC0_9GAMM	3	Proteobacteria	Gammaproteobacteria	Thiothrix nivea
tr|C8NBJ5|C8NBJ5_9GAMM	3	Proteobacteria	Gammaproteobacteria	Cardiobacterium hominis
tr|F5RGX9|F5RGX9_METUF	3	Proteobacteria	Betaproteobacteria	Methyloversatilis universalis
tr|A0A0C1EHH8|A0A0C1EHH8_9NEIS	3	Proteobacteria	Betaproteobacteria	Chromobacterium piscinae
tr|U1KL45|U1KL45_9GAMM	3	Proteobacteria	Gammaproteobacteria	Pseudoalteromonas rubra
tr|H8YXD3|H8YXD3_9GAMM	3	Proteobacteria	Gammaproteobacteria	Thiorhodovibrio sp. 970
tr|Q60BV2|Q60BV2_METCA	3	Proteobacteria	Gammaproteobacteria	Methylococcus capsulatus
tr|A0A0C2BZI7|A0A0C2BZI7_9BURK	3	Proteobacteria	Betaproteobacteria	Burkholderia sp. MR1
tr|V5C6C1|V5C6C1_9GAMM	3	Proteobacteria	Gammaproteobacteria	Methyloglobulus morosus
tr|W0TPY7|W0TPY7_9GAMM	3	Proteobacteria	Gammaproteobacteria	Thiolapillus brandeum
tr|K6ZJ07|K6ZJ07_9ALTE	3	Proteobacteria	Gammaproteobacteria	Glaciecola pallidula

tr|H8FSC4|H8FSC4_PHAMO	3	Proteobacteria	Alphaproteobacteria	Phaeospirillum molischianum
tr|A0A0A0XK37|A0A0A0XK37_BORPT	3	Proteobacteria	Betaproteobacteria	Bordetella pertussis
tr|A0A074JU64|A0A074JU64_9PROT	3	Proteobacteria	Alphaproteobacteria	Thalassospira permensis
tr|C7LVB3|C7LVB3_DESBD	3	Proteobacteria	Deltaproteobacteria	Desulfomicrobium baculatum
tr|A0A085VDL2|A0A085VDL2_PSESX	3	Proteobacteria	Gammaproteobacteria	Pseudomonas syringae
tr|A4END7|A4END7_9RHOB	3	Proteobacteria	Alphaproteobacteria	Roseobacter sp. CCS2
tr|F6G519|F6G519_RALS8	3	Proteobacteria	Betaproteobacteria	Ralstonia solanacearum
tr|A0A0A2NAS5|A0A0A2NAS5_ALCFA	3	Proteobacteria	Betaproteobacteria	Alcaligenes faecalis
tr|E7RVG0|E7RVG0_9BURK	3	Proteobacteria	Betaproteobacteria	Lautropia mirabilis
tr|A2SBR3|A2SBR3_METPP	3	Proteobacteria	Betaproteobacteria	Methylibium petroleiphilum
tr|W4LZ37|W4LZ37_9DELT	3	Proteobacteria	Deltaproteobacteria	Candidatus Entotheonella sp. TSY1
tr|D3P1L3|D3P1L3_AZOS1	3	Proteobacteria	Alphaproteobacteria	Azospirillum lipoferum
tr|L0GTT0|L0GTT0_9GAMM	3	Proteobacteria	Gammaproteobacteria	Thioflavicoccus mobilis
tr|W0P9Z5|W0P9Z5_9BURK	3	Proteobacteria	Betaproteobacteria	Advenella mimigardefordensis
tr|A0A011MTG9|A0A011MTG9_9PROT	3	Proteobacteria	Betaproteobacteria	Candidatus Accumulibacter sp. SK-12
tr|W8X4D4|W8X4D4_CASDE	3	Proteobacteria	Betaproteobacteria	Castellaniella defragrans
tr|E6X0T4|E6X0T4_NITSE	3	Proteobacteria	Epsilonproteobacteria	Nitratifractor salsuginis
tr|H5WFA8|H5WFA8_RALSL	3	Proteobacteria	Betaproteobacteria	Ralstonia solanacearum
tr|N9J529|N9J529_ACIBA	3	Proteobacteria	Gammaproteobacteria	Acinetobacter baumannii
tr|E8WLI1|E8WLI1_GEOS8	3	Proteobacteria	Deltaproteobacteria	Geobacter sp. M18
tr|I4W5L9|I4W5L9_9GAMM	3	Proteobacteria	Gammaproteobacteria	Rhodanobacter spathiphylli
tr|H8Z2C9|H8Z2C9_9GAMM	3	Proteobacteria	Gammaproteobacteria	Thiorhodovibrio sp. 970
tr|A5EVN5|A5EVN5_DICNV	3	Proteobacteria	Gammaproteobacteria	Dichelobacter nodosus
tr|U6ZHU6|U6ZHU6_9ENTR	3	Proteobacteria	Gammaproteobacteria	Dickeya solani
tr|N6VSY4|N6VSY4_9ALTE	3	Proteobacteria	Gammaproteobacteria	Marinobacter nanhaiticus
tr|I7ZD67|I7ZD67_9GAMM	3	Proteobacteria	Gammaproteobacteria	Hydrocarboniphaga effusa
tr|D5RJN2|D5RJN2_9PROT	3	Proteobacteria	Alphaproteobacteria	Roseomonas cervicalis

tr|S9QUJ4|S9QUJ4_9RHOB	3	Proteobacteria	Alphaproteobacteria	Salipiger mucosus
tr|K5YNK1|K5YNK1_9PROT	3	Proteobacteria	Alphaproteobacteria	Acidocella sp. MX-AZ02
tr|W6IEP2|W6IEP2_9PROT	3	Proteobacteria	Alphaproteobacteria	Granulibacter bethesdensis
tr|K2HDV7|K2HDV7_9RHOB	3	Proteobacteria	Alphaproteobacteria	Oceaniovalibus guishaninsula
tr|E8LJ51|E8LJ51_9GAMM	3	Proteobacteria	Gammaproteobacteria	Succinatimonas hippei
tr|A3VJY1|A3VJY1_9RHOB	3	Proteobacteria	Alphaproteobacteria	Maritimibacter alkaliphilus
tr|A0A0A1HRZ9|A0A0A1HRZ9_9PSED	3	Proteobacteria	Gammaproteobacteria	Pseudomonas sp. SHC52
tr|F1YR40|F1YR40_9PROT	3	Proteobacteria	Alphaproteobacteria	Acetobacter pomorum
tr|E0XV80|E0XV80_9RHOB	3	Proteobacteria	Alphaproteobacteria	uncultured Rhodobacterales bacterium
tr|A0A0A0HMQ5|A0A0A0HMQ5_9RHOB	3	Proteobacteria	Alphaproteobacteria	Roseovarius mucosus
tr|A4SZ70|A4SZ70_POLSQ	3	Proteobacteria	Betaproteobacteria	Polynucleobacter necessarius
tr|A0A095DJU8|A0A095DJU8_BURCE	3	Proteobacteria	Betaproteobacteria	Burkholderia cepacia
tr|I4N3T3|I4N3T3_9PSED	3	Proteobacteria	Gammaproteobacteria	Pseudomonas sp. M47T1
tr|C7DF26|C7DF26_9RHOB	3	Proteobacteria	Alphaproteobacteria	Thalassobium sp. R2A62
tr|E3F4J9|E3F4J9_KETVY	3	Proteobacteria	Alphaproteobacteria	Ketogulonicigenium vulgare
tr|B5WKB9|B5WKB9_9BURK	3	Proteobacteria	Betaproteobacteria	Burkholderia sp. H160
tr|W4HJ72|W4HJ72_9RHOB	3	Proteobacteria	Alphaproteobacteria	Roseivivax atlanticus
tr|Q5LU11|Q5LU11_RUEPO	3	Proteobacteria	Alphaproteobacteria	Ruegeria pomeroyi
tr|S9R3A0|S9R3A0_9RHOB	3	Proteobacteria	Alphaproteobacteria	Rubellimicrobium thermophilum
tr|Q2CEX1|Q2CEX1_OCEGH	3	Proteobacteria	Alphaproteobacteria	Oceanicola granulosus
tr|A0A0A0EJW2|A0A0A0EJW2_9RHOB	3	Proteobacteria	Alphaproteobacteria	Oceanicola sp. 22II-S11g
tr|A1B5I5|A1B5I5_PARDP	3	Proteobacteria	Alphaproteobacteria	Paracoccus denitrificans
tr|A6Q8Y6|A6Q8Y6_SULNB	3	Proteobacteria	Epsilonproteobacteria	Sulfurovum sp. NBC37-1
tr|R5EUI6|R5EUI6_9GAMM	3	Proteobacteria	Gammaproteobacteria	Succinatimonas sp. CAG:777
tr|E0MTJ0|E0MTJ0_9RHOB	3	Proteobacteria	Alphaproteobacteria	Ahrensia sp. R2A130
tr|Q28S24|Q28S24_JANSC	3	Proteobacteria	Alphaproteobacteria	Jannaschia sp. CCS1
tr|A0A074JYA3|A0A074JYA3_9RHOB	3	Proteobacteria	Alphaproteobacteria	Thioclava pacifica

tr|I2JLN1|I2JLN1_9GAMM	5	Proteobacteria	Gammaproteobacteria	gamma proteobacterium BDW918
tr|B1ZMG9|B1ZMG9_OPITP	6	Verrucomicrobia	Opitutae	Opitutus terrae
tr|A3SUA2|A3SUA2_9RHOB	3	Proteobacteria	Alphaproteobacteria	Sulfitobacter sp. NAS-14.1
tr|A0A086YBP6|A0A086YBP6_9RHOB	3	Proteobacteria	Alphaproteobacteria	Haematobacter massiliensis
tr|A0A017HC32|A0A017HC32_9RHOB	3	Proteobacteria	Alphaproteobacteria	Loktanella hongkongensis
tr|I6AE33|I6AE33_BURTH	3	Proteobacteria	Betaproteobacteria	Burkholderia thailandensis
tr|A0A017HNY4|A0A017HNY4_9RHOB	13	Proteobacteria	Alphaproteobacteria	Rubellimicrobium mesophilum
tr|A0A0C2VS99|A0A0C2VS99_9PROT	3	Proteobacteria	Epsilonproteobacteria	Sulfurovum sp. PC08-66
tr|A0A081G1V5|A0A081G1V5_9ALTE	4	Proteobacteria	Gammaproteobacteria	Marinobacterium sp. AK27
tr|R9PQD7|R9PQD7_AGAAL	4	Proteobacteria	Gammaproteobacteria	Agarivorans albus
tr|Q12NH6|Q12NH6_SHEDO	4	Proteobacteria	Gammaproteobacteria	Shewanella denitrificans
tr|W9VNQ3|W9VNQ3_9GAMM	9	Proteobacteria	Gammaproteobacteria	Nitrincola sp. AK23
tr|E1SSP2|E1SSP2_FERBD	4	Proteobacteria	Gammaproteobacteria	Ferrimonas balearica
tr|B5JKB5|B5JKB5_9BACT	6	Verrucomicrobia	Verrucomicrobiae	Verrucomicrobiae bacterium DG1235
tr|F9ZUH5|F9ZUH5_ACICS	3	Proteobacteria	Gammaproteobacteria	Acidithiobacillus caldus
tr|A0A094K1R7|A0A094K1R7_9GAMM	4	Proteobacteria	Gammaproteobacteria	Shewanella sp. YQH10
tr|A0A0C2YWZ5|A0A0C2YWZ5_9PROT	3	Proteobacteria	Epsilonproteobacteria	Sulfurovum sp. AS07-7
tr|A0A091ADE0|A0A091ADE0_AERSA	4	Proteobacteria	Gammaproteobacteria	Aeromonas salmonicida
tr|A0A0C2UYM8|A0A0C2UYM8_9PROT	3	Proteobacteria	Epsilonproteobacteria	Sulfurovum sp. FS06-10
tr|F7S7F1|F7S7F1_9PROT	3	Proteobacteria	Alphaproteobacteria	Acidiphilium sp. PM
tr|Q0VLU5|Q0VLU5_ALCBS	6	Proteobacteria	Gammaproteobacteria	Alcanivorax borkumensis
tr|M1PHR4|M1PHR4_DESSD	8	Proteobacteria	Deltaproteobacteria	Desulfocapsa sulfexigens
tr|D1P4W6|D1P4W6_9ENTR	5	Proteobacteria	Gammaproteobacteria	Providencia rustigianii
tr|Q7MKY5|Q7MKY5_VIBVY	4	Proteobacteria	Gammaproteobacteria	Vibrio vulnificus
tr|Q65UD6|Q65UD6_MANSM	4	Proteobacteria	Gammaproteobacteria	[Mannheimia] succiniciproducens
tr|R1IEJ1|R1IEJ1_9GAMM	4	Proteobacteria	Gammaproteobacteria	Grimontia indica
tr|A1SWU9|A1SWU9_PSYIN	4	Proteobacteria	Gammaproteobacteria	Psychromonas ingrahamii

tr|A0A099L1N4|A0A099L1N4_9GAMM	11	Proteobacteria	Gammaproteobacteria	Thalassotalea sp. ND16A
tr|L6NP03|L6NP03_SALEN	19	Proteobacteria	Gammaproteobacteria	Salmonella enterica
tr|Q6AQF6|Q6AQF6_DESPS	5	Proteobacteria	Deltaproteobacteria	Desulfotalea psychrophila
tr|E1VMF2|E1VMF2_9GAMM	5	Proteobacteria	Gammaproteobacteria	gamma proteobacterium HdN1
tr|M9YIV7|M9YIV7_AZOVI	5	Proteobacteria	Gammaproteobacteria	Azotobacter vinelandii
tr|A0A081NCZ7|A0A081NCZ7_9GAMM	5	Proteobacteria	Gammaproteobacteria	Endozoicomonas numazuensis
tr|A0A0A2VIN0|A0A0A2VIN0_BEABA	20	Ascomycota	Sordariomycetes	Beauveria bassiana
tr|K8BKY4|K8BKY4_9ENTR	30	Proteobacteria	Gammaproteobacteria	Cronobacter turicensis
tr|T0CD20|T0CD20_9DELT	8	Proteobacteria	Deltaproteobacteria	Bacteriovorax sp. BAL6_X
tr|A0A0A2I518|A0A0A2I518_9DELT	4	Proteobacteria	Deltaproteobacteria	Desulfobulbus sp. Tol-SR
tr|K5YSZ8|K5YSZ8_9PROT	3	Proteobacteria	Alphaproteobacteria	Acidocella sp. MX-AZ02
tr|E8RBT1|E8RBT1_DESPD	4	Proteobacteria	Deltaproteobacteria	Desulfobulbus propionicus
tr|A6FIP2|A6FIP2_9GAMM	4	Proteobacteria	Gammaproteobacteria	Moritella sp. PE36
tr|D4H238|D4H238_DENA2	11	Deferribacteres	Deferribacteres	Denitrovibrio acetiphilus
tr|A6DBM5|A6DBM5_9PROT	3	Proteobacteria	Epsilonproteobacteria	Caminibacter mediatlanticus
tr|Q6MNT4|Q6MNT4_BDEBA	6	Proteobacteria	Deltaproteobacteria	Bdellovibrio bacteriovorus
tr|M4V5W8|M4V5W8_9DELT	6	Proteobacteria	Deltaproteobacteria	Bdellovibrio exovorus
tr|Q48A65|Q48A65_COLP3	11	Proteobacteria	Gammaproteobacteria	Colwellia psychrerythraea
tr|A6DM96|A6DM96_9BACT	4	Lentisphaerae	Lentisphaeria	Lentisphaera araneosa
tr|K2K470|K2K470_9GAMM	5	Proteobacteria	Gammaproteobacteria	Gallaecimonas xiamenensis
tr|M5TMN6|M5TMN6_9PLAN	6	Planctomycetes	Planctomycetia	Rhodopirellula sp. SWK7
tr|M5RZ87|M5RZ87_9PLAN	6	Planctomycetes	Planctomycetia	Rhodopirellula maiorica
tr|E8R5N3|E8R5N3_ISOPI	16	Planctomycetes	Planctomycetia	Isosphaera pallida
tr|V4KT63|V4KT63_9DELT	4	Proteobacteria	Deltaproteobacteria	uncultured Desulfofustis sp. PB-SRB1
tr|W0Z5H5|W0Z5H5_PSEAI	25	Proteobacteria	Gammaproteobacteria	Pseudomonas aeruginosa
tr|G2HXQ5|G2HXQ5_9PROT	4	Proteobacteria	Epsilonproteobacteria	Arcobacter sp. L
tr|K1YAB1|K1YAB1_9BACT	3			uncultured bacterium

tr|I3CHA5|I3CHA5_9GAMM	6	Proteobacteria	Gammaproteobacteria	Beggiatoa alba
tr|I4WAE0|I4WAE0_9GAMM	1	Proteobacteria	Gammaproteobacteria	Rhodanobacter sp. 115
tr|E5Y3N4|E5Y3N4_BILWA	1	Proteobacteria	Deltaproteobacteria	Bilophila wadsworthia
tr|U2ZN81|U2ZN81_PSEAC	1	Proteobacteria	Gammaproteobacteria	Pseudomonas alcaligenes
tr|X1WKZ8|X1WKZ8_ACYPI	33	Arthropoda	Insecta	Acyrthosiphon pisum
tr|A0A0B8NYA0|A0A0B8NYA0_9VIBR	6	Proteobacteria	Gammaproteobacteria	Vibrio sp. JCM 19231
tr|W7DYQ2|W7DYQ2_9PROT	1	Proteobacteria	Alphaproteobacteria	Commensalibacter sp. MX01
tr|A0A090RQ91|A0A090RQ91_9VIBR	6	Proteobacteria	Gammaproteobacteria	Vibrio sp. C7
tr|R5QLU3|R5QLU3_9PROT	1	Proteobacteria	Alphaproteobacteria	Acetobacter sp. CAG:977
tr|T2G944|T2G944_DESGI	1	Proteobacteria	Deltaproteobacteria	Desulfovibrio gigas
tr|A0A059KS25|A0A059KS25_9BURK	1	Proteobacteria	Betaproteobacteria	Sphaerotilus natans
tr|D8F981|D8F981_9DELT	1	Proteobacteria	Deltaproteobacteria	delta proteobacterium NaphS2
tr|A0A0A7XF72|A0A0A7XF72_ACIBA	1	Proteobacteria	Gammaproteobacteria	Acinetobacter baumannii
tr|A0A0B0HD74|A0A0B0HD74_SOVGS	1	Proteobacteria	Gammaproteobacteria	Solemya velum gill symbiont
tr|R5EIQ1|R5EIQ1_9GAMM	1	Proteobacteria	Gammaproteobacteria	Succinatimonas sp. CAG:777
tr|E5APT9|E5APT9_BURRH	1	Proteobacteria	Betaproteobacteria	Burkholderia rhizoxinica
tr|Y0KET7|Y0KET7_9PROT	1	Proteobacteria	Betaproteobacteria	Methylophilaceae bacterium 11
tr|K2BJ74|K2BJ74_9BACT	1			uncultured bacterium
tr|A0A084IL35|A0A084IL35_9GAMM	1	Proteobacteria	Gammaproteobacteria	Salinisphaera hydrothermalis
tr|Q0A6H4|Q0A6H4_ALKEH	1	Proteobacteria	Gammaproteobacteria	Alkalilimnicola ehrlichii
tr|M1LXJ0|M1LXJ0_9PROT	1	Proteobacteria	Betaproteobacteria	Candidatus Kinetoplastibacterium galatii
tr|A0A077ZFB4|A0A077ZFB4_TRITR	26	Nematoda	Enoplea	Trichuris trichiura
tr|L0DYZ8|L0DYZ8_THIND	1	Proteobacteria	Gammaproteobacteria	Thioalkalivibrio nitratireducens
tr|A0A089WLJ1|A0A089WLJ1_9PSED	1	Proteobacteria	Gammaproteobacteria	Pseudomonas cremoricolorata
tr|K2BX90|K2BX90_9BACT	1			uncultured bacterium
tr|S0G4N9|S0G4N9_9DELT	1	Proteobacteria	Deltaproteobacteria	Desulfotignum phosphitoxidans
tr|Q6AKW0|Q6AKW0_DESPS	1	Proteobacteria	Deltaproteobacteria	Desulfotalea psychrophila

tr|A0A090IGN5|A0A090IGN5_9GAMM	1	Proteobacteria	Gammaproteobacteria	Moritella viscosa
tr|Q1K499|Q1K499_DESAC	1	Proteobacteria	Deltaproteobacteria	Desulfuromonas acetoxidans
tr|I4B9F0|I4B9F0_TURPD	1	Spirochaetes	Spirochaetia	Turneriella parva
tr|C7LQ95|C7LQ95_DESBD	1	Proteobacteria	Deltaproteobacteria	Desulfomicrobium baculatum
tr|A0LFA1|A0LFA1_SYNFM	1	Proteobacteria	Deltaproteobacteria	Syntrophobacter fumaroxidans
tr|E8RF78|E8RF78_DESPD	1	Proteobacteria	Deltaproteobacteria	Desulfobulbus propionicus
tr|L8XXI9|L8XXI9_9GAMM	1	Proteobacteria	Gammaproteobacteria	Wohlfahrtiimonas chitiniclastica
tr|B5JEH5|B5JEH5_9BACT	1	Verrucomicrobia	Verrucomicrobiae	Verrucomicrobiae bacterium DG1235
tr|Q2LQ52|Q2LQ52_SYNAS	1	Proteobacteria	Deltaproteobacteria	Syntrophus aciditrophicus
tr|Q1Q2A0|Q1Q2A0_9BACT	1	Planctomycetes	Planctomycetia	Candidatus Kuenenia stuttgartiensis
tr|F2NCT0|F2NCT0_DESAR	1	Proteobacteria	Deltaproteobacteria	Desulfobacca acetoxidans
tr|Q07WT5|Q07WT5_SHEFN	1	Proteobacteria	Gammaproteobacteria	Shewanella frigidimarina
tr|A0A059ZYB4|A0A059ZYB4_9GAMM	1	Proteobacteria	Gammaproteobacteria	Acidithiobacillus caldus
tr|A0LFH2|A0LFH2_SYNFM	1	Proteobacteria	Deltaproteobacteria	Syntrophobacter fumaroxidans
tr|Q0F1F6|Q0F1F6_9PROT	1	Proteobacteria	Zetaproteobacteria	Mariprofundus ferrooxydans
tr|A0A0A6R484|A0A0A6R484_9GAMM	1	Proteobacteria	Gammaproteobacteria	Methylomonas denitrificans
tr|I6ANI7|I6ANI7_9BACT	1	Verrucomicrobia	Opitutae	Opitutaceae bacterium TAV1
tr|K7YS43|K7YS43_9PROT	1	Proteobacteria	Alphaproteobacteria	Candidatus Endolissoclinum faulkneri
tr|K2BPX9|K2BPX9_9BACT	1			uncultured bacterium
tr|G2DCT6|G2DCT6_9GAMM	1	Proteobacteria	Gammaproteobacteria	endosymbiont of Riftia pachyptila
tr|A0A075WT33|A0A075WT33_9BACT	1	Thermodesulfobacteria	Thermodesulfobacteria	Thermodesulfobacterium commune
tr|A0A077AZG9|A0A077AZG9_9RICK	1	Proteobacteria	Alphaproteobacteria	Candidatus Paracaedibacter acanthamoebae
tr|A0A090ANW3|A0A090ANW3_9GAMM	1	Proteobacteria	Gammaproteobacteria	Thioploca ingrica
tr|K5YWE0|K5YWE0_9PROT	1	Proteobacteria	Alphaproteobacteria	Acidocella sp. MX-AZ02
tr|D6STH6|D6STH6_9DELT	1	Proteobacteria	Deltaproteobacteria	Desulfonatronospira thiodismutans
tr|I7ZC47|I7ZC47_9GAMM	1	Proteobacteria	Gammaproteobacteria	Hydrocarboniphaga effusa
tr|F8A8S9|F8A8S9_THEID	1	Thermodesulfobacteria	Thermodesulfobacteria	Thermodesulfatator indicus

tr|A0A077FLA7|A0A077FLA7_9RICK	1	Proteobacteria	Alphaproteobacteria	Rickettsiales bacterium Ac37b
tr|A0A0B8UFY6|A0A0B8UFY6_PISSA	1	Proteobacteria	Gammaproteobacteria	Piscirickettsia salmonis
tr|A8EXT9|A8EXT9_RICCK	1	Proteobacteria	Alphaproteobacteria	Rickettsia canadensis
tr|W0JKP3|W0JKP3_DESAE	1	Proteobacteria	Deltaproteobacteria	Desulfurella acetivorans
tr|C8ND21|C8ND21_9GAMM	1	Proteobacteria	Gammaproteobacteria	Cardiobacterium hominis
tr|A5EYC2|A5EYC2_DICNV	6	Proteobacteria	Gammaproteobacteria	Dichelobacter nodosus
tr|M1LLF7|M1LLF7_9PROT	1	Proteobacteria	Betaproteobacteria	Candidatus Kinetoplastibacterium desouzaii
tr|I3TX61|I3TX61_TISMK	1	Proteobacteria	Alphaproteobacteria	Tistrella mobilis
tr|Q2A4R8|Q2A4R8_FRATH	1	Proteobacteria	Gammaproteobacteria	Francisella tularensis
tr|H8L3U9|H8L3U9_FRAAD	6	Proteobacteria	Gammaproteobacteria	Frateuria aurantia
tr|K2J1F9|K2J1F9_9PROT	1	Proteobacteria	Alphaproteobacteria	Oceanibaculum indicum
tr|D5QBK8|D5QBK8_KOMHA	1	Proteobacteria	Alphaproteobacteria	Komagataeibacter hansenii
tr|A0A0A8F5E9|A0A0A8F5E9_NEIME	1	Proteobacteria	Betaproteobacteria	Neisseria meningitidis
tr|D5MK82|D5MK82_9BACT	1			Candidatus Methylomirabilis oxyfera
tr|W2V2P6|W2V2P6_9RICK	1	Proteobacteria	Alphaproteobacteria	Candidatus Xenolissoclinum pacificiensis
tr|Q0FAL5|Q0FAL5_9RHOB	1	Proteobacteria	Alphaproteobacteria	Rhodobacterales bacterium HTCC2255
tr|Q2LS06|Q2LS06_SYNAS	1	Proteobacteria	Deltaproteobacteria	Syntrophus aciditrophicus
tr|K2E4C8|K2E4C8_9BACT	1			uncultured bacterium
tr|A3UD49|A3UD49_9RHOB	1	Proteobacteria	Alphaproteobacteria	Oceanicaulis sp. HTCC2633
tr|A0A0C1QJC1|A0A0C1QJC1_9RICK	1	Proteobacteria	Alphaproteobacteria	endosymbiont of Acanthamoeba sp. UWC36
tr|B3CMK7|B3CMK7_WOLPP	1	Proteobacteria	Alphaproteobacteria	Wolbachia endosymbiont of Culex quinquefasciatus
tr|W0DXE2|W0DXE2_9GAMM	1	Proteobacteria	Gammaproteobacteria	Thioalkalimicrobium aerophilum
tr|A0A061QFC6|A0A061QFC6_9PROT	1	Proteobacteria	Alphaproteobacteria	alpha proteobacterium Q-1
tr|A0A086YDD8|A0A086YDD8_9RHOB	1	Proteobacteria	Alphaproteobacteria	Haematobacter missouriensis
tr|T0ZPG4|T0ZPG4_9ZZZZ	1			mine drainage metagenome
tr|S5YB63|S5YB63_PARAH	1	Proteobacteria	Alphaproteobacteria	Paracoccus aminophilus
tr|R5QNA7|R5QNA7_9PROT	1	Proteobacteria	Alphaproteobacteria	Acetobacter sp. CAG:977

tr|S9QYP4|S9QYP4_9RHOB	1	Proteobacteria	Alphaproteobacteria	Rubellimicrobium thermophilum
tr|M4VEA5|M4VEA5_9DELT	1	Proteobacteria	Deltaproteobacteria	Bdellovibrio exovorus
tr|C8X0M3|C8X0M3_DESRD	1	Proteobacteria	Deltaproteobacteria	Desulfohalobium retbaense
tr|G9ZVS6|G9ZVS6_9PROT	1	Proteobacteria	Alphaproteobacteria	Acetobacteraceae bacterium AT-5844
tr|G2I4S7|G2I4S7_KOMMN	1	Proteobacteria	Alphaproteobacteria	Komagataeibacter medellinensis
tr|A0A077C2N2|A0A077C2N2_9RICK	1	Proteobacteria	Alphaproteobacteria	Candidatus Caedibacter acanthamoebae
tr|D1AD49|D1AD49_THECD	2	Actinobacteria	Actinobacteria	Thermomonospora curvata
tr|D6KSC1|D6KSC1_9FIRM	1	Firmicutes	Negativicutes	Veillonella sp. 6_1_27
tr|Q5H658|Q5H658_XANOR	1	Proteobacteria	Gammaproteobacteria	Xanthomonas oryzae
tr|R5R5J3|R5R5J3_9PROT	1	Proteobacteria		Proteobacteria bacterium CAG:495
tr|B9KHI7|B9KHI7_ANAMF	1	Proteobacteria	Alphaproteobacteria	Anaplasma marginale
tr|Q2W3M7|Q2W3M7_MAGSA	1	Proteobacteria	Alphaproteobacteria	Magnetospirillum magneticum
tr|A0A078L431|A0A078L431_9GAMM	1	Proteobacteria	Gammaproteobacteria	Legionella massiliensis
tr|H8FPR4|H8FPR4_PHAMO	1	Proteobacteria	Alphaproteobacteria	Phaeospirillum molischianum
tr|B5YIL8|B5YIL8_THEYD	1	Nitrospirae	Nitrospira	Thermodesulfovibrio yellowstonii
tr|A5CEP7|A5CEP7_ORITB	1	Proteobacteria	Alphaproteobacteria	Orientia tsutsugamushi
tr|O66784|O66784_AQUAE	1	Aquificae	Aquificae	Aquifex aeolicus
tr|L8K1H1|L8K1H1_9BACT	1	Bacteroidetes	Cytophagia	Fulvivirga imtechensis
tr|W9AP53|W9AP53_9MYCO	2	Actinobacteria	Actinobacteria	Mycobacterium cosmeticum
tr|A0A059G889|A0A059G889_9RHOB	1	Proteobacteria	Alphaproteobacteria	Hyphomonas oceanitis
tr|Q5HC50|Q5HC50_EHRRW	1	Proteobacteria	Alphaproteobacteria	Ehrlichia ruminantium
tr|E1QG67|E1QG67_DESB2	1	Proteobacteria	Deltaproteobacteria	Desulfarculus baarsii
tr|F2NGX9|F2NGX9_DESAR	6	Proteobacteria	Deltaproteobacteria	Desulfobacca acetoxidans
tr|J2FZ00|J2FZ00_9CAUL	1	Proteobacteria	Alphaproteobacteria	Caulobacter sp. AP07
tr|D3PDB7|D3PDB7_DEFDS	1	Deferribacteres	Deferribacteres	Deferribacter desulfuricans
tr|A0A090VXL3|A0A090VXL3_9FLAO	1	Bacteroidetes	Flavobacteriia	Jejuia pallidilutea
tr|A8UTJ7|A8UTJ7_9AQUI	1	Aquificae	Aquificae	Hydrogenivirga sp. 128-5-R1-1

tr|X5GWN0|X5GWN0_9RICK	1	Proteobacteria	Alphaproteobacteria	Neorickettsia helminthoeca
tr|A0A0A0CYI9|A0A0A0CYI9_9PROT	1	Proteobacteria	Alphaproteobacteria	Inquilinus limosus
tr|M4S5I1|M4S5I1_9SPHN	1	Proteobacteria	Alphaproteobacteria	Sphingomonas sp. MM-1
tr|D5BSN9|D5BSN9_PUNMI	1	Proteobacteria	Alphaproteobacteria	Candidatus Puniceispirillum marinum
tr|F9N659|F9N659_9FIRM	1	Firmicutes	Negativicutes	Veillonella sp. oral taxon 780
tr|F1YQK0|F1YQK0_9PROT	1	Proteobacteria	Alphaproteobacteria	Acetobacter pomorum
tr|W5TFY4|W5TFY4_9NOCA	1	Actinobacteria	Actinobacteria	Nocardia nova
tr|Q0BRB9|Q0BRB9_GRABC	1	Proteobacteria	Alphaproteobacteria	Granulibacter bethesdensis
tr|U2WDD5|U2WDD5_9PROT	1	Proteobacteria	Alphaproteobacteria	alpha proteobacterium RS24
tr|G6EZ80|G6EZ80_9PROT	1	Proteobacteria	Alphaproteobacteria	Commensalibacter intestini
tr|R5QMG9|R5QMG9_9PROT	1	Proteobacteria		Proteobacteria bacterium CAG:495
tr|T0SGM9|T0SGM9_9DELT	1	Proteobacteria	Deltaproteobacteria	Bacteriovorax sp. Seq25_V
tr|F4CXJ4|F4CXJ4_PSEUX	2	Actinobacteria	Actinobacteria	Pseudonocardia dioxanivorans
tr|W7JBX5|W7JBX5_9PSEU	1	Actinobacteria	Actinobacteria	Actinokineospora sp. EG49
tr|A0A099DAR3|A0A099DAR3_9ACTO	2	Actinobacteria	Actinobacteria	Actinopolyspora erythraea
tr|A0A0C1P3T8|A0A0C1P3T8_9PSEU	2	Actinobacteria	Actinobacteria	Prauserella sp. Am3
tr|A0A0B7CJ46|A0A0B7CJ46_9PSEU	2	Actinobacteria	Actinobacteria	Kibdelosporangium sp. MJ126-NF4
tr|W7SR07|W7SR07_9PSEU	2	Actinobacteria	Actinobacteria	Kutzneria sp. 744
tr|J1S703|J1S703_9ACTO	2	Actinobacteria	Actinobacteria	Streptomyces auratus
tr|T2RPW9|T2RPW9_SACER	2	Actinobacteria	Actinobacteria	Saccharopolyspora erythraea
tr|H6RMU8|H6RMU8_BLASD	1	Actinobacteria	Actinobacteria	Blastococcus saxobsidens
tr|A0A0A0J9Q0|A0A0A0J9Q0_9MICO	1	Actinobacteria	Actinobacteria	Knoellia flava
tr|M3TSE7|M3TSE7_9ACTO	2	Actinobacteria	Actinobacteria	Gordonia paraffinivorans
tr|E2PY83|E2PY83_STRC2	2	Actinobacteria	Actinobacteria	Streptomyces clavuligerus
tr|G0FS99|G0FS99_AMYMS	1	Actinobacteria	Actinobacteria	Amycolatopsis mediterranei
tr|E2SGB0|E2SGB0_9ACTO	1	Actinobacteria	Actinobacteria	Aeromicrobium marinum
tr|R7WR74|R7WR74_9NOCA	2	Actinobacteria	Actinobacteria	Rhodococcus rhodnii

tr|E9V1A1|E9V1A1_9ACTO	1	Actinobacteria	Actinobacteria	Nocardioidaceae bacterium Broad-1
tr|D5UQ84|D5UQ84_TSUPD	1	Actinobacteria	Actinobacteria	Tsukamurella paurometabola
tr|A0A076N3Z6|A0A076N3Z6_AMYME	2	Actinobacteria	Actinobacteria	Amycolatopsis methanolica
tr|F6EHT4|F6EHT4_AMYSD	1	Actinobacteria	Actinobacteria	Amycolicicoccus subflavus
tr|E4WD60|E4WD60_RHOE1	2	Actinobacteria	Actinobacteria	Rhodococcus equi
tr|D3QB64|D3QB64_STANL	2	Actinobacteria	Actinobacteria	Stackebrandtia nassauensis
tr|A0A0A1DSY0|A0A0A1DSY0_NOCSI	1	Actinobacteria	Actinobacteria	Pimelobacter simplex
tr|D1A3A0|D1A3A0_THECD	2	Actinobacteria	Actinobacteria	Thermomonospora curvata
tr|A1SKI8|A1SKI8_NOCSJ	1	Actinobacteria	Actinobacteria	Nocardioides sp. JS614
tr|E9UWC5|E9UWC5_9ACTO	2	Actinobacteria	Actinobacteria	Nocardioidaceae bacterium Broad-1
tr|E2SEJ7|E2SEJ7_9ACTO	1	Actinobacteria	Actinobacteria	Aeromicrobium marinum
tr|L2TT79|L2TT79_9NOCA	2	Actinobacteria	Actinobacteria	Rhodococcus wratislaviensis
tr|X8B1E4|X8B1E4_MYCAV	2	Actinobacteria	Actinobacteria	Mycobacterium avium
tr|C1BB82|C1BB82_RHOOB	2	Actinobacteria	Actinobacteria	Rhodococcus opacus
tr|L7L5Y6|L7L5Y6_9ACTO	2	Actinobacteria	Actinobacteria	Gordonia hirsuta
tr|H0RGF1|H0RGF1_9ACTO	2	Actinobacteria	Actinobacteria	Gordonia polyisoprenivorans
tr|W8HMK3|W8HMK3_RHOOP	1	Actinobacteria	Actinobacteria	Rhodococcus opacus
tr|E2S874|E2S874_9ACTO	1	Actinobacteria	Actinobacteria	Aeromicrobium marinum
tr|R7XXX2|R7XXX2_9ACTO	1	Actinobacteria	Actinobacteria	Nocardioides sp. CF8
tr|L7LAD3|L7LAD3_9ACTO	2	Actinobacteria	Actinobacteria	Gordonia hirsuta
tr|W5WSC8|W5WSC8_9PSEU	1	Actinobacteria	Actinobacteria	Kutzneria albida
tr|E6JDS6|E6JDS6_9ACTO	1	Actinobacteria	Actinobacteria	Dietzia cinnamea
tr|G7GSR3|G7GSR3_9ACTO	1	Actinobacteria	Actinobacteria	Gordonia amarae
tr|H5X8G1|H5X8G1_9PSEU	2	Actinobacteria	Actinobacteria	Saccharomonospora marina
tr|X7ZXK3|X7ZXK3_MYCXE	6	Actinobacteria	Actinobacteria	Mycobacterium xenopi
tr|L7LKU3|L7LKU3_9ACTO	1	Actinobacteria	Actinobacteria	Gordonia sihwensis
tr|J4TJB4|J4TJB4_9MYCO	1	Actinobacteria	Actinobacteria	Mycobacterium colombiense

tr|X7YM24|X7YM24_MYCKA	2	Actinobacteria	Actinobacteria	Mycobacterium kansasii
tr|A0A024M5I3|A0A024M5I3_9MYCO	10	Actinobacteria	Actinobacteria	Mycobacterium farcinogenes
tr|H5X5Q8|H5X5Q8_9PSEU	1	Actinobacteria	Actinobacteria	Saccharomonospora marina
tr|E9V0Y9|E9V0Y9_9ACTO	1	Actinobacteria	Actinobacteria	Nocardioidaceae bacterium Broad-1
tr|C3JUL6|C3JUL6_RHOER	1	Actinobacteria	Actinobacteria	Rhodococcus erythropolis
tr|X8CLF3|X8CLF3_MYCIT	12	Actinobacteria	Actinobacteria	Mycobacterium intracellulare
tr|G7GKE4|G7GKE4_9ACTO	1	Actinobacteria	Actinobacteria	Gordonia amarae
tr|G7GUZ7|G7GUZ7_9ACTO	1	Actinobacteria	Actinobacteria	Gordonia amarae
tr|Q5YPJ0|Q5YPJ0_NOCFA	1	Actinobacteria	Actinobacteria	Nocardia farcinica
tr|X8DN68|X8DN68_MYCXE	6	Actinobacteria	Actinobacteria	Mycobacterium xenopi
tr|A0A0A1DL73|A0A0A1DL73_NOCSI	1	Actinobacteria	Actinobacteria	Pimelobacter simplex
tr|A0A0B8NG66|A0A0B8NG66_9NOCA	1	Actinobacteria	Actinobacteria	Nocardia seriolae
tr|C1A0D9|C1A0D9_RHOE4	2	Actinobacteria	Actinobacteria	Rhodococcus erythropolis
tr|A0A098BNV6|A0A098BNV6_9NOCA	1	Actinobacteria	Actinobacteria	Rhodococcus ruber
tr|L8DFS3|L8DFS3_9NOCA	1	Actinobacteria	Actinobacteria	Rhodococcus sp. AW25M09
tr|E2SCQ8|E2SCQ8_9ACTO	2	Actinobacteria	Actinobacteria	Aeromicrobium marinum
tr|D0L6Z6|D0L6Z6_GORB4	1	Actinobacteria	Actinobacteria	Gordonia bronchialis
tr|A0A0A1DP83|A0A0A1DP83_NOCSI	2	Actinobacteria	Actinobacteria	Pimelobacter simplex
tr|A0A049DYL2|A0A049DYL2_MYCAV	1	Actinobacteria	Actinobacteria	Mycobacterium avium
tr|F5YX50|F5YX50_MYCSD	1	Actinobacteria	Actinobacteria	Mycobacterium sp. JDM601
tr|W5TJZ0|W5TJZ0_9NOCA	1	Actinobacteria	Actinobacteria	Nocardia nova
tr|D2B9V9|D2B9V9_STRRD	2	Actinobacteria	Actinobacteria	Streptosporangium roseum
tr|V8CVX7|V8CVX7_9ACTO	6	Actinobacteria	Actinobacteria	Williamsia sp. D3
tr|K8XTI5|K8XTI5_RHOOP	1	Actinobacteria	Actinobacteria	Rhodococcus opacus
tr|D1A3I7|D1A3I7_THECD	2	Actinobacteria	Actinobacteria	Thermomonospora curvata
tr|H0R663|H0R663_9ACTO	1	Actinobacteria	Actinobacteria	Gordonia effusa
tr|M2VYX2|M2VYX2_9NOCA	1	Actinobacteria	Actinobacteria	Rhodococcus triatomae

tr|F6EN10|F6EN10_AMYSD	1	Actinobacteria	Actinobacteria	Amycolicicoccus subflavus
tr|F6EHS4|F6EHS4_AMYSD	1	Actinobacteria	Actinobacteria	Amycolicicoccus subflavus
tr|D1AD47|D1AD47_THECD	2	Actinobacteria	Actinobacteria	Thermomonospora curvata
tr|E9UWP3|E9UWP3_9ACTO	1	Actinobacteria	Actinobacteria	Nocardioidaceae bacterium Broad-1
tr|H5X8G0|H5X8G0_9PSEU	1	Actinobacteria	Actinobacteria	Saccharomonospora marina
tr|R4MBA2|R4MBA2_MYCTX	1	Actinobacteria	Actinobacteria	Mycobacterium tuberculosis
tr|G7GP50|G7GP50_9ACTO	1	Actinobacteria	Actinobacteria	Gordonia amarae
tr|A0A034UEJ5|A0A034UEJ5_9NOCA	1	Actinobacteria	Actinobacteria	Nocardia brasiliensis
tr|H0R4X4|H0R4X4_9ACTO	1	Actinobacteria	Actinobacteria	Gordonia effusa
tr|H0QWF9|H0QWF9_9ACTO	1	Actinobacteria	Actinobacteria	Gordonia effusa
tr|L8DEL3|L8DEL3_9NOCA	1	Actinobacteria	Actinobacteria	Rhodococcus sp. AW25M09
tr|G4HR53|G4HR53_MYCRH	1	Actinobacteria	Actinobacteria	Mycobacterium rhodesiae
tr|H5UKZ3|H5UKZ3_9ACTO	1	Actinobacteria	Actinobacteria	Gordonia terrae
tr|H0QWP1|H0QWP1_9ACTO	1	Actinobacteria	Actinobacteria	Gordonia effusa
tr|G7H7G1|G7H7G1_9ACTO	1	Actinobacteria	Actinobacteria	Gordonia araii
tr|A0A0C1LBJ1|A0A0C1LBJ1_9PSEU	2	Actinobacteria	Actinobacteria	Prauserella sp. Am3
tr|G7GTT1|G7GTT1_9ACTO	1	Actinobacteria	Actinobacteria	Gordonia amarae
tr|A0A0A1FTG8|A0A0A1FTG8_9MYCO	1	Actinobacteria	Actinobacteria	Mycobacterium sp. VKM Ac-1817D
tr|J9SEN1|J9SEN1_9ACTO	1	Actinobacteria	Actinobacteria	Gordonia sp. KTR9
tr|A0A069JAN8|A0A069JAN8_9NOCA	2	Actinobacteria	Actinobacteria	Rhodococcus qingshengii
tr|D1A3A1|D1A3A1_THECD	1	Actinobacteria	Actinobacteria	Thermomonospora curvata
tr|L7LAG4|L7LAG4_9ACTO	1	Actinobacteria	Actinobacteria	Gordonia hirsuta
tr|H5UG93|H5UG93_9ACTO	1	Actinobacteria	Actinobacteria	Gordonia terrae
tr|R4UZM2|R4UZM2_MYCAB	1	Actinobacteria	Actinobacteria	Mycobacterium abscessus
tr|Q82B07|Q82B07_STRAW	1	Actinobacteria	Actinobacteria	Streptomyces avermitilis
tr|J9RFH6|J9RFH6_9ACTO	1	Actinobacteria	Actinobacteria	Gordonia sp. KTR9
tr|H6RCR8|H6RCR8_NOCCG	1	Actinobacteria	Actinobacteria	Nocardia cyriacigeorgica

tr|M2VAL0|M2VAL0_9NOCA	1	Actinobacteria	Actinobacteria	Rhodococcus triatomae
tr|H6RMV0|H6RMV0_BLASD	1	Actinobacteria	Actinobacteria	Blastococcus saxobsidens
tr|R1IB31|R1IB31_9PSEU	1	Actinobacteria	Actinobacteria	Amycolatopsis vancoresmycina
tr|G7GXB3|G7GXB3_9ACTO	1	Actinobacteria	Actinobacteria	Gordonia araii
tr|E2SGB1|E2SGB1_9ACTO	1	Actinobacteria	Actinobacteria	Aeromicrobium marinum
tr|G7GSQ0|G7GSQ0_9ACTO	1	Actinobacteria	Actinobacteria	Gordonia amarae
tr|G7GKE2|G7GKE2_9ACTO	1	Actinobacteria	Actinobacteria	Gordonia amarae
tr|D2B9V8|D2B9V8_STRRD	1	Actinobacteria	Actinobacteria	Streptosporangium roseum
tr|A0A0B8NBL1|A0A0B8NBL1_9NOCA	1	Actinobacteria	Actinobacteria	Nocardia seriolae
tr|E5XTR1|E5XTR1_9ACTO	6	Actinobacteria	Actinobacteria	Segniliparus rugosus
tr|R7XWT8|R7XWT8_9ACTO	24	Actinobacteria	Actinobacteria	Nocardioides sp. CF8
tr|F4CXJ2|F4CXJ2_PSEUX	1	Actinobacteria	Actinobacteria	Pseudonocardia dioxanivorans
tr|Q5YVH1|Q5YVH1_NOCFA	1	Actinobacteria	Actinobacteria	Nocardia farcinica
tr|D5UP35|D5UP35_TSUPD	1	Actinobacteria	Actinobacteria	Tsukamurella paurometabola
tr|U1ZJL3|U1ZJL3_9BURK	1	Proteobacteria	Betaproteobacteria	Alcaligenes sp. EGD-AK7
tr|A0A031MIE7|A0A031MIE7_9PSED	1	Proteobacteria	Gammaproteobacteria	Pseudomonas bauzanensis
tr|J6I7B6|J6I7B6_9ENTR	1	Proteobacteria	Gammaproteobacteria	Klebsiella sp. OBRC7
tr|N6YC44|N6YC44_9RHOO	1	Proteobacteria	Betaproteobacteria	Thauera sp. 63
tr|Q2Y8L0|Q2Y8L0_NITMU	1	Proteobacteria	Betaproteobacteria	Nitrosospira multiformis
tr|A0A0B2DAV4|A0A0B2DAV4_9GAMM	1	Proteobacteria	Gammaproteobacteria	Serpens flexibilis
tr|H2FYH2|H2FYH2_OCESG	1	Proteobacteria	Gammaproteobacteria	Oceanimonas sp. GK1
tr|H8L0A6|H8L0A6_FRAAD	1	Proteobacteria	Gammaproteobacteria	Frateuria aurantia
tr|C4LEE8|C4LEE8_TOLAT	1	Proteobacteria	Gammaproteobacteria	Tolumonas auensis
tr|W0PAK2|W0PAK2_9BURK	1	Proteobacteria	Betaproteobacteria	Advenella mimigardefordensis
tr|T1CD74|T1CD74_9ZZZZ	1			mine drainage metagenome
tr|T2LCY9|T2LCY9_9GAMM	1	Proteobacteria	Gammaproteobacteria	Halomonas sp. A3H3
tr|A0A072N462|A0A072N462_9ALTE	1	Proteobacteria	Gammaproteobacteria	Marinobacter sp. AK21

tr|G5Q9L4|G5Q9L4_SALMO	1	Proteobacteria	Gammaproteobacteria	Salmonella enterica
tr|U7P997|U7P997_9GAMM	1	Proteobacteria	Gammaproteobacteria	Halomonas sp. PBN3
tr|A0A0A0M9P8|A0A0A0M9P8_9GAMM	1	Proteobacteria	Gammaproteobacteria	Lysobacter defluvii
tr|I2B9J8|I2B9J8_SHIBC	1	Proteobacteria	Gammaproteobacteria	Shimwellia blattae
tr|A0A077DC69|A0A077DC69_9BURK	1	Proteobacteria	Betaproteobacteria	Basilea psittacipulmonis
tr|A1WY43|A1WY43_HALHL	1	Proteobacteria	Gammaproteobacteria	Halorhodospira halophila
tr|V8G7S9|V8G7S9_9BURK	1	Proteobacteria	Betaproteobacteria	Pelistega sp. HM-7
tr|A0A096BDQ0|A0A096BDQ0_9BURK	1	Proteobacteria	Betaproteobacteria	Oligella urethralis
tr|X5M6E7|X5M6E7_9PROT	1	Proteobacteria	Alphaproteobacteria	Candidatus Phaeomarinobacter ectocarpi
tr|A7HW40|A7HW40_PARL1	1	Proteobacteria	Alphaproteobacteria	Parvibaculum lavamentivorans
tr|A0A081B9S0|A0A081B9S0_9PROT	1	Proteobacteria	Alphaproteobacteria	alpha proteobacterium MA2
tr|W9H4G6|W9H4G6_9PROT	1	Proteobacteria	Alphaproteobacteria	Skermanella stibiiresistens
tr|M2TL71|M2TL71_9PROT	1	Proteobacteria	Alphaproteobacteria	alpha proteobacterium JLT2015
tr|A0A0C1U256|A0A0C1U256_9CYAN	1	Cyanobacteria		Scytonema millei
tr|A0A069E8D6|A0A069E8D6_9RHOB	1	Proteobacteria	Alphaproteobacteria	Hyphomonas adhaerens
tr|B6IT87|B6IT87_RHOCS	1	Proteobacteria	Alphaproteobacteria	Rhodospirillum centenum
tr|G8APJ5|G8APJ5_AZOBR	1	Proteobacteria	Alphaproteobacteria	Azospirillum brasilense
tr|K2DV61|K2DV61_9BACT	1			uncultured bacterium
tr|D0CZC8|D0CZC8_9RHOB	1	Proteobacteria	Alphaproteobacteria	Citreicella sp. SE45
tr|S7U5S8|S7U5S8_9DELT	1	Proteobacteria	Deltaproteobacteria	Desulfovibrio sp. X2
tr|B8DLS5|B8DLS5_DESVM	1	Proteobacteria	Deltaproteobacteria	Desulfovibrio vulgaris
tr|A0A0B8RE09|A0A0B8RE09_9PROT	1	Proteobacteria	Alphaproteobacteria	alpha proteobacterium U9-1i
tr|A0A061QHA4|A0A061QHA4_9PROT	1	Proteobacteria	Alphaproteobacteria	alpha proteobacterium Q-1
tr|A0A0A1PX21|A0A0A1PX21_9BACT	1			bacterium YEK0313
tr|E3I3Y1|E3I3Y1_RHOVT	1	Proteobacteria	Alphaproteobacteria	Rhodomicrobium vannielii
tr|B4RE08|B4RE08_PHEZH	1	Proteobacteria	Alphaproteobacteria	Phenylobacterium zucineum
tr|J7Q4T2|J7Q4T2_METSZ	1	Proteobacteria	Alphaproteobacteria	Methylocystis sp. SC2

tr|T0GJ97|T0GJ97_9SPHN	1	Proteobacteria	Alphaproteobacteria	Sphingobium baderi
tr|I2K9W2|I2K9W2_9PROT	1	Proteobacteria	Epsilonproteobacteria	Sulfurovum sp. AR
tr|Q3J2I1|Q3J2I1_RHOS4	1	Proteobacteria	Alphaproteobacteria	Rhodobacter sphaeroides
tr|D9QGF1|D9QGF1_BRESC	1	Proteobacteria	Alphaproteobacteria	Brevundimonas subvibrioides
tr|A0A0C1IN25|A0A0C1IN25_9RHOB	1	Proteobacteria	Alphaproteobacteria	Ruegeria sp. ANG-R
tr|A0A074MC36|A0A074MC36_ERYLO	1	Proteobacteria	Alphaproteobacteria	Erythrobacter longus
tr|S6B3W9|S6B3W9_9PROT	1	Proteobacteria	Betaproteobacteria	Sulfuricella denitrificans
tr|A0A0A8K3K2|A0A0A8K3K2_9RHIZ	1	Proteobacteria	Alphaproteobacteria	Methyloceanibacter caenitepidi
tr|K7T2K7|K7T2K7_9HELI	1	Proteobacteria	Epsilonproteobacteria	Candidatus Sulfuricurvum sp. RIFRC-1
tr|T0JP38|T0JP38_9HELI	1	Proteobacteria	Epsilonproteobacteria	Sulfurimonas sp. AST-10
tr|M2SE07|M2SE07_9PROT	1	Proteobacteria	Alphaproteobacteria	alpha proteobacterium JLT2015
tr|E0TB69|E0TB69_PARBH	1	Proteobacteria	Alphaproteobacteria	Parvularcula bermudensis
tr|C6XN24|C6XN24_HIRBI	1	Proteobacteria	Alphaproteobacteria	Hirschia baltica
tr|B1Z830|B1Z830_METPB	1	Proteobacteria	Alphaproteobacteria	Methylobacterium populi
tr|A0A0A0D684|A0A0A0D684_9PROT	1	Proteobacteria	Alphaproteobacteria	Inquilinus limosus
tr|I4YW72|I4YW72_9RHIZ	1	Proteobacteria	Alphaproteobacteria	Microvirga lotononidis
tr|I3TKF0|I3TKF0_TISMK	1	Proteobacteria	Alphaproteobacteria	Tistrella mobilis
tr|A0A095CYU7|A0A095CYU7_9SPHN	1	Proteobacteria	Alphaproteobacteria	Sphingopyxis sp. LC363
tr|A0A087M978|A0A087M978_9PROT	1	Proteobacteria	Epsilonproteobacteria	Sulfurospirillum sp. SCADC
tr|W0JMI0|W0JMI0_DESAE	1	Proteobacteria	Deltaproteobacteria	Desulfurella acetivorans
tr|B8ET81|B8ET81_METSB	1	Proteobacteria	Alphaproteobacteria	Methylocella silvestris
tr|A0A085EY42|A0A085EY42_9BRAD	1	Proteobacteria	Alphaproteobacteria	Bosea sp. LC85
tr|B6R083|B6R083_9RHOB	1	Proteobacteria	Alphaproteobacteria	Pseudovibrio sp. JE062
tr|D5BVM5|D5BVM5_NITHN	1	Proteobacteria	Gammaproteobacteria	Nitrosococcus halophilus
tr|V4JE12|V4JE12_9DELT	1	Proteobacteria	Deltaproteobacteria	uncultured Desulfofustis sp. PB-SRB1
tr|G2HRZ6|G2HRZ6_9PROT	1	Proteobacteria	Epsilonproteobacteria	Arcobacter sp. L
tr|E8RQ92|E8RQ92_ASTEC	1	Proteobacteria	Alphaproteobacteria	Asticcacaulis excentricus

tr|U4V650|U4V650_9RHIZ	1	Proteobacteria	Alphaproteobacteria	Ochrobactrum intermedium
tr|U7G9H6|U7G9H6_9RHOB	1	Proteobacteria	Alphaproteobacteria	Labrenzia sp. C1B10
tr|A0A086YEL8|A0A086YEL8_9RHOB	1	Proteobacteria	Alphaproteobacteria	Haematobacter missouriensis
tr|W6LSR3|W6LSR3_9GAMM	1	Proteobacteria	Gammaproteobacteria	Candidatus Contendobacter odensis
tr|D7A0D4|D7A0D4_STAND	1	Proteobacteria	Alphaproteobacteria	Starkeya novella
tr|A0A085FIL3|A0A085FIL3_9RHIZ	1	Proteobacteria	Alphaproteobacteria	Devosia sp. LC5
tr|J9A1J9|J9A1J9_9RHOB	1	Proteobacteria	Alphaproteobacteria	Rhodovulum sp. PH10
tr|A9DQS1|A9DQS1_9RHOB	1	Proteobacteria	Alphaproteobacteria	Oceanibulbus indolifex
tr|I1AR71|I1AR71_9RHOB	1	Proteobacteria	Alphaproteobacteria	Citreicella sp. 357
tr|A8TM92|A8TM92_9PROT	1	Proteobacteria	Alphaproteobacteria	alpha proteobacterium BAL199
tr|E6PQ34|E6PQ34_9ZZZZ	1			mine drainage metagenome
tr|F8JCY2|F8JCY2_HYPSM	7	Proteobacteria	Alphaproteobacteria	Hyphomicrobium sp. MC1
tr|D8PJK4|D8PJK4_9BACT	1	Nitrospirae	Nitrospira	Candidatus Nitrospira defluvii
tr|A0A077AV80|A0A077AV80_9RICK	1	Proteobacteria	Alphaproteobacteria	Candidatus Paracaedibacter acanthamoebae
tr|V4RDJ9|V4RDJ9_9RHIZ	1	Proteobacteria	Alphaproteobacteria	Lutibaculum baratangense
tr|A0A0C2W1Z7|A0A0C2W1Z7_9PROT	1	Proteobacteria	Epsilonproteobacteria	Sulfurovum sp. FS08-3
tr|C9Y6W8|C9Y6W8_9BURK	1	Proteobacteria	Betaproteobacteria	putative symbiont of Hydra magnipapillata
tr|A0A0C2VMD8|A0A0C2VMD8_9PROT	1	Proteobacteria	Epsilonproteobacteria	Sulfurovum sp. FS06-10
tr|E6WYF4|E6WYF4_NITSE	1	Proteobacteria	Epsilonproteobacteria	Nitratifractor salsuginis
tr|D7DJ74|D7DJ74_METV0	1	Proteobacteria	Betaproteobacteria	Methylotenera versatilis
tr|A0A011QPF4|A0A011QPF4_9PROT	1	Proteobacteria	Betaproteobacteria	Candidatus Accumulibacter sp. BA-93
tr|F2J3B6|F2J3B6_POLGS	1	Proteobacteria	Alphaproteobacteria	Polymorphum gilvum
tr|I3BYE7|I3BYE7_9GAMM	1	Proteobacteria	Gammaproteobacteria	Thiothrix nivea
tr|A9NDG9|A9NDG9_COXBR	1	Proteobacteria	Gammaproteobacteria	Coxiella burnetii
tr|A8I863|A8I863_AZOC5	1	Proteobacteria	Alphaproteobacteria	Azorhizobium caulinodans
tr|A0A017HET3|A0A017HET3_9RHOB	1	Proteobacteria	Alphaproteobacteria	Loktanella hongkongensis
tr|Q47GR7|Q47GR7_DECAR	1	Proteobacteria	Betaproteobacteria	Dechloromonas aromatica

tr|Q13AL4|Q13AL4_RHOPS	1	Proteobacteria	Alphaproteobacteria	Rhodopseudomonas palustris
tr|K2BGD7|K2BGD7_9BACT	1			uncultured bacterium
tr|K9GQ74|K9GQ74_9PROT	1	Proteobacteria	Alphaproteobacteria	Caenispirillum salinarum
tr|D0L0H1|D0L0H1_HALNC	1	Proteobacteria	Gammaproteobacteria	Halothiobacillus neapolitanus
tr|S9RL13|S9RL13_9RHOB	1	Proteobacteria	Alphaproteobacteria	Salipiger mucosus
tr|A0A099FAI4|A0A099FAI4_PARVE	1	Proteobacteria	Alphaproteobacteria	Paracoccus versutus
tr|A0A094MYJ3|A0A094MYJ3_9GAMM	1	Proteobacteria	Gammaproteobacteria	Thiobacillus prosperus
tr|N0B1J2|N0B1J2_9RHIZ	7	Proteobacteria	Alphaproteobacteria	Hyphomicrobium denitrificans
tr|A0A0A8K6N0|A0A0A8K6N0_9RHIZ	7	Proteobacteria	Alphaproteobacteria	Methyloceanibacter caenitepidi
tr|Q11I37|Q11I37_CHESB	1	Proteobacteria	Alphaproteobacteria	Chelativorans sp. BNC1
tr|W0BHR6|W0BHR6_9GAMM	1	Proteobacteria	Gammaproteobacteria	Legionella oakridgensis
tr|A5IDN0|A5IDN0_LEGPC	1	Proteobacteria	Gammaproteobacteria	Legionella pneumophila
tr|W0TJP3|W0TJP3_9GAMM	1	Proteobacteria	Gammaproteobacteria	Thiolapillus brandeum
tr|X4Z9H6|X4Z9H6_9BORD	7	Proteobacteria	Betaproteobacteria	Bordetella holmesii
tr|X7ELT6|X7ELT6_9RHOB	1	Proteobacteria	Alphaproteobacteria	Roseivivax halodurans
tr|A0A090BUD1|A0A090BUD1_9GAMM	1	Proteobacteria	Gammaproteobacteria	Thioploca ingrica
tr|K2BNS1|K2BNS1_9BACT	1			uncultured bacterium
tr|H5SAC6|H5SAC6_9GAMM	1	Proteobacteria	Gammaproteobacteria	uncultured gamma proteobacterium
tr|H0PSW3|H0PSW3_9RHOO	1	Proteobacteria	Betaproteobacteria	Azoarcus sp. KH32C
tr|A4BL42|A4BL42_9GAMM	1	Proteobacteria	Gammaproteobacteria	Nitrococcus mobilis
tr|V5SGA2|V5SGA2_9RHIZ	7	Proteobacteria	Alphaproteobacteria	Hyphomicrobium nitrativorans
tr|Q3SM48|Q3SM48_THIDA	1	Proteobacteria	Betaproteobacteria	Thiobacillus denitrificans
tr|W0V0P9|W0V0P9_9BURK	1	Proteobacteria	Betaproteobacteria	Janthinobacterium agaricidamnosum
tr|A8PLY9|A8PLY9_9COXI	1	Proteobacteria	Gammaproteobacteria	Rickettsiella grylli
tr|G4RGN1|G4RGN1_PELHB	1	Proteobacteria	Alphaproteobacteria	Pelagibacterium halotolerans
tr|U2Z7D0|U2Z7D0_9RHOB	1	Proteobacteria	Alphaproteobacteria	Loktanella cinnabarina
tr|M9RIN2|M9RIN2_9RHOB	1	Proteobacteria	Alphaproteobacteria	Octadecabacter arcticus

tr|A0A0A1FJ80|A0A0A1FJ80_9BURK	1	Proteobacteria	Betaproteobacteria	Collimonas arenae
tr|A0A093TGR2|A0A093TGR2_RALSL	1	Proteobacteria	Betaproteobacteria	Ralstonia solanacearum
tr|A0A099EVU0|A0A099EVU0_9RHOB	1	Proteobacteria	Alphaproteobacteria	Paracoccus halophilus
tr|B2IFF5|B2IFF5_BEII9	7	Proteobacteria	Alphaproteobacteria	Beijerinckia indica
tr|Q0A9D5|Q0A9D5_ALKEH	1	Proteobacteria	Gammaproteobacteria	Alkalilimnicola ehrlichii
tr|A0A0A8GQ79|A0A0A8GQ79_9PROT	1	Proteobacteria	Epsilonproteobacteria	Campylobacter peloridis
tr|A0A084INE6|A0A084INE6_9GAMM	1	Proteobacteria	Gammaproteobacteria	Salinisphaera hydrothermalis
tr|M3IM68|M3IM68_9PROT	1	Proteobacteria	Epsilonproteobacteria	Campylobacter showae
tr|L8XXV6|L8XXV6_9GAMM	1	Proteobacteria	Gammaproteobacteria	Wohlfahrtiimonas chitiniclastica
tr|T1X3K2|T1X3K2_VARPD	1	Proteobacteria	Betaproteobacteria	Variovorax paradoxus
tr|F5Y4Q4|F5Y4Q4_RAMTT	1	Proteobacteria	Betaproteobacteria	Ramlibacter tataouinensis
tr|K2BWT1|K2BWT1_9BACT	1			uncultured bacterium
tr|M5DVB0|M5DVB0_9PROT	1	Proteobacteria	Betaproteobacteria	Nitrosospira sp. APG3
tr|B4UAD8|B4UAD8_ANASK	29	Proteobacteria	Deltaproteobacteria	Anaeromyxobacter sp. K
tr|Q0G008|Q0G008_9RHIZ	1	Proteobacteria	Alphaproteobacteria	Fulvimarina pelagi
tr|S3XRK7|S3XRK7_9PROT	1	Proteobacteria	Epsilonproteobacteria	Campylobacter ureolyticus
tr|A0A059HYU4|A0A059HYU4_CAMJU	1	Proteobacteria	Epsilonproteobacteria	Campylobacter jejuni
tr|O67491|O67491_AQUAE	1	Aquificae	Aquificae	Aquifex aeolicus
tr|A1SVF6|A1SVF6_PSYIN	1	Proteobacteria	Gammaproteobacteria	Psychromonas ingrahamii
tr|N6V7S3|N6V7S3_9RHIZ	1	Proteobacteria	Alphaproteobacteria	Rhizobium freirei
tr|F5R923|F5R923_METUF	1	Proteobacteria	Betaproteobacteria	Methyloversatilis universalis
tr|Q1YMZ5|Q1YMZ5_MOBAS	1	Proteobacteria	Alphaproteobacteria	Aurantimonas manganoxydans
tr|E0MKS7|E0MKS7_9RHOB	1	Proteobacteria	Alphaproteobacteria	Ahrensia sp. R2A130
tr|L0EV30|L0EV30_LIBCB	36	Proteobacteria	Alphaproteobacteria	Liberibacter crescens
tr|Q1GYQ2|Q1GYQ2_METFK	1	Proteobacteria	Betaproteobacteria	Methylobacillus flagellatus
tr|A0A0A8BEL8|A0A0A8BEL8_9BURK	1	Proteobacteria	Betaproteobacteria	Pandoraea sputorum
tr|C9RK73|C9RK73_FIBSS	1	Fibrobacteres	Fibrobacteria	Fibrobacter succinogenes

tr|U1H7F6|U1H7F6_9BRAD	1	Proteobacteria	Alphaproteobacteria	Bradyrhizobium sp. DFCI-1
tr|E7G4E4|E7G4E4_9HELI	1	Proteobacteria	Epsilonproteobacteria	Helicobacter suis
tr|G3IS04|G3IS04_9GAMM	1	Proteobacteria	Gammaproteobacteria	Methylobacter tundripaludum
tr|E6VPM6|E6VPM6_RHOPX	7	Proteobacteria	Alphaproteobacteria	Rhodopseudomonas palustris
tr|U6B5C2|U6B5C2_9RHIZ	1	Proteobacteria	Alphaproteobacteria	Candidatus Liberibacter americanus
tr|H9UGG4|H9UGG4_SPIAZ	1	Spirochaetes	Spirochaetia	Spirochaeta africana
tr|C8PG52|C8PG52_9PROT	1	Proteobacteria	Epsilonproteobacteria	Campylobacter gracilis
tr|E5AMH6|E5AMH6_BURRH	1	Proteobacteria	Betaproteobacteria	Burkholderia rhizoxinica
tr|Q82TE2|Q82TE2_NITEU	1	Proteobacteria	Betaproteobacteria	Nitrosomonas europaea
tr|T1DHA0|T1DHA0_9ZZZZ	1			mine drainage metagenome
tr|A7ZD92|A7ZD92_CAMC1	1	Proteobacteria	Epsilonproteobacteria	Campylobacter concisus
tr|A0A063Y3A9|A0A063Y3A9_9GAMM	1	Proteobacteria	Gammaproteobacteria	Nitrincola lacisaponensis
tr|B0VHY9|B0VHY9_CLOAI	1	Cloacimonetes		Candidatus Cloacimonas acidaminovorans
tr|F9ZMQ1|F9ZMQ1_ACICS	1	Proteobacteria	Gammaproteobacteria	Acidithiobacillus caldus
tr|M1Q0H5|M1Q0H5_9ZZZZ	1			uncultured organism
tr|E4QMA4|E4QMA4_METS6	1	Proteobacteria	Betaproteobacteria	Methylovorus sp. MP688
tr|X0SVT9|X0SVT9_9ZZZZ	1			marine sediment metagenome
tr|R9Q3A0|R9Q3A0_9AQUI	1	Aquificae	Aquificae	Hydrogenobaculum sp. SHO
tr|A0A0A1VM91|A0A0A1VM91_9BURK	1	Proteobacteria	Betaproteobacteria	Acidovorax sp. MR-S7
tr|H1XWC3|H1XWC3_9BACT	1			Caldithrix abyssi
tr|U5XWV8|U5XWV8_CAMFE	1	Proteobacteria	Epsilonproteobacteria	Campylobacter fetus
tr|A0A038GNJ3|A0A038GNJ3_9BURK	1	Proteobacteria	Betaproteobacteria	Burkholderia sp. MP-1
tr|Q7MQT8|Q7MQT8_WOLSU	1	Proteobacteria	Epsilonproteobacteria	Wolinella succinogenes
tr|D6Z3S5|D6Z3S5_DESAT	1	Proteobacteria	Deltaproteobacteria	Desulfurivibrio alkaliphilus
tr|Q60CD6|Q60CD6_METCA	1	Proteobacteria	Gammaproteobacteria	Methylococcus capsulatus
tr|E6QSE7|E6QSE7_9ZZZZ	1			mine drainage metagenome
tr|U7DAR1|U7DAR1_9BACT	1	Fibrobacteres	Chitinivibrionia	Chitinivibrio alkaliphilus

tr|E8WAI5|E8WAI5_STRFA	2	Actinobacteria	Actinobacteria	Streptomyces pratensis
tr|W5WGQ0|W5WGQ0_9PSEU	2	Actinobacteria	Actinobacteria	Kutzneria albida
tr|H5XNG6|H5XNG6_9PSEU	2	Actinobacteria	Actinobacteria	Saccharomonospora cyanea
tr|F4CXJ7|F4CXJ7_PSEUX	2	Actinobacteria	Actinobacteria	Pseudonocardia dioxanivorans
tr|A0A099D9G8|A0A099D9G8_9ACTO	2	Actinobacteria	Actinobacteria	Actinopolyspora erythraea
tr|D9UWI5|D9UWI5_9ACTO	2	Actinobacteria	Actinobacteria	Streptomyces sp. AA4
tr|M2Z3P3|M2Z3P3_9PSEU	2	Actinobacteria	Actinobacteria	Amycolatopsis decaplanina
tr|A0A0A1DIE7|A0A0A1DIE7_NOCSI	2	Actinobacteria	Actinobacteria	Pimelobacter simplex
tr|A0A0B7CGE0|A0A0B7CGE0_9PSEU	2	Actinobacteria	Actinobacteria	Kibdelosporangium sp. MJ126-NF4
tr|A0A0C1L1Y3|A0A0C1L1Y3_9PSEU	2	Actinobacteria	Actinobacteria	Prauserella sp. Am3
tr|C6WH34|C6WH34_ACTMD	2	Actinobacteria	Actinobacteria	Actinosynnema mirum
tr|D2PUA3|D2PUA3_KRIFD	2	Actinobacteria	Actinobacteria	Kribbella flavida
tr|W9DM11|W9DM11_9PSEU	2	Actinobacteria	Actinobacteria	Amycolatopsis halophila
tr|A0A081EI46|A0A081EI46_STRFR	2	Actinobacteria	Actinobacteria	Streptomyces fradiae
tr|V9XFQ6|V9XFQ6_9NOCA	2	Actinobacteria	Actinobacteria	Rhodococcus pyridinivorans
tr|D3Q8W0|D3Q8W0_STANL	2	Actinobacteria	Actinobacteria	Stackebrandtia nassauensis
tr|J9SC86|J9SC86_9ACTO	2	Actinobacteria	Actinobacteria	Gordonia sp. KTR9
tr|H6RMV1|H6RMV1_BLASD	2	Actinobacteria	Actinobacteria	Blastococcus saxobsidens
tr|R7Y1D9|R7Y1D9_9ACTO	2	Actinobacteria	Actinobacteria	Nocardioides sp. CF8
tr|E6S9N9|E6S9N9_INTC7	2	Actinobacteria	Actinobacteria	Intrasporangium calvum
tr|E6JBH9|E6JBH9_9ACTO	2	Actinobacteria	Actinobacteria	Dietzia cinnamea
tr|E2SGB3|E2SGB3_9ACTO	2	Actinobacteria	Actinobacteria	Aeromicrobium marinum
tr|A0A0A1DTM0|A0A0A1DTM0_NOCSI	2	Actinobacteria	Actinobacteria	Pimelobacter simplex
tr|D1A3A3|D1A3A3_THECD	2	Actinobacteria	Actinobacteria	Thermomonospora curvata
tr|F6EHT1|F6EHT1_AMYSD	2	Actinobacteria	Actinobacteria	Amycolicicoccus subflavus
tr|D5UQ87|D5UQ87_TSUPD	2	Actinobacteria	Actinobacteria	Tsukamurella paurometabola
tr|E9UWC8|E9UWC8_9ACTO	2	Actinobacteria	Actinobacteria	Nocardioidaceae bacterium Broad-1

tr|T5IB76|T5IB76_RHOER	2	Actinobacteria	Actinobacteria	Rhodococcus erythropolis
tr|A0A0B2BME5|A0A0B2BME5_9ACTO	2	Actinobacteria	Actinobacteria	Mumia flava
tr|A0A0B8N9A0|A0A0B8N9A0_9NOCA	2	Actinobacteria	Actinobacteria	Nocardia seriolae
tr|Q5YWK4|Q5YWK4_NOCFA	2	Actinobacteria	Actinobacteria	Nocardia farcinica
tr|W5TFE2|W5TFE2_9NOCA	2	Actinobacteria	Actinobacteria	Nocardia nova
tr|A0A022LN77|A0A022LN77_9ACTO	2	Actinobacteria	Actinobacteria	Dietzia sp. UCD-THP
tr|R7XXX0|R7XXX0_9ACTO	2	Actinobacteria	Actinobacteria	Nocardioides sp. CF8
tr|F6EN33|F6EN33_AMYSD	2	Actinobacteria	Actinobacteria	Amycolicicoccus subflavus
tr|G7GUZ4|G7GUZ4_9ACTO	2	Actinobacteria	Actinobacteria	Gordonia amarae
tr|E9V0Z2|E9V0Z2_9ACTO	2	Actinobacteria	Actinobacteria	Nocardioidaceae bacterium Broad-1
tr|H5X8F8|H5X8F8_9PSEU	2	Actinobacteria	Actinobacteria	Saccharomonospora marina
tr|W8HDM9|W8HDM9_RHOOP	2	Actinobacteria	Actinobacteria	Rhodococcus opacus
tr|W5TAK2|W5TAK2_9NOCA	2	Actinobacteria	Actinobacteria	Nocardia nova
tr|A0A076N1G7|A0A076N1G7_AMYME	2	Actinobacteria	Actinobacteria	Amycolatopsis methanolica
tr|M0QIR0|M0QIR0_9ACTO	2	Actinobacteria	Actinobacteria	Gordonia soli
tr|A0A0B2Y2J3|A0A0B2Y2J3_9MYCO	2	Actinobacteria	Actinobacteria	Mycobacterium setense
tr|A1TCM7|A1TCM7_MYCVP	2	Actinobacteria	Actinobacteria	Mycobacterium vanbaalenii
tr|W5WJM9|W5WJM9_9PSEU	2	Actinobacteria	Actinobacteria	Kutzneria albida
tr|L7KN85|L7KN85_9ACTO	2	Actinobacteria	Actinobacteria	Gordonia aichiensis
tr|L7L6G7|L7L6G7_9ACTO	2	Actinobacteria	Actinobacteria	Gordonia hirsuta
tr|L7LN06|L7LN06_9ACTO	2	Actinobacteria	Actinobacteria	Gordonia sihwensis
tr|X8A4N1|X8A4N1_MYCAV	2	Actinobacteria	Actinobacteria	Mycobacterium avium
tr|L7KHC4|L7KHC4_9ACTO	2	Actinobacteria	Actinobacteria	Gordonia aichiensis
tr|X7YA39|X7YA39_MYCKA	2	Actinobacteria	Actinobacteria	Mycobacterium kansasii
tr|R4MIZ7|R4MIZ7_MYCTX	2	Actinobacteria	Actinobacteria	Mycobacterium tuberculosis
tr|X0QWU6|X0QWU6_9NOCA	2	Actinobacteria	Actinobacteria	Rhodococcus wratislaviensis
tr|G8RXD4|G8RXD4_MYCRN	2	Actinobacteria	Actinobacteria	Mycobacterium rhodesiae

tr|Q8VKQ6|Q8VKQ6_MYCTO	2	Actinobacteria	Actinobacteria	Mycobacterium tuberculosis
tr|H5TQ33|H5TQ33_9ACTO	2	Actinobacteria	Actinobacteria	Gordonia otitidis
tr|L0INV5|L0INV5_MYCSM	2	Actinobacteria	Actinobacteria	Mycobacterium smegmatis
tr|L8DA64|L8DA64_9NOCA	2	Actinobacteria	Actinobacteria	Rhodococcus sp. AW25M09
tr|G7GSR6|G7GSR6_9ACTO	2	Actinobacteria	Actinobacteria	Gordonia amarae
tr|K5BIM6|K5BIM6_9MYCO	2	Actinobacteria	Actinobacteria	Mycobacterium hassiacum
tr|C1B001|C1B001_RHOOB	2	Actinobacteria	Actinobacteria	Rhodococcus opacus
tr|M2XHC6|M2XHC6_9NOCA	2	Actinobacteria	Actinobacteria	Rhodococcus triatomae
tr|H5X5Q5|H5X5Q5_9PSEU	2	Actinobacteria	Actinobacteria	Saccharomonospora marina
tr|H6RAR5|H6RAR5_NOCCG	2	Actinobacteria	Actinobacteria	Nocardia cyriacigeorgica
tr|A0A0A1DSN4|A0A0A1DSN4_NOCSI	2	Actinobacteria	Actinobacteria	Pimelobacter simplex
tr|M0QMW8|M0QMW8_9ACTO	2	Actinobacteria	Actinobacteria	Gordonia soli
tr|K0V4J6|K0V4J6_MYCFO	2	Actinobacteria	Actinobacteria	Mycobacterium fortuitum
tr|E2S871|E2S871_9ACTO	2	Actinobacteria	Actinobacteria	Aeromicrobium marinum
tr|G7GP47|G7GP47_9ACTO	2	Actinobacteria	Actinobacteria	Gordonia amarae
tr|H0R660|H0R660_9ACTO	2	Actinobacteria	Actinobacteria	Gordonia effusa
tr|X7Y291|X7Y291_MYCKA	2	Actinobacteria	Actinobacteria	Mycobacterium kansasii
tr|U0EPN6|U0EPN6_9NOCA	2	Actinobacteria	Actinobacteria	Rhodococcus sp. P27
tr|M2WUF8|M2WUF8_9NOCA	2	Actinobacteria	Actinobacteria	Rhodococcus triatomae
tr|I0PGT7|I0PGT7_MYCAB	2	Actinobacteria	Actinobacteria	Mycobacterium abscessus
tr|H6R1K7|H6R1K7_NOCCG	2	Actinobacteria	Actinobacteria	Nocardia cyriacigeorgica
tr|K0UZ20|K0UZ20_MYCVA	2	Actinobacteria	Actinobacteria	Mycobacterium vaccae
tr|F5YUN8|F5YUN8_MYCSD	2	Actinobacteria	Actinobacteria	Mycobacterium sp. JDM601
tr|G7GTS9|G7GTS9_9ACTO	2	Actinobacteria	Actinobacteria	Gordonia amarae
tr|D0L6Z9|D0L6Z9_GORB4	2	Actinobacteria	Actinobacteria	Gordonia bronchialis
tr|F4CXJ3|F4CXJ3_PSEUX	2	Actinobacteria	Actinobacteria	Pseudonocardia dioxanivorans
tr|X8CVC8|X8CVC8_MYCIT	12	Actinobacteria	Actinobacteria	Mycobacterium intracellulare

tr|E9V1A0|E9V1A0_9ACTO	2	Actinobacteria	Actinobacteria	Nocardioidaceae bacterium Broad-1
tr|K6XI89|K6XI89_9ACTO	2	Actinobacteria	Actinobacteria	Gordonia namibiensis
tr|W5WIN9|W5WIN9_9PSEU	2	Actinobacteria	Actinobacteria	Kutzneria albida
tr|D1A3J0|D1A3J0_THECD	2	Actinobacteria	Actinobacteria	Thermomonospora curvata
tr|R6ABR7|R6ABR7_9PROT	1	Proteobacteria		Proteobacteria bacterium CAG:139
tr|R6ACY1|R6ACY1_9PROT	1	Proteobacteria		Proteobacteria bacterium CAG:139
tr|C1DCY3|C1DCY3_LARHH	1	Proteobacteria	Betaproteobacteria	Laribacter hongkongensis
tr|X7R9Z9|X7R9Z9_VIBPH	1	Proteobacteria	Gammaproteobacteria	Vibrio parahaemolyticus
tr|W9VAF1|W9VAF1_9GAMM	1	Proteobacteria	Gammaproteobacteria	Thiorhodococcus sp. AK35
tr|W0DNQ7|W0DNQ7_9GAMM	1	Proteobacteria	Gammaproteobacteria	Thioalkalivibrio thiocyanoxidans
tr|C0QIN1|C0QIN1_DESAH	1	Proteobacteria	Deltaproteobacteria	Desulfobacterium autotrophicum
tr|N1WBP2|N1WBP2_9LEPT	1	Spirochaetes	Spirochaetia	Leptospira vanthielii
tr|H1G2K8|H1G2K8_9GAMM	1	Proteobacteria	Gammaproteobacteria	Ectothiorhodospira sp. PHS-1
tr|A0A066PRJ9|A0A066PRJ9_9PROT	1	Proteobacteria	Alphaproteobacteria	Acidiphilium sp. JA12-A1
tr|G4E6M2|G4E6M2_9GAMM	1	Proteobacteria	Gammaproteobacteria	Thiorhodospira sibirica
tr|A4SXS4|A4SXS4_POLSQ	1	Proteobacteria	Betaproteobacteria	Polynucleobacter necessarius
tr|B9XQZ7|B9XQZ7_PEDPL	1	Verrucomicrobia	Verrucomicrobiae	Pedosphaera parvula
tr|F0JL42|F0JL42_DESDE	1	Proteobacteria	Deltaproteobacteria	Desulfovibrio desulfuricans
tr|Q1NX33|Q1NX33_9DELT	1	Proteobacteria	Deltaproteobacteria	delta proteobacterium MLMS-1
tr|F3Z0E1|F3Z0E1_DESAF	1	Proteobacteria	Deltaproteobacteria	Desulfovibrio africanus
tr|C6C1P5|C6C1P5_DESAD	1	Proteobacteria	Deltaproteobacteria	Desulfovibrio salexigens
tr|H0A0J8|H0A0J8_9PROT	1	Proteobacteria	Alphaproteobacteria	Acetobacteraceae bacterium AT-5844
tr|K1Z964|K1Z964_9BACT	1			uncultured bacterium
tr|A0A0B0EB06|A0A0B0EB06_9BACT	1	Planctomycetes	Planctomycetia	Candidatus Scalindua brodae
tr|K0NPF9|K0NPF9_DESTT	1	Proteobacteria	Deltaproteobacteria	Desulfobacula toluolica
tr|I5B7B9|I5B7B9_9DELT	1	Proteobacteria	Deltaproteobacteria	Desulfobacter postgatei
tr|D5H5N7|D5H5N7_SALRM	1	Bacteroidetes		Salinibacter ruber

tr|Q3A0X1|Q3A0X1_PELCD	1	Proteobacteria	Deltaproteobacteria	Pelobacter carbinolicus
tr|X0XD94|X0XD94_9ZZZZ	1			marine sediment metagenome
tr|Q5NTF0|Q5NTF0_9BACT	1			uncultured bacterium
tr|S0G044|S0G044_9DELT	1	Proteobacteria	Deltaproteobacteria	Desulfotignum phosphitoxidans
tr|I6B3M6|I6B3M6_9BACT	1	Verrucomicrobia	Opitutae	Opitutaceae bacterium TAV1
tr|A0A066TEL1|A0A066TEL1_9GAMM	1	Proteobacteria	Gammaproteobacteria	Gilliamella apicola
tr|U1KNB8|U1KNB8_9GAMM	1	Proteobacteria	Gammaproteobacteria	Pseudoalteromonas citrea
tr|H5T8I0|H5T8I0_9ALTE	1	Proteobacteria	Gammaproteobacteria	Glaciecola punicea
tr|T1CNB6|T1CNB6_9ZZZZ	1			mine drainage metagenome
tr|R5XY65|R5XY65_9PROT	1	Proteobacteria	Alphaproteobacteria	Acetobacter sp. CAG:267
tr|W6KJD9|W6KJD9_9PROT	1	Proteobacteria	Alphaproteobacteria	Magnetospira sp. QH-2
tr|C6V519|C6V519_NEORI	1	Proteobacteria	Alphaproteobacteria	Neorickettsia risticii
tr|H6SP71|H6SP71_RHOPH	1	Proteobacteria	Alphaproteobacteria	Rhodospirillum photometricum
tr|A0A076ECD6|A0A076ECD6_RHOOP	1	Actinobacteria	Actinobacteria	Rhodococcus opacus
tr|A0A0B8N996|A0A0B8N996_9NOCA	1	Actinobacteria	Actinobacteria	Nocardia seriolae
tr|V5XHK3|V5XHK3_MYCNE	1	Actinobacteria	Actinobacteria	Mycobacterium neoaurum
tr|M0QIQ5|M0QIQ5_9ACTO	1	Actinobacteria	Actinobacteria	Gordonia soli
tr|E6JDS4|E6JDS4_9ACTO	1	Actinobacteria	Actinobacteria	Dietzia cinnamea
tr|J5E3K3|J5E3K3_9MYCO	2	Actinobacteria	Actinobacteria	Mycobacterium colombiense
tr|W8HDM4|W8HDM4_RHOOP	1	Actinobacteria	Actinobacteria	Rhodococcus opacus
tr|A0A024K3A8|A0A024K3A8_9MYCO	1	Actinobacteria	Actinobacteria	Mycobacterium triplex
tr|G7CJW9|G7CJW9_MYCTH	1	Actinobacteria	Actinobacteria	Mycobacterium thermoresistibile
tr|Q7D9K7|Q7D9K7_MYCTO	1	Actinobacteria	Actinobacteria	Mycobacterium tuberculosis
tr|G7GUZ9|G7GUZ9_9ACTO	1	Actinobacteria	Actinobacteria	Gordonia amarae
tr|W5TFD9|W5TFD9_9NOCA	1	Actinobacteria	Actinobacteria	Nocardia nova
tr|A0A076N1F8|A0A076N1F8_AMYME	2	Actinobacteria	Actinobacteria	Amycolatopsis methanolica
tr|X8A2N5|X8A2N5_MYCAV	1	Actinobacteria	Actinobacteria	Mycobacterium avium

tr|X8AE45|X8AE45_MYCAV	2	Actinobacteria	Actinobacteria	Mycobacterium avium
tr|L7L6H6|L7L6H6_9ACTO	1	Actinobacteria	Actinobacteria	Gordonia hirsuta
tr|U5ECA5|U5ECA5_NOCAS	1	Actinobacteria	Actinobacteria	Nocardia asteroides
tr|G7GKG8|G7GKG8_9ACTO	1	Actinobacteria	Actinobacteria	Gordonia amarae
tr|G7H1J2|G7H1J2_9ACTO	1	Actinobacteria	Actinobacteria	Gordonia araii
tr|V5X6N2|V5X6N2_MYCNE	1	Actinobacteria	Actinobacteria	Mycobacterium neoaurum
tr|I0WL21|I0WL21_9NOCA	1	Actinobacteria	Actinobacteria	Rhodococcus imtechensis
tr|W5WJM4|W5WJM4_9PSEU	1	Actinobacteria	Actinobacteria	Kutzneria albida
tr|E9V0Y7|E9V0Y7_9ACTO	1	Actinobacteria	Actinobacteria	Nocardioidaceae bacterium Broad-1
tr|D1A398|D1A398_THECD	1	Actinobacteria	Actinobacteria	Thermomonospora curvata
tr|R7Y399|R7Y399_9ACTO	1	Actinobacteria	Actinobacteria	Gordonia terrae
tr|E2S876|E2S876_9ACTO	1	Actinobacteria	Actinobacteria	Aeromicrobium marinum
tr|H5TSB2|H5TSB2_9ACTO	1	Actinobacteria	Actinobacteria	Gordonia otitidis
tr|W7IML6|W7IML6_9PSEU	1	Actinobacteria	Actinobacteria	Actinokineospora sp. EG49
tr|X7YBW9|X7YBW9_MYCKA	1	Actinobacteria	Actinobacteria	Mycobacterium kansasii
tr|W7SAJ9|W7SAJ9_9PSEU	1	Actinobacteria	Actinobacteria	Kutzneria sp. 744
tr|D2PU98|D2PU98_KRIFD	1	Actinobacteria	Actinobacteria	Kribbella flavida
tr|J2K2G4|J2K2G4_9ACTO	1	Actinobacteria	Actinobacteria	Streptomyces auratus
tr|D3QB66|D3QB66_STANL	2	Actinobacteria	Actinobacteria	Stackebrandtia nassauensis
tr|E6S9N4|E6S9N4_INTC7	1	Actinobacteria	Actinobacteria	Intrasporangium calvum
tr|E9UWC3|E9UWC3_9ACTO	1	Actinobacteria	Actinobacteria	Nocardioidaceae bacterium Broad-1
tr|A0A0A1DJ20|A0A0A1DJ20_NOCSI	1	Actinobacteria	Actinobacteria	Pimelobacter simplex
tr|H6RMU6|H6RMU6_BLASD	2	Actinobacteria	Actinobacteria	Blastococcus saxobsidens
tr|D6KCL8|D6KCL8_9ACTO	1	Actinobacteria	Actinobacteria	Streptomyces sp. e14
tr|A0A0B2BN83|A0A0B2BN83_9ACTO	1	Actinobacteria	Actinobacteria	Mumia flava
tr|M7AA00|M7AA00_9ACTO	1	Actinobacteria	Actinobacteria	Gordonia sp. NB4-1Y
tr|A0A0A1DLG3|A0A0A1DLG3_NOCSI	1	Actinobacteria	Actinobacteria	Pimelobacter simplex

tr|F6EHT6|F6EHT6_AMYSD	2	Actinobacteria	Actinobacteria	Amycolicicoccus subflavus
tr|U0E8H8|U0E8H8_9NOCA	1	Actinobacteria	Actinobacteria	Rhodococcus sp. P27
tr|A0A0C1L1Y7|A0A0C1L1Y7_9PSEU	2	Actinobacteria	Actinobacteria	Prauserella sp. Am3
tr|A0A0A1DM07|A0A0A1DM07_NOCSI	1	Actinobacteria	Actinobacteria	Pimelobacter simplex
tr|L2T7U8|L2T7U8_9NOCA	1	Actinobacteria	Actinobacteria	Rhodococcus wratislaviensis
tr|E2SGA8|E2SGA8_9ACTO	2	Actinobacteria	Actinobacteria	Aeromicrobium marinum
tr|R7XXX5|R7XXX5_9ACTO	2	Actinobacteria	Actinobacteria	Nocardioides sp. CF8
tr|L8DA69|L8DA69_9NOCA	1	Actinobacteria	Actinobacteria	Rhodococcus sp. AW25M09
tr|E2SCR0|E2SCR0_9ACTO	1	Actinobacteria	Actinobacteria	Aeromicrobium marinum
tr|A0A059MGR1|A0A059MGR1_9NOCA	2	Actinobacteria	Actinobacteria	Rhodococcus sp. BCP1
tr|C1B006|C1B006_RHOOB	1	Actinobacteria	Actinobacteria	Rhodococcus opacus
tr|C6WH39|C6WH39_ACTMD	1	Actinobacteria	Actinobacteria	Actinosynnema mirum
tr|H5X8G3|H5X8G3_9PSEU	1	Actinobacteria	Actinobacteria	Saccharomonospora marina
tr|A0A073BDE9|A0A073BDE9_9PSEU	2	Actinobacteria	Actinobacteria	Saccharopolyspora rectivirgula
tr|H5X5R0|H5X5R0_9PSEU	1	Actinobacteria	Actinobacteria	Saccharomonospora marina
tr|K6VSM1|K6VSM1_9ACTO	1	Actinobacteria	Actinobacteria	Gordonia rhizosphera
tr|L8DGB2|L8DGB2_9NOCA	1	Actinobacteria	Actinobacteria	Rhodococcus sp. AW25M09
tr|E9UWP1|E9UWP1_9ACTO	1	Actinobacteria	Actinobacteria	Nocardioidaceae bacterium Broad-1
tr|A0A0A1FX38|A0A0A1FX38_9MYCO	1	Actinobacteria	Actinobacteria	Mycobacterium sp. VKM Ac-1817D
tr|A0A088DHD2|A0A088DHD2_MYCAV	1	Actinobacteria	Actinobacteria	Mycobacterium avium
tr|H0R665|H0R665_9ACTO	1	Actinobacteria	Actinobacteria	Gordonia effusa
tr|X8DHC4|X8DHC4_MYCAB	1	Actinobacteria	Actinobacteria	Mycobacterium abscessus
tr|A0A0B8NJT9|A0A0B8NJT9_9NOCA	1	Actinobacteria	Actinobacteria	Nocardia seriolae
tr|M2XN33|M2XN33_9NOCA	1	Actinobacteria	Actinobacteria	Rhodococcus qingshengii
tr|H0R4X2|H0R4X2_9ACTO	1	Actinobacteria	Actinobacteria	Gordonia effusa
tr|W5TQG3|W5TQG3_9NOCA	1	Actinobacteria	Actinobacteria	Nocardia nova
tr|U5EGV5|U5EGV5_NOCAS	1	Actinobacteria	Actinobacteria	Nocardia asteroides

tr|G7GTT4|G7GTT4_9ACTO	1	Actinobacteria	Actinobacteria	Gordonia amarae
tr|R4UK58|R4UK58_MYCAB	1	Actinobacteria	Actinobacteria	Mycobacterium abscessus
tr|A0A0A1DM34|A0A0A1DM34_NOCSI	1	Actinobacteria	Actinobacteria	Pimelobacter simplex
tr|A0A049DP51|A0A049DP51_MYCAV	1	Actinobacteria	Actinobacteria	Mycobacterium avium
tr|D5UP37|D5UP37_TSUPD	1	Actinobacteria	Actinobacteria	Tsukamurella paurometabola
tr|A0A0B8KPG8|A0A0B8KPG8_MYCPC	1	Actinobacteria	Actinobacteria	Mycobacterium avium
tr|A0A0B8NMN5|A0A0B8NMN5_9NOCA	1	Actinobacteria	Actinobacteria	Nocardia seriolae
tr|L7KYU3|L7KYU3_9ACTO	1	Actinobacteria	Actinobacteria	Gordonia amicalis
tr|X8DX53|X8DX53_MYCAB	1	Actinobacteria	Actinobacteria	Mycobacterium abscessus
tr|F1YGH6|F1YGH6_9ACTO	1	Actinobacteria	Actinobacteria	Gordonia neofelifaecis
tr|G7H7G3|G7H7G3_9ACTO	1	Actinobacteria	Actinobacteria	Gordonia araii
tr|U5EFA8|U5EFA8_NOCAS	1	Actinobacteria	Actinobacteria	Nocardia asteroides
tr|H0QYR9|H0QYR9_9ACTO	1	Actinobacteria	Actinobacteria	Gordonia effusa
tr|A0A062XQ08|A0A062XQ08_9BACT	1	Acidobacteria		Thermoanaerobaculum aquaticum
tr|A0A084T2A7|A0A084T2A7_9DELT	1	Proteobacteria	Deltaproteobacteria	Cystobacter violaceus
tr|I3IJ79|I3IJ79_9PLAN	1	Planctomycetes	Planctomycetia	planctomycete KSU-1
tr|G2LF04|G2LF04_CHLTF	1	Acidobacteria		Candidatus Chloracidobacterium thermophilum
tr|A9F8B6|A9F8B6_SORC5	1	Proteobacteria	Deltaproteobacteria	Sorangium cellulosum
tr|A0A0B5FRU7|A0A0B5FRU7_9DELT	1	Proteobacteria	Deltaproteobacteria	Geoalkalibacter subterraneus
tr|B9M828|B9M828_GEODF	1	Proteobacteria	Deltaproteobacteria	Geobacter daltonii
tr|Q1IHY3|Q1IHY3_KORVE	1	Acidobacteria	Acidobacteriia	Candidatus Koribacter versatilis
tr|E8V6D1|E8V6D1_TERSS	1	Acidobacteria	Acidobacteriia	Terriglobus saanensis
tr|F4C5F7|F4C5F7_SPHS2	1	Bacteroidetes	Sphingobacteriia	Sphingobacterium sp. 21
tr|A0A0B6X0V1|A0A0B6X0V1_9BACT	1	Acidobacteria		Pyrinomonas methylaliphatogenes
tr|I2GM83|I2GM83_9BACT	1	Bacteroidetes	Cytophagia	Fibrisoma limi
tr|Q02BI0|Q02BI0_SOLUE	1	Acidobacteria	Solibacteres	Candidatus Solibacter usitatus
tr|L0G5Y2|L0G5Y2_ECHVK	1	Bacteroidetes	Cytophagia	Echinicola vietnamensis

tr|I6ZXI2|I6ZXI2_MELRP	1	Ignavibacteriae	Ignavibacteria	Melioribacter roseus
tr|Q1PXQ9|Q1PXQ9_9BACT	1	Planctomycetes	Planctomycetia	Candidatus Kuenenia stuttgartiensis
tr|U2CPG0|U2CPG0_9BACE	1	Bacteroidetes	Bacteroidia	Bacteroides pyogenes
tr|K1ZAX2|K1ZAX2_9BACT	1			uncultured bacterium
tr|W5X224|W5X224_BDEBC	1	Proteobacteria	Deltaproteobacteria	Bdellovibrio bacteriovorus
tr|A0A098LE12|A0A098LE12_9BACT	1	Bacteroidetes	Cytophagia	Sporocytophaga myxococcoides
tr|G8NWH9|G8NWH9_GRAMM	1	Acidobacteria	Acidobacteriia	Granulicella mallensis
tr|A0A0A8WWS4|A0A0A8WWS4_9DELT	1	Proteobacteria	Deltaproteobacteria	Geobacter sp. OR-1
tr|G8TBV3|G8TBV3_NIAKG	1	Bacteroidetes	Sphingobacteriia	Niastella koreensis
tr|J0LBB1|J0LBB1_9BACT	1	Bacteroidetes	Cytophagia	Pontibacter sp. BAB1700
tr|F8EIQ2|F8EIQ2_RUNSL	1	Bacteroidetes	Cytophagia	Runella slithyformis
tr|H8KQ18|H8KQ18_SOLCM	1	Bacteroidetes	Sphingobacteriia	Solitalea canadensis
tr|E6QM46|E6QM46_9ZZZZ	1			mine drainage metagenome
tr|C6VWY6|C6VWY6_DYAFD	1	Bacteroidetes	Cytophagia	Dyadobacter fermentans
tr|A0A066WL26|A0A066WL26_9FLAO	1	Bacteroidetes	Flavobacteriia	Flavobacterium sp. EM1321
tr|Q11QT9|Q11QT9_CYTH3	1	Bacteroidetes	Cytophagia	Cytophaga hutchinsonii
tr|H1YG72|H1YG72_9SPHI	1	Bacteroidetes	Sphingobacteriia	Mucilaginibacter paludis
tr|A0A0C1U0Z5|A0A0C1U0Z5_9CYAN	1	Cyanobacteria		Hassallia byssoidea
tr|G8TB64|G8TB64_NIAKG	1	Bacteroidetes	Sphingobacteriia	Niastella koreensis
tr|I4C8D1|I4C8D1_DESTA	1	Proteobacteria	Deltaproteobacteria	Desulfomonile tiedjei
tr|B9XI42|B9XI42_PEDPL	1	Verrucomicrobia	Verrucomicrobiae	Pedosphaera parvula
tr|D7VTS5|D7VTS5_9SPHI	1	Bacteroidetes	Sphingobacteriia	Sphingobacterium spiritivorum
tr|A0A083WVJ2|A0A083WVJ2_9FLAO	1	Bacteroidetes	Flavobacteriia	Chryseobacterium antarcticum
tr|H1XU60|H1XU60_9BACT	1			Caldithrix abyssi
tr|C7PRY9|C7PRY9_CHIPD	1	Bacteroidetes	Sphingobacteriia	Chitinophaga pinensis
tr|I0AH89|I0AH89_IGNAJ	1	Ignavibacteriae	Ignavibacteria	Ignavibacterium album
tr|I6ZZU2|I6ZZU2_MELRP	1	Ignavibacteriae	Ignavibacteria	Melioribacter roseus

tr|J9ZBK3|J9ZBK3_LEPFM	1	Nitrospirae	Nitrospira	Leptospirillum ferriphilum
tr|C0INF9|C0INF9_9BACT	1			uncultured bacterium BLR12
tr|F4L5C0|F4L5C0_HALH1	1	Bacteroidetes	Sphingobacteriia	Haliscomenobacter hydrossis
tr|G2SGS9|G2SGS9_RHOMR	1	Bacteroidetes		Rhodothermus marinus
tr|Q01T55|Q01T55_SOLUE	1	Acidobacteria	Solibacteres	Candidatus Solibacter usitatus
tr|A5FBV3|A5FBV3_FLAJ1	1	Bacteroidetes	Flavobacteriia	Flavobacterium johnsoniae
tr|A0A085EHM6|A0A085EHM6_9FLAO	1	Bacteroidetes	Flavobacteriia	Flavobacterium sp. EM1308
tr|W0RKP5|W0RKP5_9BACT	1	Gemmatimonadetes	Gemmatimonadetes	Gemmatimonadetes bacterium KBS708
tr|H2BUH3|H2BUH3_9FLAO	1	Bacteroidetes	Flavobacteriia	Gillisia limnaea
tr|A0A062XY82|A0A062XY82_9BACT	1	Acidobacteria		Thermoanaerobaculum aquaticum
tr|B3ELU9|B3ELU9_CHLPB	1	Chlorobi	Chlorobia	Chlorobium phaeobacteroides
tr|W8F729|W8F729_9BACT	1	Bacteroidetes	Cytophagia	Hymenobacter swuensis
tr|M7NHF3|M7NHF3_9BACT	1	Bacteroidetes	Cytophagia	Cesiribacter andamanensis
tr|C7PSE8|C7PSE8_CHIPD	1	Bacteroidetes	Sphingobacteriia	Chitinophaga pinensis
tr|F0S3N0|F0S3N0_DESTD	1	Aquificae	Aquificae	Desulfurobacterium thermolithotrophum
tr|C1DTQ3|C1DTQ3_SULAA	1	Aquificae	Aquificae	Sulfurihydrogenibium azorense
tr|A6ECD4|A6ECD4_9SPHI	1	Bacteroidetes	Sphingobacteriia	Pedobacter sp. BAL39
tr|B4U8R4|B4U8R4_HYDS0	1	Aquificae	Aquificae	Hydrogenobaculum sp. Y04AAS1
tr|A0A0C1KVI4|A0A0C1KVI4_9SPHI	1	Bacteroidetes	Sphingobacteriia	Flavihumibacter sp. ZG627
tr|G2DDR7|G2DDR7_9GAMM	1	Proteobacteria	Gammaproteobacteria	endosymbiont of Riftia pachyptila
tr|G4WVV9|G4WVV9_9BACT	18			uncultured bacterium CSLD10
tr|M7XAT5|M7XAT5_9BACT	1	Bacteroidetes	Cytophagia	Mariniradius saccharolyticus
tr|A0A0C1KRV9|A0A0C1KRV9_9SPHI	1	Bacteroidetes	Sphingobacteriia	Flavihumibacter sp. ZG627
tr|A0LL26|A0LL26_SYNFM	1	Proteobacteria	Deltaproteobacteria	Syntrophobacter fumaroxidans
tr|A0A099TQ66|A0A099TQ66_9HELI	1	Proteobacteria	Epsilonproteobacteria	Helicobacter sp. MIT 11-5569
tr|V8CB90|V8CB90_9HELI	1	Proteobacteria	Epsilonproteobacteria	Helicobacter macacae
tr|D3UJD6|D3UJD6_HELM1	1	Proteobacteria	Epsilonproteobacteria	Helicobacter mustelae

tr|A0A0B2EM49|A0A0B2EM49_HELPX	1	Proteobacteria	Epsilonproteobacteria	Helicobacter pylori
tr|T1CYV8|T1CYV8_9HELI	1	Proteobacteria	Epsilonproteobacteria	Helicobacter fennelliae
tr|A0A099VAL0|A0A099VAL0_9HELI	1	Proteobacteria	Epsilonproteobacteria	Helicobacter trogontum
tr|M2YFM5|M2YFM5_9PSEU	2	Actinobacteria	Actinobacteria	Amycolatopsis decaplanina
tr|F3ZJE5|F3ZJE5_9ACTO	2	Actinobacteria	Actinobacteria	Streptomyces sp. Tu6071
tr|E2PY84|E2PY84_STRC2	1	Actinobacteria	Actinobacteria	Streptomyces clavuligerus
tr|A0A066Y802|A0A066Y802_9ACTO	2	Actinobacteria	Actinobacteria	Streptomyces olindensis
tr|H6RMU7|H6RMU7_BLASD	2	Actinobacteria	Actinobacteria	Blastococcus saxobsidens
tr|D1A399|D1A399_THECD	2	Actinobacteria	Actinobacteria	Thermomonospora curvata
tr|D2PU99|D2PU99_KRIFD	2	Actinobacteria	Actinobacteria	Kribbella flavida
tr|E2SGA9|E2SGA9_9ACTO	1	Actinobacteria	Actinobacteria	Aeromicrobium marinum
tr|A0A0A1DGK3|A0A0A1DGK3_NOCSI	2	Actinobacteria	Actinobacteria	Pimelobacter simplex
tr|V9XBJ1|V9XBJ1_9NOCA	2	Actinobacteria	Actinobacteria	Rhodococcus pyridinivorans
tr|A1SKI7|A1SKI7_NOCSJ	1	Actinobacteria	Actinobacteria	Nocardioides sp. JS614
tr|F6EHT5|F6EHT5_AMYSD	2	Actinobacteria	Actinobacteria	Amycolicicoccus subflavus
tr|A0A022LT29|A0A022LT29_9ACTO	2	Actinobacteria	Actinobacteria	Dietzia sp. UCD-THP
tr|D5UQ83|D5UQ83_TSUPD	2	Actinobacteria	Actinobacteria	Tsukamurella paurometabola
tr|E9UWC4|E9UWC4_9ACTO	1	Actinobacteria	Actinobacteria	Nocardioidaceae bacterium Broad-1
tr|R7Y156|R7Y156_9ACTO	1	Actinobacteria	Actinobacteria	Nocardioides sp. CF8
tr|D3QB65|D3QB65_STANL	2	Actinobacteria	Actinobacteria	Stackebrandtia nassauensis
tr|C1BDE9|C1BDE9_RHOOB	2	Actinobacteria	Actinobacteria	Rhodococcus opacus
tr|T5HZF3|T5HZF3_RHOER	1	Actinobacteria	Actinobacteria	Rhodococcus erythropolis
tr|H5X8G2|H5X8G2_9PSEU	2	Actinobacteria	Actinobacteria	Saccharomonospora marina
tr|E2SEJ6|E2SEJ6_9ACTO	1	Actinobacteria	Actinobacteria	Aeromicrobium marinum
tr|G7GQS7|G7GQS7_9ACTO	1	Actinobacteria	Actinobacteria	Gordonia amarae
tr|E2S875|E2S875_9ACTO	1	Actinobacteria	Actinobacteria	Aeromicrobium marinum
tr|A0R1K5|A0R1K5_MYCS2	2	Actinobacteria	Actinobacteria	Mycobacterium smegmatis

tr|M2XH03|M2XH03_9NOCA	2	Actinobacteria	Actinobacteria	Rhodococcus triatomae
tr|R7XXR3|R7XXR3_9ACTO	2	Actinobacteria	Actinobacteria	Nocardioides sp. CF8
tr|E6JDS5|E6JDS5_9ACTO	1	Actinobacteria	Actinobacteria	Dietzia cinnamea
tr|L7KG85|L7KG85_9ACTO	2	Actinobacteria	Actinobacteria	Gordonia aichiensis
tr|F8B413|F8B413_FRADG	2	Actinobacteria	Actinobacteria	Frankia symbiont of Datisca glomerata
tr|A0A076N0Q6|A0A076N0Q6_AMYME	1	Actinobacteria	Actinobacteria	Amycolatopsis methanolica
tr|A0A076EAH3|A0A076EAH3_RHOOP	1	Actinobacteria	Actinobacteria	Rhodococcus opacus
tr|E9V0Y8|E9V0Y8_9ACTO	1	Actinobacteria	Actinobacteria	Nocardioidaceae bacterium Broad-1
tr|A0A051UJJ6|A0A051UJJ6_MYCTX	2	Actinobacteria	Actinobacteria	Mycobacterium tuberculosis
tr|V7JUV1|V7JUV1_MYCPC	2	Actinobacteria	Actinobacteria	Mycobacterium avium
tr|I7FLI0|I7FLI0_MYCS2	1	Actinobacteria	Actinobacteria	Mycobacterium smegmatis
tr|L7KCE3|L7KCE3_GORRU	2	Actinobacteria	Actinobacteria	Gordonia rubripertincta
tr|A0QMJ3|A0QMJ3_MYCA1	1	Actinobacteria	Actinobacteria	Mycobacterium avium
tr|G7GKE5|G7GKE5_9ACTO	2	Actinobacteria	Actinobacteria	Gordonia amarae
tr|G7GSR2|G7GSR2_9ACTO	1	Actinobacteria	Actinobacteria	Gordonia amarae
tr|A0A0A1DJ08|A0A0A1DJ08_NOCSI	1	Actinobacteria	Actinobacteria	Pimelobacter simplex
tr|M0QIE3|M0QIE3_9ACTO	2	Actinobacteria	Actinobacteria	Gordonia soli
tr|A0A0A1DPF3|A0A0A1DPF3_NOCSI	1	Actinobacteria	Actinobacteria	Pimelobacter simplex
tr|H5X5Q9|H5X5Q9_9PSEU	2	Actinobacteria	Actinobacteria	Saccharomonospora marina
tr|V8CVU7|V8CVU7_9ACTO	1	Actinobacteria	Actinobacteria	Williamsia sp. D3
tr|E9UWP2|E9UWP2_9ACTO	2	Actinobacteria	Actinobacteria	Nocardioidaceae bacterium Broad-1
tr|W5TBZ3|W5TBZ3_9NOCA	1	Actinobacteria	Actinobacteria	Nocardia nova
tr|H0R664|H0R664_9ACTO	1	Actinobacteria	Actinobacteria	Gordonia effusa
tr|H6R4G2|H6R4G2_NOCCG	1	Actinobacteria	Actinobacteria	Nocardia cyriacigeorgica
tr|E2SCQ9|E2SCQ9_9ACTO	1	Actinobacteria	Actinobacteria	Aeromicrobium marinum
tr|W5TF23|W5TF23_9NOCA	1	Actinobacteria	Actinobacteria	Nocardia nova
tr|Q0S3I4|Q0S3I4_RHOJR	2	Actinobacteria	Actinobacteria	Rhodococcus jostii

tr|A0A0B8NHK4|A0A0B8NHK4_9NOCA	2	Actinobacteria	Actinobacteria	Nocardia seriolae
tr|A0A0C2VT22|A0A0C2VT22_RHOER	2	Actinobacteria	Actinobacteria	Rhodococcus erythropolis
tr|W5TJG0|W5TJG0_9NOCA	1	Actinobacteria	Actinobacteria	Nocardia nova
tr|A0A088DKD4|A0A088DKD4_MYCAV	1	Actinobacteria	Actinobacteria	Mycobacterium avium
tr|L8DBZ0|L8DBZ0_9NOCA	1	Actinobacteria	Actinobacteria	Rhodococcus sp. AW25M09
tr|A0A073B2F2|A0A073B2F2_9PSEU	2	Actinobacteria	Actinobacteria	Saccharopolyspora rectivirgula
tr|G7GTT3|G7GTT3_9ACTO	1	Actinobacteria	Actinobacteria	Gordonia amarae
tr|M2VE22|M2VE22_9NOCA	1	Actinobacteria	Actinobacteria	Rhodococcus triatomae
tr|H5U070|H5U070_9ACTO	2	Actinobacteria	Actinobacteria	Gordonia sputi
tr|K6XRH0|K6XRH0_9ACTO	1	Actinobacteria	Actinobacteria	Gordonia namibiensis
tr|F5Z0C0|F5Z0C0_MYCSD	1	Actinobacteria	Actinobacteria	Mycobacterium sp. JDM601
tr|X8EUF9|X8EUF9_MYCCH	1	Actinobacteria	Actinobacteria	Mycobacterium chelonae
tr|L8DK68|L8DK68_9NOCA	1	Actinobacteria	Actinobacteria	Rhodococcus sp. AW25M09
tr|H0QWF8|H0QWF8_9ACTO	1	Actinobacteria	Actinobacteria	Gordonia effusa
tr|D5UP36|D5UP36_TSUPD	1	Actinobacteria	Actinobacteria	Tsukamurella paurometabola
tr|X8AND8|X8AND8_MYCAV	6	Actinobacteria	Actinobacteria	Mycobacterium avium
tr|D5UYS0|D5UYS0_TSUPD	1	Actinobacteria	Actinobacteria	Tsukamurella paurometabola
tr|H6MXF1|H6MXF1_GORPV	1	Actinobacteria	Actinobacteria	Gordonia polyisoprenivorans
tr|D5UW65|D5UW65_TSUPD	1	Actinobacteria	Actinobacteria	Tsukamurella paurometabola
tr|K6VQS7|K6VQS7_9ACTO	1	Actinobacteria	Actinobacteria	Gordonia namibiensis
tr|H5UAB4|H5UAB4_9ACTO	1	Actinobacteria	Actinobacteria	Gordonia terrae
tr|F6EN11|F6EN11_AMYSD	1	Actinobacteria	Actinobacteria	Amycolicicoccus subflavus
tr|L7KVP1|L7KVP1_9ACTO	1	Actinobacteria	Actinobacteria	Gordonia amicalis
tr|K5B990|K5B990_9MYCO	1	Actinobacteria	Actinobacteria	Mycobacterium hassiacum
tr|R7XXT4|R7XXT4_9ACTO	1	Actinobacteria	Actinobacteria	Nocardioides sp. CF8
tr|L7LAC8|L7LAC8_9ACTO	1	Actinobacteria	Actinobacteria	Gordonia hirsuta
tr|R7XYT4|R7XYT4_9ACTO	1	Actinobacteria	Actinobacteria	Nocardioides sp. CF8

tr|X8CS24|X8CS24_MYCIT	12	Actinobacteria	Actinobacteria	Mycobacterium intracellulare
tr|A0A098BQC5|A0A098BQC5_9NOCA	1	Actinobacteria	Actinobacteria	Rhodococcus ruber
tr|E9V0Z1|E9V0Z1_9ACTO	1	Actinobacteria	Actinobacteria	Nocardioidaceae bacterium Broad-1
tr|H0QWP0|H0QWP0_9ACTO	1	Actinobacteria	Actinobacteria	Gordonia effusa
tr|A0A076MVD4|A0A076MVD4_AMYME	1	Actinobacteria	Actinobacteria	Amycolatopsis methanolica
tr|R7Y2Z7|R7Y2Z7_9ACTO	1	Actinobacteria	Actinobacteria	Gordonia terrae
tr|H0RGF2|H0RGF2_9ACTO	1	Actinobacteria	Actinobacteria	Gordonia polyisoprenivorans
tr|I0PGQ2|I0PGQ2_MYCAB	1	Actinobacteria	Actinobacteria	Mycobacterium abscessus
tr|D2B9V7|D2B9V7_STRRD	1	Actinobacteria	Actinobacteria	Streptosporangium roseum
tr|G7GP52|G7GP52_9ACTO	2	Actinobacteria	Actinobacteria	Gordonia amarae
tr|L7KU47|L7KU47_9ACTO	1	Actinobacteria	Actinobacteria	Gordonia amicalis
tr|H5X5Q6|H5X5Q6_9PSEU	1	Actinobacteria	Actinobacteria	Saccharomonospora marina
tr|W5TGX9|W5TGX9_9NOCA	1	Actinobacteria	Actinobacteria	Nocardia nova
tr|G7GP48|G7GP48_9ACTO	1	Actinobacteria	Actinobacteria	Gordonia amarae
tr|Q5YVH2|Q5YVH2_NOCFA	1	Actinobacteria	Actinobacteria	Nocardia farcinica
tr|W7STV8|W7STV8_9PSEU	1	Actinobacteria	Actinobacteria	Kutzneria sp. 744
tr|F5Z0B7|F5Z0B7_MYCSD	1	Actinobacteria	Actinobacteria	Mycobacterium sp. JDM601
tr|D3QB62|D3QB62_STANL	1	Actinobacteria	Actinobacteria	Stackebrandtia nassauensis
tr|K0EUQ9|K0EUQ9_9NOCA	1	Actinobacteria	Actinobacteria	Nocardia brasiliensis
tr|H0RG52|H0RG52_9ACTO	1	Actinobacteria	Actinobacteria	Gordonia polyisoprenivorans
tr|A0A076MRM1|A0A076MRM1_AMYME	1	Actinobacteria	Actinobacteria	Amycolatopsis methanolica
tr|H6MXF4|H6MXF4_GORPV	1	Actinobacteria	Actinobacteria	Gordonia polyisoprenivorans
tr|R4LVY7|R4LVY7_MYCTX	6	Actinobacteria	Actinobacteria	Mycobacterium tuberculosis
tr|G8RXW6|G8RXW6_MYCRN	1	Actinobacteria	Actinobacteria	Mycobacterium rhodesiae
tr|Q0S3I6|Q0S3I6_RHOJR	6	Actinobacteria	Actinobacteria	Rhodococcus jostii
tr|H6R8W6|H6R8W6_NOCCG	1	Actinobacteria	Actinobacteria	Nocardia cyriacigeorgica
tr|H0ITI3|H0ITI3_MYCAB	1	Actinobacteria	Actinobacteria	Mycobacterium abscessus

tr|V2PWJ5|V2PWJ5_9BACT	1	Deferribacteres	Deferribacteres	Mucispirillum schaedleri
tr|D3PEB4|D3PEB4_DEFDS	1	Deferribacteres	Deferribacteres	Deferribacter desulfuricans
tr|A0A068JLF4|A0A068JLF4_9DELT	1	Proteobacteria	Deltaproteobacteria	Desulfonatronum thiodismutans
tr|B3E1S0|B3E1S0_GEOLS	1	Proteobacteria	Deltaproteobacteria	Geobacter lovleyi
tr|A5GF79|A5GF79_GEOUR	1	Proteobacteria	Deltaproteobacteria	Geobacter uraniireducens
tr|D5CSN6|D5CSN6_SIDLE	1	Proteobacteria	Betaproteobacteria	Sideroxydans lithotrophicus
tr|B5YHU7|B5YHU7_THEYD	1	Nitrospirae	Nitrospira	Thermodesulfovibrio yellowstonii
tr|A0A099UEX8|A0A099UEX8_9HELI	1	Proteobacteria	Epsilonproteobacteria	Helicobacter typhlonius
tr|K1YVS7|K1YVS7_9BACT	1			uncultured bacterium
tr|M4VAD6|M4VAD6_9DELT	1	Proteobacteria	Deltaproteobacteria	Bdellovibrio exovorus
tr|D4H824|D4H824_DENA2	1	Deferribacteres	Deferribacteres	Denitrovibrio acetiphilus
tr|H8N0I9|H8N0I9_CORCM	1	Proteobacteria	Deltaproteobacteria	Corallococcus coralloides
tr|T0RH11|T0RH11_9DELT	1	Proteobacteria	Deltaproteobacteria	Bacteriovorax sp. Seq25_V
tr|E6W1R4|E6W1R4_DESIS	1	Chrysiogenetes	Chrysiogenetes	Desulfurispirillum indicum
tr|D0LYW5|D0LYW5_HALO1	1	Proteobacteria	Deltaproteobacteria	Haliangium ochraceum
tr|S4Y647|S4Y647_SORCE	1	Proteobacteria	Deltaproteobacteria	Sorangium cellulosum
tr|Q7MS05|Q7MS05_WOLSU	1	Proteobacteria	Epsilonproteobacteria	Wolinella succinogenes
tr|A6Q4N4|A6Q4N4_NITSB	1	Proteobacteria	Epsilonproteobacteria	Nitratiruptor sp. SB155-2
tr|K2AF75|K2AF75_9BACT	1			uncultured bacterium
tr|K7S285|K7S285_9HELI	1	Proteobacteria	Epsilonproteobacteria	Candidatus Sulfuricurvum sp. RIFRC-1
tr|A0A017TC91|A0A017TC91_9DELT	1	Proteobacteria	Deltaproteobacteria	Chondromyces apiculatus
tr|F5YBC2|F5YBC2_TREAZ	1	Spirochaetes	Spirochaetia	Treponema azotonutricium
tr|A6DA65|A6DA65_9PROT	1	Proteobacteria	Epsilonproteobacteria	Caminibacter mediatlanticus
tr|A0A094WAV2|A0A094WAV2_9BACT	1	Nitrospirae	Nitrospira	Leptospirillum ferriphilum
tr|F8F156|F8F156_TRECH	1	Spirochaetes	Spirochaetia	Treponema caldaria
tr|V2QE64|V2QE64_9BACT	1	Deferribacteres	Deferribacteres	Mucispirillum schaedleri
tr|C0QRE9|C0QRE9_PERMH	1	Aquificae	Aquificae	Persephonella marina

tr|A7HBT5|A7HBT5_ANADF	1	Proteobacteria	Deltaproteobacteria	Anaeromyxobacter sp. Fw109-5
tr|A0A0C2ZVP4|A0A0C2ZVP4_9HELI	1	Proteobacteria	Epsilonproteobacteria	Sulfuricurvum sp. PC08-66
tr|U2HVI4|U2HVI4_9SPHI	1	Bacteroidetes	Sphingobacteriia	Sphingobacterium paucimobilis
tr|C1AA19|C1AA19_GEMAT	1	Gemmatimonadetes	Gemmatimonadetes	Gemmatimonas aurantiaca
tr|E1R9E4|E1R9E4_SPISS	1	Spirochaetes	Spirochaetia	Spirochaeta smaragdinae
tr|J0L7H8|J0L7H8_9HELI	1	Proteobacteria	Epsilonproteobacteria	Thiovulum sp. ES
tr|A0A0C2YZI5|A0A0C2YZI5_9PROT	1	Proteobacteria	Epsilonproteobacteria	Sulfurovum sp. AS07-7
tr|A0A075KNH8|A0A075KNH8_9FIRM	1	Firmicutes	Negativicutes	Pelosinus sp. UFO1
tr|Q0EZD4|Q0EZD4_9PROT	1	Proteobacteria	Zetaproteobacteria	Mariprofundus ferrooxydans
tr|K4IK87|K4IK87_PSYTT	1	Bacteroidetes	Flavobacteriia	Psychroflexus torquis
tr|E4T8S3|E4T8S3_PALPW	1	Bacteroidetes	Bacteroidia	Paludibacter propionicigenes
tr|A0A078TMF9|A0A078TMF9_9PORP	1	Bacteroidetes	Bacteroidia	Parabacteroides distasonis
tr|V6DIG3|V6DIG3_9DELT	1	Proteobacteria	Deltaproteobacteria	Candidatus Babela massiliensis
tr|H3KA77|H3KA77_9FIRM	1	Firmicutes	Negativicutes	Megamonas funiformis
tr|K2DP81|K2DP81_9BACT	1			uncultured bacterium
tr|W0SHT2|W0SHT2_9RHOO	1	Proteobacteria	Betaproteobacteria	Sulfuritalea hydrogenivorans
tr|B3U4T4|B3U4T4_9BACT	1	Nitrospirae	Nitrospira	Candidatus Nitrospira defluvii
tr|C6I0D1|C6I0D1_9BACT	1	Nitrospirae	Nitrospira	Leptospirillum ferrodiazotrophum
tr|J3CHC9|J3CHC9_9FLAO	1	Bacteroidetes	Flavobacteriia	Chryseobacterium sp. CF314
tr|R5W525|R5W525_9BACT	1	Bacteroidetes	Bacteroidia	Alistipes sp. CAG:53
tr|H9UKN8|H9UKN8_SPIAZ	1	Spirochaetes	Spirochaetia	Spirochaeta africana
tr|A0A085B811|A0A085B811_9FLAO	1	Bacteroidetes	Flavobacteriia	Epilithonimonas lactis
tr|W6M797|W6M797_9GAMM	1	Proteobacteria	Gammaproteobacteria	Candidatus Contendobacter odensis
tr|A6G1B9|A6G1B9_9DELT	6	Proteobacteria	Deltaproteobacteria	Plesiocystis pacifica
tr|D9PM45|D9PM45_9ZZZZ	1			sediment metagenome
tr|K1LKK0|K1LKK0_9BACT	1	Bacteroidetes	Cytophagia	Cecembia lonarensis
tr|A0A0C1RTA3|A0A0C1RTA3_9BACT	1	Verrucomicrobia		Methylacidiphilum kamchatkense

tr|U5BSD5|U5BSD5_9BACT	1	Bacteroidetes	Cytophagia	Rhodonellum psychrophilum
tr|A0A017SZP8|A0A017SZP8_9DELT	1	Proteobacteria	Deltaproteobacteria	Chondromyces apiculatus
tr|R5WR07|R5WR07_9BACT	1	Bacteroidetes	Bacteroidia	Alistipes sp. CAG:157
tr|F0SQW1|F0SQW1_PLABD	1	Planctomycetes	Planctomycetia	Planctomyces brasiliensis
tr|A0A034UBF2|A0A034UBF2_9NOCA	1	Actinobacteria	Actinobacteria	Nocardia brasiliensis
tr|L8DDJ3|L8DDJ3_9NOCA	1	Actinobacteria	Actinobacteria	Rhodococcus sp. AW25M09
tr|L7LDF5|L7LDF5_9ACTO	1	Actinobacteria	Actinobacteria	Gordonia hirsuta
tr|L7LBL5|L7LBL5_9ACTO	1	Actinobacteria	Actinobacteria	Gordonia hirsuta
tr|H0QWP2|H0QWP2_9ACTO	1	Actinobacteria	Actinobacteria	Gordonia effusa
tr|H0RLY8|H0RLY8_9ACTO	1	Actinobacteria	Actinobacteria	Gordonia polyisoprenivorans
tr|H0R4X5|H0R4X5_9ACTO	1	Actinobacteria	Actinobacteria	Gordonia effusa
tr|W5TH26|W5TH26_9NOCA	1	Actinobacteria	Actinobacteria	Nocardia nova
tr|R4UX48|R4UX48_MYCAB	1	Actinobacteria	Actinobacteria	Mycobacterium abscessus
tr|G8RXW8|G8RXW8_MYCRN	1	Actinobacteria	Actinobacteria	Mycobacterium rhodesiae
tr|E5XTR0|E5XTR0_9ACTO	1	Actinobacteria	Actinobacteria	Segniliparus rugosus
tr|U5E6V1|U5E6V1_NOCAS	1	Actinobacteria	Actinobacteria	Nocardia asteroides
tr|F6ERP1|F6ERP1_AMYSD	1	Actinobacteria	Actinobacteria	Amycolicicoccus subflavus
tr|J9SRZ1|J9SRZ1_9ACTO	1	Actinobacteria	Actinobacteria	Gordonia sp. KTR9
tr|D5UW66|D5UW66_TSUPD	6	Actinobacteria	Actinobacteria	Tsukamurella paurometabola
tr|A0A0C1DBV2|A0A0C1DBV2_9NOCA	1	Actinobacteria	Actinobacteria	Nocardia vulneris
tr|E5XS62|E5XS62_9ACTO	1	Actinobacteria	Actinobacteria	Segniliparus rugosus
tr|H0R405|H0R405_9ACTO	1	Actinobacteria	Actinobacteria	Gordonia effusa
tr|H6R4F1|H6R4F1_NOCCG	1	Actinobacteria	Actinobacteria	Nocardia cyriacigeorgica
tr|W5TK72|W5TK72_9NOCA	1	Actinobacteria	Actinobacteria	Nocardia nova
tr|H5UG92|H5UG92_9ACTO	1	Actinobacteria	Actinobacteria	Gordonia terrae
tr|I0P732|I0P732_MYCAB	1	Actinobacteria	Actinobacteria	Mycobacterium abscessus
tr|H0QWG0|H0QWG0_9ACTO	1	Actinobacteria	Actinobacteria	Gordonia effusa

tr|G7GQQ7|G7GQQ7_9ACTO	1	Actinobacteria	Actinobacteria	Gordonia amarae
tr|Q5YVH0|Q5YVH0_NOCFA	1	Actinobacteria	Actinobacteria	Nocardia farcinica
tr|U5EI33|U5EI33_NOCAS	1	Actinobacteria	Actinobacteria	Nocardia asteroides
tr|G7H7G0|G7H7G0_9ACTO	1	Actinobacteria	Actinobacteria	Gordonia araii
tr|D5UP34|D5UP34_TSUPD	1	Actinobacteria	Actinobacteria	Tsukamurella paurometabola
tr|L7LEA8|L7LEA8_9ACTO	1	Actinobacteria	Actinobacteria	Gordonia hirsuta
tr|G7GSP9|G7GSP9_9ACTO	1	Actinobacteria	Actinobacteria	Gordonia amarae
tr|X5LBT5|X5LBT5_9MYCO	1	Actinobacteria	Actinobacteria	Mycobacterium vulneris
tr|L8DAF2|L8DAF2_9NOCA	2	Actinobacteria	Actinobacteria	Rhodococcus sp. AW25M09
tr|H0ITI5|H0ITI5_MYCAB	1	Actinobacteria	Actinobacteria	Mycobacterium abscessus
tr|A0A0B8NLH2|A0A0B8NLH2_9NOCA	1	Actinobacteria	Actinobacteria	Nocardia seriolae
tr|D5UYR8|D5UYR8_TSUPD	1	Actinobacteria	Actinobacteria	Tsukamurella paurometabola
tr|H0RG54|H0RG54_9ACTO	1	Actinobacteria	Actinobacteria	Gordonia polyisoprenivorans
tr|K6XAK4|K6XAK4_9ACTO	1	Actinobacteria	Actinobacteria	Gordonia namibiensis
tr|A0A0A1DKY3|A0A0A1DKY3_NOCSI	2	Actinobacteria	Actinobacteria	Pimelobacter simplex
tr|W7S8N6|W7S8N6_9PSEU	2	Actinobacteria	Actinobacteria	Kutzneria sp. 744
tr|G4HXY5|G4HXY5_MYCRH	1	Actinobacteria	Actinobacteria	Mycobacterium rhodesiae
tr|W5WJZ9|W5WJZ9_9PSEU	1	Actinobacteria	Actinobacteria	Kutzneria albida
tr|Q0SD31|Q0SD31_RHOJR	1	Actinobacteria	Actinobacteria	Rhodococcus jostii
tr|H0R662|H0R662_9ACTO	1	Actinobacteria	Actinobacteria	Gordonia effusa
tr|R1I6Y7|R1I6Y7_9PSEU	1	Actinobacteria	Actinobacteria	Amycolatopsis vancoresmycina
tr|K6WXK8|K6WXK8_9ACTO	1	Actinobacteria	Actinobacteria	Gordonia namibiensis
tr|N1M1R1|N1M1R1_9NOCA	1	Actinobacteria	Actinobacteria	Rhodococcus sp. EsD8
tr|D3QB63|D3QB63_STANL	1	Actinobacteria	Actinobacteria	Stackebrandtia nassauensis
tr|W5TKJ6|W5TKJ6_9NOCA	1	Actinobacteria	Actinobacteria	Nocardia nova
tr|Q82B08|Q82B08_STRAW	1	Actinobacteria	Actinobacteria	Streptomyces avermitilis
tr|E9UWP4|E9UWP4_9ACTO	1	Actinobacteria	Actinobacteria	Nocardioidaceae bacterium Broad-1

tr|X8AC78|X8AC78_MYCAV	6	Actinobacteria	Actinobacteria	Mycobacterium avium
tr|A0A049DVU2|A0A049DVU2_MYCAV	1	Actinobacteria	Actinobacteria	Mycobacterium avium
tr|A0A076N4G4|A0A076N4G4_AMYME	2	Actinobacteria	Actinobacteria	Amycolatopsis methanolica
tr|E2SCQ7|E2SCQ7_9ACTO	1	Actinobacteria	Actinobacteria	Aeromicrobium marinum
tr|W5TSW7|W5TSW7_9NOCA	2	Actinobacteria	Actinobacteria	Nocardia nova
tr|L7KJL2|L7KJL2_9ACTO	1	Actinobacteria	Actinobacteria	Gordonia aichiensis
tr|X8C805|X8C805_MYCXE	1	Actinobacteria	Actinobacteria	Mycobacterium xenopi
tr|E6S9N7|E6S9N7_INTC7	2	Actinobacteria	Actinobacteria	Intrasporangium calvum
tr|U5EJN4|U5EJN4_NOCAS	1	Actinobacteria	Actinobacteria	Nocardia asteroides
tr|H6RMU9|H6RMU9_BLASD	2	Actinobacteria	Actinobacteria	Blastococcus saxobsidens
tr|A0A076MUX5|A0A076MUX5_AMYME	2	Actinobacteria	Actinobacteria	Amycolatopsis methanolica
tr|N1MJ19|N1MJ19_9NOCA	1	Actinobacteria	Actinobacteria	Rhodococcus sp. EsD8
tr|C1B3R9|C1B3R9_RHOOB	2	Actinobacteria	Actinobacteria	Rhodococcus opacus
tr|L7L8J5|L7L8J5_9ACTO	1	Actinobacteria	Actinobacteria	Gordonia hirsuta
tr|G7GKG5|G7GKG5_9ACTO	1	Actinobacteria	Actinobacteria	Gordonia amarae
tr|H0R5E0|H0R5E0_9ACTO	2	Actinobacteria	Actinobacteria	Gordonia effusa
tr|E2S873|E2S873_9ACTO	1	Actinobacteria	Actinobacteria	Aeromicrobium marinum
tr|G7GSR4|G7GSR4_9ACTO	1	Actinobacteria	Actinobacteria	Gordonia amarae
tr|F8B412|F8B412_FRADG	2	Actinobacteria	Actinobacteria	Frankia symbiont of Datisca glomerata
tr|E9SXF7|E9SXF7_RHOHA	1	Actinobacteria	Actinobacteria	Rhodococcus equi
tr|H5TSA9|H5TSA9_9ACTO	1	Actinobacteria	Actinobacteria	Gordonia otitidis
tr|C6WH36|C6WH36_ACTMD	1	Actinobacteria	Actinobacteria	Actinosynnema mirum
tr|D9UW72|D9UW72_9ACTO	1	Actinobacteria	Actinobacteria	Streptomyces sp. AA4
tr|H5X5Q7|H5X5Q7_9PSEU	1	Actinobacteria	Actinobacteria	Saccharomonospora marina
tr|G7GP49|G7GP49_9ACTO	1	Actinobacteria	Actinobacteria	Gordonia amarae
tr|E6JBI1|E6JBI1_9ACTO	1	Actinobacteria	Actinobacteria	Dietzia cinnamea
tr|A0A0B2BNA9|A0A0B2BNA9_9ACTO	1	Actinobacteria	Actinobacteria	Mumia flava

tr|X7ZQS1|X7ZQS1_MYCKA	1	Actinobacteria	Actinobacteria	Mycobacterium kansasii
tr|W8HIN5|W8HIN5_RHOOP	2	Actinobacteria	Actinobacteria	Rhodococcus opacus
tr|E6JDS7|E6JDS7_9ACTO	2	Actinobacteria	Actinobacteria	Dietzia cinnamea
tr|T1VIH4|T1VIH4_RHOER	1	Actinobacteria	Actinobacteria	Rhodococcus erythropolis
tr|D1AD48|D1AD48_THECD	1	Actinobacteria	Actinobacteria	Thermomonospora curvata
tr|A1SKI9|A1SKI9_NOCSJ	1	Actinobacteria	Actinobacteria	Nocardioides sp. JS614
tr|B5H0E3|B5H0E3_STRC2	1	Actinobacteria	Actinobacteria	Streptomyces clavuligerus
tr|A0A0A1DIP0|A0A0A1DIP0_NOCSI	1	Actinobacteria	Actinobacteria	Pimelobacter simplex
tr|L7LGC5|L7LGC5_9ACTO	1	Actinobacteria	Actinobacteria	Gordonia sihwensis
tr|H5XA18|H5XA18_9PSEU	2	Actinobacteria	Actinobacteria	Saccharomonospora marina
tr|D1A3I8|D1A3I8_THECD	1	Actinobacteria	Actinobacteria	Thermomonospora curvata
tr|A0A0A1DK44|A0A0A1DK44_NOCSI	1	Actinobacteria	Actinobacteria	Pimelobacter simplex
tr|E2SCQ6|E2SCQ6_9ACTO	1	Actinobacteria	Actinobacteria	Aeromicrobium marinum
tr|G7GSR5|G7GSR5_9ACTO	1	Actinobacteria	Actinobacteria	Gordonia amarae
tr|M2VIV3|M2VIV3_9NOCA	1	Actinobacteria	Actinobacteria	Rhodococcus triatomae
tr|D5P4I7|D5P4I7_9MYCO	1	Actinobacteria	Actinobacteria	Mycobacterium parascrofulaceum
tr|X7Z6I4|X7Z6I4_MYCKA	1	Actinobacteria	Actinobacteria	Mycobacterium kansasii
tr|E1QEH6|E1QEH6_DESB2	1	Proteobacteria	Deltaproteobacteria	Desulfarculus baarsii
tr|A0A091F962|A0A091F962_9DELT	1	Proteobacteria	Deltaproteobacteria	Smithella sp. SCADC
tr|G4FMK9|G4FMK9_9SYNE	1	Cyanobacteria		Synechococcus sp. WH 8016
tr|B5IQJ6|B5IQJ6_9CHRO	1	Cyanobacteria		Cyanobium sp. PCC 7001
tr|A2C0C3|A2C0C3_PROM1	1	Cyanobacteria		Prochlorococcus marinus
tr|A0A0A2AM06|A0A0A2AM06_PROMR	1	Cyanobacteria		Prochlorococcus marinus
tr|A0A0A2BW26|A0A0A2BW26_9PROC	1	Cyanobacteria		Prochlorococcus sp. MIT 0602
tr|A5GQG4|A5GQG4_SYNR3	1	Cyanobacteria		Synechococcus sp. RCC307
tr|Q5N2W8|Q5N2W8_SYNP6	1	Cyanobacteria		Synechococcus elongatus
tr|K9SH59|K9SH59_9CYAN	1	Cyanobacteria		Pseudanabaena sp. PCC 7367

tr|Q2JUB0|Q2JUB0_SYNJA	1	Cyanobacteria		Synechococcus sp. JA-3-3Ab
tr|B4WJN6|B4WJN6_9SYNE	1	Cyanobacteria		Synechococcus sp. PCC 7335
tr|U5DIK0|U5DIK0_9CHRO	1	Cyanobacteria		Rubidibacter lacunae
tr|K9Y9N2|K9Y9N2_HALP7	1	Cyanobacteria		Halothece sp. PCC 7418
tr|K9T3H1|K9T3H1_9CYAN	1	Cyanobacteria		Pleurocapsa minor
tr|K9XQV7|K9XQV7_STAC7	1	Cyanobacteria		Stanieria cyanosphaera
tr|K9W8T4|K9W8T4_9CYAN	1	Cyanobacteria		Microcoleus sp. PCC 7113
tr|B7KB83|B7KB83_CYAP7	1	Cyanobacteria		Cyanothece sp. PCC 7424
tr|K9RWX8|K9RWX8_SYNP3	1	Cyanobacteria		Synechococcus sp. PCC 6312
tr|L8MSA2|L8MSA2_9CYAN	1	Cyanobacteria		Pseudanabaena biceps
tr|B0C226|B0C226_ACAM1	1	Cyanobacteria		Acaryochloris marina
tr|B8HTP7|B8HTP7_CYAP4	1	Cyanobacteria		Cyanothece sp. PCC 7425
tr|A0A077JDZ5|A0A077JDZ5_9CYAN	1	Cyanobacteria		cyanobacterium endosymbiont of Epithemia turgida
tr|H1WM06|H1WM06_9CYAN	1	Cyanobacteria		Arthrospira sp. PCC 8005
tr|K8GQV7|K8GQV7_9CYAN	1	Cyanobacteria		Oscillatoriales cyanobacterium JSC-12
tr|U9VTS0|U9VTS0_9CYAN	1	Cyanobacteria		Leptolyngbya sp. Heron Island J
tr|A0A0C1TAB8|A0A0C1TAB8_9CHRO	1	Cyanobacteria		Aphanocapsa montana
tr|D1A3I5|D1A3I5_THECD	1	Actinobacteria	Actinobacteria	Thermomonospora curvata
tr|D1AD45|D1AD45_THECD	1	Actinobacteria	Actinobacteria	Thermomonospora curvata
tr|W5TQP4|W5TQP4_9NOCA	1	Actinobacteria	Actinobacteria	Nocardia nova
tr|F6ERN9|F6ERN9_AMYSD	1	Actinobacteria	Actinobacteria	Amycolicicoccus subflavus
tr|G7GSQ1|G7GSQ1_9ACTO	1	Actinobacteria	Actinobacteria	Gordonia amarae
tr|A0A0C1DRH0|A0A0C1DRH0_9NOCA	1	Actinobacteria	Actinobacteria	Nocardia vulneris
tr|I0P730|I0P730_MYCAB	1	Actinobacteria	Actinobacteria	Mycobacterium abscessus
tr|L7LB86|L7LB86_9ACTO	1	Actinobacteria	Actinobacteria	Gordonia hirsuta
tr|H0R403|H0R403_9ACTO	1	Actinobacteria	Actinobacteria	Gordonia effusa
tr|G7GY04|G7GY04_9ACTO	1	Actinobacteria	Actinobacteria	Gordonia araii

tr|J9SBI8|J9SBI8_9ACTO	1	Actinobacteria	Actinobacteria	Gordonia sp. KTR9
tr|A0A0B8NCI5|A0A0B8NCI5_9NOCA	1	Actinobacteria	Actinobacteria	Nocardia seriolae
tr|N1M7R1|N1M7R1_9NOCA	1	Actinobacteria	Actinobacteria	Rhodococcus sp. EsD8
tr|H0R5M6|H0R5M6_9ACTO	1	Actinobacteria	Actinobacteria	Gordonia effusa
tr|H0QYS0|H0QYS0_9ACTO	1	Actinobacteria	Actinobacteria	Gordonia effusa
tr|G7GQQ5|G7GQQ5_9ACTO	1	Actinobacteria	Actinobacteria	Gordonia amarae
tr|A0A024LYA0|A0A024LYA0_9MYCO	1	Actinobacteria	Actinobacteria	Mycobacterium farcinogenes
tr|F5YRK0|F5YRK0_MYCSD	1	Actinobacteria	Actinobacteria	Mycobacterium sp. JDM601
tr|E5XS64|E5XS64_9ACTO	1	Actinobacteria	Actinobacteria	Segniliparus rugosus
tr|G7H7G2|G7H7G2_9ACTO	1	Actinobacteria	Actinobacteria	Gordonia araii
tr|H0R4X3|H0R4X3_9ACTO	1	Actinobacteria	Actinobacteria	Gordonia effusa
tr|W5WKH5|W5WKH5_9PSEU	1	Actinobacteria	Actinobacteria	Kutzneria albida
tr|A1SKJ0|A1SKJ0_NOCSJ	1	Actinobacteria	Actinobacteria	Nocardioides sp. JS614
tr|D6AF89|D6AF89_STRFL	1	Actinobacteria	Actinobacteria	Streptomyces filamentosus
tr|M2XNH7|M2XNH7_9NOCA	1	Actinobacteria	Actinobacteria	Rhodococcus qingshengii
tr|E6JDS8|E6JDS8_9ACTO	1	Actinobacteria	Actinobacteria	Dietzia cinnamea
tr|D5UQ86|D5UQ86_TSUPD	1	Actinobacteria	Actinobacteria	Tsukamurella paurometabola
tr|E2SEJ9|E2SEJ9_9ACTO	1	Actinobacteria	Actinobacteria	Aeromicrobium marinum
tr|H0QWP3|H0QWP3_9ACTO	1	Actinobacteria	Actinobacteria	Gordonia effusa
tr|I2AD27|I2AD27_9MYCO	2	Actinobacteria	Actinobacteria	Mycobacterium sp. MOTT36Y
tr|L7LAW1|L7LAW1_9ACTO	1	Actinobacteria	Actinobacteria	Gordonia hirsuta
tr|F4CXJ6|F4CXJ6_PSEUX	1	Actinobacteria	Actinobacteria	Pseudonocardia dioxanivorans
tr|K6WGW0|K6WGW0_9ACTO	1	Actinobacteria	Actinobacteria	Gordonia namibiensis
tr|A0A098BQD0|A0A098BQD0_9NOCA	1	Actinobacteria	Actinobacteria	Rhodococcus ruber
tr|L7LDI1|L7LDI1_9ACTO	1	Actinobacteria	Actinobacteria	Gordonia hirsuta
tr|H0RK29|H0RK29_9ACTO	1	Actinobacteria	Actinobacteria	Gordonia polyisoprenivorans
tr|L7L8H2|L7L8H2_9ACTO	1	Actinobacteria	Actinobacteria	Gordonia hirsuta

tr|F8B411|F8B411_FRADG	1	Actinobacteria	Actinobacteria	Frankia symbiont of Datisca glomerata
tr|W4A9M2|W4A9M2_RHORH	2	Actinobacteria	Actinobacteria	Rhodococcus rhodochrous
tr|W9DNC4|W9DNC4_9PSEU	1	Actinobacteria	Actinobacteria	Amycolatopsis halophila
tr|M2XC84|M2XC84_9NOCA	1	Actinobacteria	Actinobacteria	Rhodococcus triatomae
tr|A0A0A1DT20|A0A0A1DT20_NOCSI	1	Actinobacteria	Actinobacteria	Pimelobacter simplex
tr|D2B9W1|D2B9W1_STRRD	2	Actinobacteria	Actinobacteria	Streptosporangium roseum
tr|E5XTQ9|E5XTQ9_9ACTO	1	Actinobacteria	Actinobacteria	Segniliparus rugosus
tr|A0A0C1DA60|A0A0C1DA60_9NOCA	1	Actinobacteria	Actinobacteria	Nocardia vulneris
tr|K0P2I7|K0P2I7_9BACT	1	Bacteroidetes	Cytophagia	Cardinium endosymbiont of Encarsia pergandiella
tr|B3ES33|B3ES33_AMOA5	1	Bacteroidetes	Cytophagia	Candidatus Amoebophilus asiaticus
tr|I4AGD9|I4AGD9_FLELS	1	Bacteroidetes	Cytophagia	Flexibacter litoralis
tr|M7NKI7|M7NKI7_9BACT	1	Bacteroidetes	Cytophagia	Cesiribacter andamanensis
tr|A0A098L870|A0A098L870_9BACT	1	Bacteroidetes	Cytophagia	Sporocytophaga myxococcoides
tr|F8EK33|F8EK33_RUNSL	1	Bacteroidetes	Cytophagia	Runella slithyformis
tr|J1FGV0|J1FGV0_9BACT	1	Bacteroidetes	Cytophagia	Pontibacter sp. BAB1700
tr|A0A081PD39|A0A081PD39_9SPHI	1	Bacteroidetes	Sphingobacteriia	Sphingobacterium antarcticum
tr|A1ZPY6|A1ZPY6_9BACT	1	Bacteroidetes	Cytophagia	Microscilla marina
tr|E4TVH0|E4TVH0_MARTH	1	Bacteroidetes	Cytophagia	Marivirga tractuosa
tr|I0KE54|I0KE54_9BACT	1	Bacteroidetes	Cytophagia	Fibrella aestuarina
tr|R7HMZ6|R7HMZ6_9BACT	1	Bacteroidetes	Bacteroidia	Prevotella sp. CAG:279
tr|T2NDF6|T2NDF6_PORGN	1	Bacteroidetes	Bacteroidia	Porphyromonas gingivalis
tr|I2EQY5|I2EQY5_EMTOG	1	Bacteroidetes	Cytophagia	Emticicia oligotrophica
tr|A0A099WU86|A0A099WU86_PORCN	1	Bacteroidetes	Bacteroidia	Porphyromonas cangingivalis
tr|R5AQU5|R5AQU5_9BACT	1	Bacteroidetes	Bacteroidia	Prevotella sp. CAG:1031
tr|G5HA61|G5HA61_9BACT	1	Bacteroidetes	Bacteroidia	Alistipes indistinctus
tr|G8R7C0|G8R7C0_OWEHD	1	Bacteroidetes	Flavobacteriia	Owenweeksia hongkongensis
tr|C7PH47|C7PH47_CHIPD	1	Bacteroidetes	Sphingobacteriia	Chitinophaga pinensis

tr|A0A0A2EHY2|A0A0A2EHY2_9PORP	1	Bacteroidetes	Bacteroidia	Porphyromonadaceae bacterium COT-184 OH4590
tr|F5IW51|F5IW51_9PORP	1	Bacteroidetes	Bacteroidia	Dysgonomonas gadei
tr|F2KZG4|F2KZG4_PREDF	1	Bacteroidetes	Bacteroidia	Prevotella denticola
tr|Q11X28|Q11X28_CYTH3	1	Bacteroidetes	Cytophagia	Cytophaga hutchinsonii
tr|A0A090VSQ0|A0A090VSQ0_9FLAO	1	Bacteroidetes	Flavobacteriia	Jejuia pallidilutea
tr|A0A0A2E7S6|A0A0A2E7S6_9PORP	1	Bacteroidetes	Bacteroidia	Porphyromonas macacae
tr|A0A0C1XY96|A0A0C1XY96_9CYAN	1	Cyanobacteria		Hassallia byssoidea
tr|L1MNU8|L1MNU8_9BACT	1	Bacteroidetes	Bacteroidia	Prevotella sp. oral taxon 473
tr|S8GQX7|S8GQX7_9BACT	1	Bacteroidetes		Bacteroidetes bacterium oral taxon 272
tr|G9S299|G9S299_9PORP	1	Bacteroidetes	Bacteroidia	Tannerella sp. 6_1_58FAA_CT1
tr|H1H6W3|H1H6W3_9FLAO	1	Bacteroidetes	Flavobacteriia	Myroides odoratimimus
tr|R5P0D2|R5P0D2_9PORP	1	Bacteroidetes	Bacteroidia	Odoribacter sp. CAG:788
tr|F8N780|F8N780_9BACT	1	Bacteroidetes	Bacteroidia	Prevotella multisaccharivorax
tr|A0A0A2N285|A0A0A2N285_9FLAO	1	Bacteroidetes	Flavobacteriia	Flavobacterium subsaxonicum
tr|X5DME2|X5DME2_9BACT	1	Bacteroidetes	Bacteroidia	Draconibacterium orientale
tr|A0A069ZPF2|A0A069ZPF2_9PORP	1	Bacteroidetes	Bacteroidia	Porphyromonas sp. KLE 1280
tr|A0A0A2FVJ7|A0A0A2FVJ7_9PORP	1	Bacteroidetes	Bacteroidia	Porphyromonas sp. COT-108 OH2963
tr|A0A0A2DUW2|A0A0A2DUW2_9PORP	1	Bacteroidetes	Bacteroidia	Porphyromonas sp. COT-239 OH1446
tr|R5GCB1|R5GCB1_9PORP	1	Bacteroidetes	Bacteroidia	Porphyromonas sp. CAG:1061
tr|I4A1W8|I4A1W8_ORNRL	1	Bacteroidetes	Flavobacteriia	Ornithobacterium rhinotracheale
tr|A0A0A2F3F9|A0A0A2F3F9_9PORP	1	Bacteroidetes	Bacteroidia	Porphyromonas sp. COT-290 OH860
tr|H8KQS8|H8KQS8_SOLCM	1	Bacteroidetes	Sphingobacteriia	Solitalea canadensis
tr|C0BK49|C0BK49_FLABM	1	Bacteroidetes	Flavobacteriia	Flavobacteria bacterium MS024-2A
tr|H5SGK0|H5SGK0_9BACT	1	Bacteroidetes		uncultured Bacteroidetes bacterium
tr|R7EV28|R7EV28_9BACT	1	Bacteroidetes	Bacteroidia	Prevotella sp. CAG:485
tr|F0NYE7|F0NYE7_WEEVC	1	Bacteroidetes	Flavobacteriia	Weeksella virosa
tr|A0A0C1IWJ7|A0A0C1IWJ7_9SPHI	1	Bacteroidetes	Sphingobacteriia	Flavihumibacter solisilvae

tr|A0A085L335|A0A085L335_9FLAO	1	Bacteroidetes	Flavobacteriia	Schleiferia thermophila
tr|I3YYY0|I3YYY0_AEQSU	1	Bacteroidetes	Flavobacteriia	Aequorivita sublithincola
tr|G8TKE0|G8TKE0_NIAKG	1	Bacteroidetes	Sphingobacteriia	Niastella koreensis
tr|A0A076HT00|A0A076HT00_9BACT	1	Bacteroidetes	Cytophagia	Hymenobacter sp. APR13
tr|R5NYV3|R5NYV3_9BACT	1	Bacteroidetes	Bacteroidia	Paraprevotella clara CAG:116
tr|T0L699|T0L699_9BACT	1			candidate division Zixibacteria
tr|A0A060RBB0|A0A060RBB0_9BACT	1	Bacteroidetes	Bacteroidia	Mucinivorans hirudinis
tr|A0A0B7IDH7|A0A0B7IDH7_9FLAO	1	Bacteroidetes	Flavobacteriia	Capnocytophaga canimorsus
tr|A0A084THK3|A0A084THK3_9FLAO	1	Bacteroidetes	Flavobacteriia	Mangrovimonas yunxiaonensis
tr|I0W9H7|I0W9H7_9FLAO	1	Bacteroidetes	Flavobacteriia	Imtechella halotolerans
tr|H8XQU0|H8XQU0_FLAIG	1	Bacteroidetes	Flavobacteriia	Flavobacterium indicum
tr|C3J8P0|C3J8P0_9PORP	1	Bacteroidetes	Bacteroidia	Porphyromonas endodontalis
tr|K2PTK0|K2PTK0_9FLAO	1	Bacteroidetes	Flavobacteriia	Galbibacter marinus
tr|C6WZS1|C6WZS1_FLAB3	1	Bacteroidetes	Flavobacteriia	Flavobacteriaceae bacterium 3519-10
tr|A9DWR2|A9DWR2_9FLAO	1	Bacteroidetes	Flavobacteriia	Kordia algicida
tr|S2W8N2|S2W8N2_9FLAO	1	Bacteroidetes	Flavobacteriia	Capnocytophaga granulosa
tr|W8VXU7|W8VXU7_9FLAO	1	Bacteroidetes	Flavobacteriia	Nonlabens marinus
tr|A0A098RZV5|A0A098RZV5_9SPHI	1	Bacteroidetes	Sphingobacteriia	Phaeodactylibacter xiamenensis
tr|F2IBN3|F2IBN3_FLUTR	1	Bacteroidetes	Flavobacteriia	Fluviicola taffensis
tr|F4KKC0|F4KKC0_PORAD	1	Bacteroidetes	Bacteroidia	Porphyromonas asaccharolytica
tr|W4PGU6|W4PGU6_9BACE	1	Bacteroidetes	Bacteroidia	Bacteroides pyogenes
tr|A0M1W6|A0M1W6_GRAFK	1	Bacteroidetes	Flavobacteriia	Gramella forsetii
tr|A0A0A2GBH2|A0A0A2GBH2_9PORP	1	Bacteroidetes	Bacteroidia	Porphyromonas gingivicanis
tr|D0MEJ5|D0MEJ5_RHOM4	1	Bacteroidetes		Rhodothermus marinus
tr|W7YFG6|W7YFG6_9BACT	1	Bacteroidetes	Bacteroidia	Saccharicrinis fermentans
tr|H1XUM8|H1XUM8_9BACT	1			Caldithrix abyssi
tr|W0F9G9|W0F9G9_9SPHI	1	Bacteroidetes	Sphingobacteriia	Niabella soli

tr|A0A099Y3B0|A0A099Y3B0_9FLAO	1	Bacteroidetes	Flavobacteriia	Polaribacter sp. Hel1_85
tr|K1Y202|K1Y202_9BACT	1			uncultured bacterium
tr|R5HZE0|R5HZE0_9BACT	1	Bacteroidetes	Bacteroidia	Alistipes sp. CAG:831
tr|R5SG86|R5SG86_9BACE	1	Bacteroidetes	Bacteroidia	Bacteroides sp. CAG:545
tr|B0VIY3|B0VIY3_CLOAI	1	Cloacimonetes		Candidatus Cloacimonas acidaminovorans
tr|C9RPE4|C9RPE4_FIBSS	1	Fibrobacteres	Fibrobacteria	Fibrobacter succinogenes
tr|J1I4E7|J1I4E7_9SPHI	1	Bacteroidetes	Sphingobacteriia	Saprospira grandis
tr|F4KW20|F4KW20_HALH1	1	Bacteroidetes	Sphingobacteriia	Haliscomenobacter hydrossis
tr|D5HC85|D5HC85_SALRM	1	Bacteroidetes		Salinibacter ruber
tr|A0A095YWT5|A0A095YWT5_9FIRM	1	Firmicutes	Clostridia	Clostridiales bacterium S5-A11
tr|W0RMM3|W0RMM3_9BACT	1	Gemmatimonadetes	Gemmatimonadetes	Gemmatimonadetes bacterium KBS708
tr|A0A0A6QVG1|A0A0A6QVG1_9GAMM	1	Proteobacteria	Gammaproteobacteria	Methylomonas denitrificans
tr|J3GHZ5|J3GHZ5_9PSED	1	Proteobacteria	Gammaproteobacteria	Pseudomonas sp. GM55
tr|F2LVU5|F2LVU5_HIPMA	1	Proteobacteria	Deltaproteobacteria	Hippea maritima
tr|F2NDZ2|F2NDZ2_DESAR	1	Proteobacteria	Deltaproteobacteria	Desulfobacca acetoxidans
tr|W0SFH4|W0SFH4_9RHOO	1	Proteobacteria	Betaproteobacteria	Sulfuritalea hydrogenivorans
tr|I4C465|I4C465_DESTA	1	Proteobacteria	Deltaproteobacteria	Desulfomonile tiedjei
tr|A0A080QB06|A0A080QB06_9GAMM	1	Proteobacteria	Gammaproteobacteria	Francisella philomiragia
tr|E1YET2|E1YET2_9DELT	1	Proteobacteria	Deltaproteobacteria	uncultured Desulfobacterium sp.
tr|A8ZWW9|A8ZWW9_DESOH	1	Proteobacteria	Deltaproteobacteria	Desulfococcus oleovorans
tr|A0A017TF38|A0A017TF38_9DELT	1	Proteobacteria	Deltaproteobacteria	Chondromyces apiculatus
tr|E2SEJ8|E2SEJ8_9ACTO	1	Actinobacteria	Actinobacteria	Aeromicrobium marinum
tr|F6EN09|F6EN09_AMYSD	1	Actinobacteria	Actinobacteria	Amycolicicoccus subflavus
tr|E9UWC6|E9UWC6_9ACTO	2	Actinobacteria	Actinobacteria	Nocardioidaceae bacterium Broad-1
tr|K5B7D8|K5B7D8_9MYCO	1	Actinobacteria	Actinobacteria	Mycobacterium hassiacum
tr|U0EGC3|U0EGC3_9NOCA	1	Actinobacteria	Actinobacteria	Rhodococcus sp. P27
tr|A0A0A1DLZ1|A0A0A1DLZ1_NOCSI	1	Actinobacteria	Actinobacteria	Pimelobacter simplex

tr|D2B9W0|D2B9W0_STRRD	1	Actinobacteria	Actinobacteria	Streptosporangium roseum
tr|X7XVT8|X7XVT8_MYCKA	2	Actinobacteria	Actinobacteria	Mycobacterium kansasii
tr|F6EHT2|F6EHT2_AMYSD	1	Actinobacteria	Actinobacteria	Amycolicicoccus subflavus
tr|G7GUZ5|G7GUZ5_9ACTO	1	Actinobacteria	Actinobacteria	Gordonia amarae
tr|G7GMN9|G7GMN9_9ACTO	1	Actinobacteria	Actinobacteria	Gordonia amarae
tr|C6XKM3|C6XKM3_HIRBI	1	Proteobacteria	Alphaproteobacteria	Hirschia baltica
tr|A3VIP4|A3VIP4_9RHOB	1	Proteobacteria	Alphaproteobacteria	Maritimibacter alkaliphilus
tr|Q2RRK6|Q2RRK6_RHORT	1	Proteobacteria	Alphaproteobacteria	Rhodospirillum rubrum
tr|A0A0B8RJ17|A0A0B8RJ17_9PROT	1	Proteobacteria	Alphaproteobacteria	alpha proteobacterium U9-1i
tr|M2TAX0|M2TAX0_9PROT	1	Proteobacteria	Alphaproteobacteria	alpha proteobacterium JLT2015
tr|K9H4S2|K9H4S2_9PROT	1	Proteobacteria	Alphaproteobacteria	Caenispirillum salinarum
tr|A0A023D5F6|A0A023D5F6_ACIMT	1	Proteobacteria	Alphaproteobacteria	Acidomonas methanolica
tr|H6MXF2|H6MXF2_GORPV	1	Actinobacteria	Actinobacteria	Gordonia polyisoprenivorans
tr|I0P731|I0P731_MYCAB	1	Actinobacteria	Actinobacteria	Mycobacterium abscessus
tr|W5TL33|W5TL33_9NOCA	1	Actinobacteria	Actinobacteria	Nocardia nova
tr|H5U1P3|H5U1P3_9ACTO	1	Actinobacteria	Actinobacteria	Gordonia sputi
tr|W5TJN0|W5TJN0_9NOCA	1	Actinobacteria	Actinobacteria	Nocardia nova
tr|A0A0C1DEH2|A0A0C1DEH2_9NOCA	1	Actinobacteria	Actinobacteria	Nocardia vulneris
tr|W5THK6|W5THK6_9NOCA	1	Actinobacteria	Actinobacteria	Nocardia nova
tr|U5ECQ1|U5ECQ1_NOCAS	1	Actinobacteria	Actinobacteria	Nocardia asteroides
tr|K0ERB3|K0ERB3_9NOCA	1	Actinobacteria	Actinobacteria	Nocardia brasiliensis
tr|F6ERP0|F6ERP0_AMYSD	1	Actinobacteria	Actinobacteria	Amycolicicoccus subflavus
tr|D5UYR9|D5UYR9_TSUPD	1	Actinobacteria	Actinobacteria	Tsukamurella paurometabola
tr|E5XS63|E5XS63_9ACTO	1	Actinobacteria	Actinobacteria	Segniliparus rugosus
tr|L7KUZ4|L7KUZ4_9ACTO	1	Actinobacteria	Actinobacteria	Gordonia amicalis
tr|N1LYM3|N1LYM3_9NOCA	1	Actinobacteria	Actinobacteria	Rhodococcus sp. EsD8
tr|H0RG53|H0RG53_9ACTO	1	Actinobacteria	Actinobacteria	Gordonia polyisoprenivorans

tr|A0A0B8NBH4|A0A0B8NBH4_9NOCA	1	Actinobacteria	Actinobacteria	Nocardia seriolae
tr|L7LCD4|L7LCD4_9ACTO	1	Actinobacteria	Actinobacteria	Gordonia hirsuta
tr|L7KV49|L7KV49_9ACTO	1	Actinobacteria	Actinobacteria	Gordonia amicalis
tr|H0I724|H0I724_MYCAB	1	Actinobacteria	Actinobacteria	Mycobacterium abscessus
tr|H0ITI4|H0ITI4_MYCAB	1	Actinobacteria	Actinobacteria	Mycobacterium abscessus
tr|H0R404|H0R404_9ACTO	1	Actinobacteria	Actinobacteria	Gordonia effusa
tr|B4CTT9|B4CTT9_9BACT	1	Verrucomicrobia	Spartobacteria	Chthoniobacter flavus
tr|R6JAY2|R6JAY2_9BACT	1	Verrucomicrobia	Verrucomicrobiae	Akkermansia muciniphila CAG:154
tr|H0E5Y0|H0E5Y0_9ACTN	1	Actinobacteria	Actinobacteria	Patulibacter medicamentivorans
tr|D3F0X5|D3F0X5_CONWI	1	Actinobacteria	Actinobacteria	Conexibacter woesei
tr|H0E7I0|H0E7I0_9ACTN	1	Actinobacteria	Actinobacteria	Patulibacter medicamentivorans
tr|H0E9E6|H0E9E6_9ACTN	1	Actinobacteria	Actinobacteria	Patulibacter medicamentivorans
tr|H0E5I8|H0E5I8_9ACTN	1	Actinobacteria	Actinobacteria	Patulibacter medicamentivorans
tr|H0E614|H0E614_9ACTN	1	Actinobacteria	Actinobacteria	Patulibacter medicamentivorans
tr|H0E8D2|H0E8D2_9ACTN	1	Actinobacteria	Actinobacteria	Patulibacter medicamentivorans
tr|H0EAY3|H0EAY3_9ACTN	1	Actinobacteria	Actinobacteria	Patulibacter medicamentivorans
tr|A0A0B5F8Q2|A0A0B5F8Q2_9ACTO	1	Actinobacteria	Actinobacteria	Streptomyces albus
tr|H0E7N8|H0E7N8_9ACTN	1	Actinobacteria	Actinobacteria	Patulibacter medicamentivorans
tr|A0A076MQZ1|A0A076MQZ1_AMYME	2	Actinobacteria	Actinobacteria	Amycolatopsis methanolica
tr|E9USY6|E9USY6_9ACTO	1	Actinobacteria	Actinobacteria	Nocardioidaceae bacterium Broad-1
tr|H0E8D1|H0E8D1_9ACTN	1	Actinobacteria	Actinobacteria	Patulibacter medicamentivorans
tr|D3F0X4|D3F0X4_CONWI	1	Actinobacteria	Actinobacteria	Conexibacter woesei
tr|H0E7H6|H0E7H6_9ACTN	1	Actinobacteria	Actinobacteria	Patulibacter medicamentivorans
tr|H0E7N4|H0E7N4_9ACTN	6	Actinobacteria	Actinobacteria	Patulibacter medicamentivorans
tr|A0A076MKB1|A0A076MKB1_AMYME	1	Actinobacteria	Actinobacteria	Amycolatopsis methanolica
tr|F8AY50|F8AY50_FRADG	1	Actinobacteria	Actinobacteria	Frankia symbiont of Datisca glomerata
tr|H0E5K6|H0E5K6_9ACTN	1	Actinobacteria	Actinobacteria	Patulibacter medicamentivorans

tr|H0E5N8|H0E5N8_9ACTN	1	Actinobacteria	Actinobacteria	Patulibacter medicamentivorans
tr|H0E5I7|H0E5I7_9ACTN	1	Actinobacteria	Actinobacteria	Patulibacter medicamentivorans
tr|D3F0X8|D3F0X8_CONWI	1	Actinobacteria	Actinobacteria	Conexibacter woesei
tr|D3F0X9|D3F0X9_CONWI	2	Actinobacteria	Actinobacteria	Conexibacter woesei
tr|H0E5K4|H0E5K4_9ACTN	1	Actinobacteria	Actinobacteria	Patulibacter medicamentivorans
tr|H0E5N9|H0E5N9_9ACTN	1	Actinobacteria	Actinobacteria	Patulibacter medicamentivorans
tr|H0E7N5|H0E7N5_9ACTN	1	Actinobacteria	Actinobacteria	Patulibacter medicamentivorans
tr|H0EAM8|H0EAM8_9ACTN	1	Actinobacteria	Actinobacteria	Patulibacter medicamentivorans
tr|H0E6M8|H0E6M8_9ACTN	1	Actinobacteria	Actinobacteria	Patulibacter medicamentivorans
tr|S7HTJ1|S7HTJ1_9FIRM	6	Firmicutes	Negativicutes	Megasphaera sp. NM10
tr|G7GSQ2|G7GSQ2_9ACTO	1	Actinobacteria	Actinobacteria	Gordonia amarae
tr|F6ERN8|F6ERN8_AMYSD	1	Actinobacteria	Actinobacteria	Amycolicicoccus subflavus
tr|H6R8W7|H6R8W7_NOCCG	1	Actinobacteria	Actinobacteria	Nocardia cyriacigeorgica
tr|A0A0B8NB36|A0A0B8NB36_9NOCA	1	Actinobacteria	Actinobacteria	Nocardia seriolae
tr|W5TKU5|W5TKU5_9NOCA	1	Actinobacteria	Actinobacteria	Nocardia nova
tr|W5TMJ9|W5TMJ9_9NOCA	1	Actinobacteria	Actinobacteria	Nocardia nova
tr|I0P729|I0P729_MYCAB	1	Actinobacteria	Actinobacteria	Mycobacterium abscessus
tr|D5UW64|D5UW64_TSUPD	1	Actinobacteria	Actinobacteria	Tsukamurella paurometabola
tr|H0QWF7|H0QWF7_9ACTO	1	Actinobacteria	Actinobacteria	Gordonia effusa
tr|H0R402|H0R402_9ACTO	1	Actinobacteria	Actinobacteria	Gordonia effusa
tr|N1M6W3|N1M6W3_9NOCA	1	Actinobacteria	Actinobacteria	Rhodococcus sp. EsD8
tr|Q5YVH3|Q5YVH3_NOCFA	1	Actinobacteria	Actinobacteria	Nocardia farcinica
tr|H0RLY4|H0RLY4_9ACTO	1	Actinobacteria	Actinobacteria	Gordonia polyisoprenivorans
tr|H5UG95|H5UG95_9ACTO	1	Actinobacteria	Actinobacteria	Gordonia terrae
tr|X5LYS7|X5LYS7_9MYCO	1	Actinobacteria	Actinobacteria	Mycobacterium vulneris
tr|J9S7Z6|J9S7Z6_9ACTO	1	Actinobacteria	Actinobacteria	Gordonia sp. KTR9
tr|H0QWN9|H0QWN9_9ACTO	1	Actinobacteria	Actinobacteria	Gordonia effusa

tr|A0A0B8NIF8|A0A0B8NIF8_9NOCA	1	Actinobacteria	Actinobacteria	Nocardia seriolae
tr|H0RG51|H0RG51_9ACTO	1	Actinobacteria	Actinobacteria	Gordonia polyisoprenivorans
tr|J9S4Z5|J9S4Z5_9ACTO	1	Actinobacteria	Actinobacteria	Gordonia sp. KTR9
tr|L7LB07|L7LB07_9ACTO	1	Actinobacteria	Actinobacteria	Gordonia hirsuta
tr|M2X8Q5|M2X8Q5_9NOCA	1	Actinobacteria	Actinobacteria	Rhodococcus triatomae
tr|H0ITI2|H0ITI2_MYCAB	1	Actinobacteria	Actinobacteria	Mycobacterium abscessus
tr|L7KY97|L7KY97_9ACTO	1	Actinobacteria	Actinobacteria	Gordonia amicalis
tr|G7GY03|G7GY03_9ACTO	1	Actinobacteria	Actinobacteria	Gordonia araii
tr|A0A081GLL1|A0A081GLL1_9CHRO	1	Cyanobacteria		Cyanobium sp. CACIAM 14
tr|A3YTJ8|A3YTJ8_9SYNE	1	Cyanobacteria		Synechococcus sp. WH 5701
tr|A3ZAH4|A3ZAH4_9SYNE	1	Cyanobacteria		Synechococcus sp. RS9917
tr|G4FID8|G4FID8_9SYNE	1	Cyanobacteria		Synechococcus sp. WH 8016
tr|G0A3P5|G0A3P5_METMM	1	Proteobacteria	Gammaproteobacteria	Methylomonas methanica
tr|W4LUG8|W4LUG8_9DELT	1	Proteobacteria	Deltaproteobacteria	Candidatus Entotheonella sp. TSY1
tr|A5G4U4|A5G4U4_GEOUR	1	Proteobacteria	Deltaproteobacteria	Geobacter uraniireducens
tr|A0LKZ5|A0LKZ5_SYNFM	1	Proteobacteria	Deltaproteobacteria	Syntrophobacter fumaroxidans
tr|S7TVJ4|S7TVJ4_9DELT	1	Proteobacteria	Deltaproteobacteria	Desulfovibrio sp. X2
tr|D8F365|D8F365_9DELT	1	Proteobacteria	Deltaproteobacteria	delta proteobacterium NaphS2
tr|S7TW54|S7TW54_DESML	1	Proteobacteria	Deltaproteobacteria	Desulfococcus multivorans
tr|A0A0C2HS84|A0A0C2HS84_9DELT	1	Proteobacteria	Deltaproteobacteria	Geoalkalibacter ferrihydriticus
tr|F2NFS5|F2NFS5_DESAR	1	Proteobacteria	Deltaproteobacteria	Desulfobacca acetoxidans
tr|A0A0B5FKW7|A0A0B5FKW7_9DELT	1	Proteobacteria	Deltaproteobacteria	Geoalkalibacter subterraneus
tr|Q30ZW6|Q30ZW6_DESAG	1	Proteobacteria	Deltaproteobacteria	Desulfovibrio alaskensis
tr|B8DN57|B8DN57_DESVM	1	Proteobacteria	Deltaproteobacteria	Desulfovibrio vulgaris
tr|B2A0D5|B2A0D5_OPITP	1	Verrucomicrobia	Opitutae	Opitutus terrae
tr|F8UHI5|F8UHI5_9ZZZZ	1			uncultured microorganism
tr|H8Z4Y8|H8Z4Y8_9GAMM	1	Proteobacteria	Gammaproteobacteria	Thiorhodovibrio sp. 970

tr|W4MEV2|W4MEV2_9DELT	1	Proteobacteria	Deltaproteobacteria	Candidatus Entotheonella sp. TSY2
tr|L0GYF9|L0GYF9_9GAMM	1	Proteobacteria	Gammaproteobacteria	Thioflavicoccus mobilis
tr|B9XGL6|B9XGL6_PEDPL	1	Verrucomicrobia	Verrucomicrobiae	Pedosphaera parvula
tr|A0A0A2HXH7|A0A0A2HXH7_9DELT	1	Proteobacteria	Deltaproteobacteria	Desulfobulbus sp. Tol-SR
tr|B8KF18|B8KF18_9GAMM	1	Proteobacteria	Gammaproteobacteria	gamma proteobacterium NOR5-3
tr|B7RTG8|B7RTG8_9GAMM	1	Proteobacteria	Gammaproteobacteria	marine gamma proteobacterium HTCC2148
tr|E8RGR1|E8RGR1_DESPD	1	Proteobacteria	Deltaproteobacteria	Desulfobulbus propionicus
tr|G2FD41|G2FD41_9GAMM	1	Proteobacteria	Gammaproteobacteria	endosymbiont of Tevnia jerichonana
tr|H5WMD1|H5WMD1_9BURK	1	Proteobacteria	Betaproteobacteria	Burkholderiales bacterium JOSHI_001
tr|I3IC48|I3IC48_9GAMM	1	Proteobacteria	Gammaproteobacteria	Cellvibrio sp. BR
tr|V4JMR4|V4JMR4_9GAMM	1	Proteobacteria	Gammaproteobacteria	uncultured Thiohalocapsa sp. PB-PSB1
tr|K4KYM2|K4KYM2_SIMAS	1	Proteobacteria	Gammaproteobacteria	Simiduia agarivorans
tr|B2JLL5|B2JLL5_BURP8	1	Proteobacteria	Betaproteobacteria	Burkholderia phymatum
tr|W0E052|W0E052_MARPU	1	Proteobacteria	Gammaproteobacteria	Marichromatium purpuratum
tr|I4MRH9|I4MRH9_9BURK	1	Proteobacteria	Betaproteobacteria	Hydrogenophaga sp. PBC
tr|A0A081KAD9|A0A081KAD9_9GAMM	1	Proteobacteria	Gammaproteobacteria	Endozoicomonas elysicola
tr|R7L8K7|R7L8K7_9BACT	1	Verrucomicrobia	Opitutae	Coraliomargarita sp. CAG:312
tr|B8KVH9|B8KVH9_9GAMM	1	Proteobacteria	Gammaproteobacteria	Luminiphilus syltensis
tr|W9GXU5|W9GXU5_9PROT	1	Proteobacteria	Alphaproteobacteria	Skermanella stibiiresistens
tr|A0A095VVA6|A0A095VVA6_9GAMM	1	Proteobacteria	Gammaproteobacteria	Pseudohaliea rubra
tr|E1JSQ7|E1JSQ7_DESFR	1	Proteobacteria	Deltaproteobacteria	Desulfovibrio fructosivorans
tr|B8IZN6|B8IZN6_DESDA	1	Proteobacteria	Deltaproteobacteria	Desulfovibrio desulfuricans
tr|U2EPK4|U2EPK4_9GAMM	34	Proteobacteria	Gammaproteobacteria	Salinisphaera shabanensis
tr|A0A081N7I0|A0A081N7I0_9GAMM	1	Proteobacteria	Gammaproteobacteria	Endozoicomonas montiporae
tr|H6SL22|H6SL22_RHOPH	1	Proteobacteria	Alphaproteobacteria	Rhodospirillum photometricum
tr|B1XY79|B1XY79_LEPCP	1	Proteobacteria	Betaproteobacteria	Leptothrix cholodnii
tr|Q0BS50|Q0BS50_GRABC	1	Proteobacteria	Alphaproteobacteria	Granulibacter bethesdensis

tr|F3S4X7|F3S4X7_9PROT	1	Proteobacteria	Alphaproteobacteria	Gluconacetobacter sp. SXCC-1
tr|Q1MPH8|Q1MPH8_LAWIP	1	Proteobacteria	Deltaproteobacteria	Lawsonia intracellularis
tr|M1PHI9|M1PHI9_DESSD	1	Proteobacteria	Deltaproteobacteria	Desulfocapsa sulfexigens
tr|D5EIN1|D5EIN1_CORAD	1	Verrucomicrobia	Opitutae	Coraliomargarita akajimensis
tr|Q2IEA1|Q2IEA1_ANADE	1	Proteobacteria	Deltaproteobacteria	Anaeromyxobacter dehalogenans
tr|S9S5Z1|S9S5Z1_PHAFV	1	Proteobacteria	Alphaproteobacteria	Phaeospirillum fulvum
tr|R5RCH3|R5RCH3_9PROT	1	Proteobacteria		Proteobacteria bacterium CAG:495
tr|A0A098GGM2|A0A098GGM2_TATMI	1	Proteobacteria	Gammaproteobacteria	Tatlockia micdadei
tr|K2IJ94|K2IJ94_9PROT	1	Proteobacteria	Alphaproteobacteria	Oceanibaculum indicum
tr|B5ZDK2|B5ZDK2_GLUDA	1	Proteobacteria	Alphaproteobacteria	Gluconacetobacter diazotrophicus
tr|Q5ZZ42|Q5ZZ42_LEGPH	1	Proteobacteria	Gammaproteobacteria	Legionella pneumophila
tr|I2DY03|I2DY03_9BURK	6	Proteobacteria	Betaproteobacteria	Burkholderia sp. KJ006
tr|R5XVI9|R5XVI9_9PROT	1	Proteobacteria	Alphaproteobacteria	Acetobacter sp. CAG:267
tr|A6DTV1|A6DTV1_9BACT	1	Lentisphaerae	Lentisphaeria	Lentisphaera araneosa
tr|J6I926|J6I926_9FIRM	1	Firmicutes	Negativicutes	Selenomonas sp. CM52
tr|C9KMC3|C9KMC3_9FIRM	1	Firmicutes	Negativicutes	Mitsuokella multacida
tr|A0A0B2JWN9|A0A0B2JWN9_9FIRM	1	Firmicutes	Negativicutes	Anaerovibrio lipolyticus
tr|R6IC34|R6IC34_9FIRM	1	Firmicutes	Negativicutes	Phascolarctobacterium sp. CAG:266
tr|G4Q3J3|G4Q3J3_ACIIR	1	Firmicutes	Negativicutes	Acidaminococcus intestini
tr|M1E695|M1E695_9FIRM	1	Firmicutes	Clostridia	Thermodesulfobium narugense
tr|A0A096AJ86|A0A096AJ86_9FIRM	1	Firmicutes	Negativicutes	Veillonella montpellierensis
tr|R7CT29|R7CT29_9FIRM	1	Firmicutes	Negativicutes	Dialister sp. CAG:357
tr|H5SN55|H5SN55_9ZZZZ	1			uncultured prokaryote
tr|B2KBY4|B2KBY4_ELUMP	1	Elusimicrobia	Elusimicrobia	Elusimicrobium minutum
tr|D9PKF2|D9PKF2_9ZZZZ	1			sediment metagenome
tr|D3L2J2|D3L2J2_9BACT	1	Synergistetes	Synergistia	Anaerobaculum hydrogeniformans
tr|S0EU26|S0EU26_CHTCT	1	Armatimonadetes	Chthonomonadetes	Chthonomonas calidirosea

tr|K5BC95|K5BC95_9MYCO	1	Actinobacteria	Actinobacteria	Mycobacterium hassiacum
tr|D1A3I9|D1A3I9_THECD	2	Actinobacteria	Actinobacteria	Thermomonospora curvata
tr|D1AD50|D1AD50_THECD	2	Actinobacteria	Actinobacteria	Thermomonospora curvata
tr|D5UP33|D5UP33_TSUPD	1	Actinobacteria	Actinobacteria	Tsukamurella paurometabola
tr|E2SGB2|E2SGB2_9ACTO	1	Actinobacteria	Actinobacteria	Aeromicrobium marinum
tr|W8DX92|W8DX92_9FLOR	1	Rhodophyta	Florideophyceae	Gracilaria salicornia
tr|M5DDI3|M5DDI3_CHOCR	1	Rhodophyta	Florideophyceae	Chondrus crispus
tr|M4ITX4|M4ITX4_CALTB	1	Rhodophyta	Florideophyceae	Calliarthron tuberculosum
tr|M4QH14|M4QH14_PYRYE	1	Rhodophyta	Bangiophyceae	Pyropia yezoensis
tr|A0A0B5W5W5|A0A0B5W5W5_9FLOR	1	Rhodophyta	Florideophyceae	Vertebrata lanosa
tr|M1WZE5|M1WZE5_9NOST	1	Cyanobacteria		Richelia intracellularis
tr|K9TI08|K9TI08_9CYAN	1	Cyanobacteria		Oscillatoria acuminata
tr|E0UEU6|E0UEU6_CYAP2	1	Cyanobacteria		Cyanothece sp. PCC 7822
tr|K9SAM0|K9SAM0_9CYAN	1	Cyanobacteria		Geitlerinema sp. PCC 7407
tr|I4GHH3|I4GHH3_MICAE	1	Cyanobacteria		Microcystis aeruginosa
tr|A0A0C2KX23|A0A0C2KX23_9CYAN	1	Cyanobacteria		Tolypothrix campylonemoides
tr|B7KAF9|B7KAF9_CYAP7	1	Cyanobacteria		Cyanothece sp. PCC 7424
tr|Q85G49|Q85G49_CYAME	1	Rhodophyta	Bangiophyceae	Cyanidioschyzon merolae
tr|E1ZQ59|E1ZQ59_CHLVA	1	Chlorophyta	Trebouxiophyceae	Chlorella variabilis
tr|K9ZMD4|K9ZMD4_ANACC	1	Cyanobacteria		Anabaena cylindrica
tr|A0A0C1WCB9|A0A0C1WCB9_9CYAN	1	Cyanobacteria		Scytonema millei
tr|F5UHF2|F5UHF2_9CYAN	1	Cyanobacteria		Microcoleus vaginatus
tr|D8RA32|D8RA32_SELML	1	Streptophyta		Selaginella moellendorffii
tr|K9UM08|K9UM08_9CHRO	1	Cyanobacteria		Chamaesiphon minutus
tr|Q113C6|Q113C6_TRIEI	1	Cyanobacteria		Trichodesmium erythraeum
tr|L8LYK1|L8LYK1_9CYAN	1	Cyanobacteria		Xenococcus sp. PCC 7305
tr|K9PWE9|K9PWE9_9CYAN	1	Cyanobacteria		Leptolyngbya sp. PCC 7376

tr|A0A098G1D3|A0A098G1D3_9GAMM	1	Proteobacteria	Gammaproteobacteria	Legionella fallonii
tr|D3HS59|D3HS59_LEGLN	1	Proteobacteria	Gammaproteobacteria	Legionella longbeachae
tr|E5XTR2|E5XTR2_9ACTO	1	Actinobacteria	Actinobacteria	Segniliparus rugosus
tr|C9LPP2|C9LPP2_9FIRM	1	Firmicutes	Negativicutes	Dialister invisus
tr|A0A0C1XFX3|A0A0C1XFX3_9CYAN	1	Cyanobacteria		Hassallia byssoidea
tr|H1D371|H1D371_9FIRM	1	Firmicutes	Negativicutes	Dialister succinatiphilus
tr|A8ZRS4|A8ZRS4_DESOH	1	Proteobacteria	Deltaproteobacteria	Desulfococcus oleovorans
tr|A0A077K340|A0A077K340_LIBAS	1	Proteobacteria	Alphaproteobacteria	Candidatus Liberibacter asiaticus
tr|D2B9W2|D2B9W2_STRRD	2	Actinobacteria	Actinobacteria	Streptosporangium roseum
tr|C3XC81|C3XC81_OXAFO	1	Proteobacteria	Betaproteobacteria	Oxalobacter formigenes
tr|D8EW13|D8EW13_9DELT	1	Proteobacteria	Deltaproteobacteria	delta proteobacterium NaphS2
tr|N6ZUM6|N6ZUM6_9RHOO	1	Proteobacteria	Betaproteobacteria	Thauera phenylacetica
tr|H0TS64|H0TS64_9BRAD	1	Proteobacteria	Alphaproteobacteria	Bradyrhizobium sp. STM 3843
tr|A0A0B0ENV4|A0A0B0ENV4_9BACT	1	Planctomycetes	Planctomycetia	Candidatus Scalindua brodae
tr|C7LVW2|C7LVW2_DESBD	1	Proteobacteria	Deltaproteobacteria	Desulfomicrobium baculatum
tr|K9HJ50|K9HJ50_9PROT	1	Proteobacteria	Alphaproteobacteria	Caenispirillum salinarum
tr|A6GAD6|A6GAD6_9DELT	1	Proteobacteria	Deltaproteobacteria	Plesiocystis pacifica
tr|B3QVW9|B3QVW9_CHLT3	1	Chlorobi	Chlorobia	Chloroherpeton thalassium
tr|F8F160|F8F160_TRECH	1	Spirochaetes	Spirochaetia	Treponema caldaria
tr|F5Y8F4|F5Y8F4_TREAZ	1	Spirochaetes	Spirochaetia	Treponema azotonutricium
tr|E0RRH8|E0RRH8_SPITD	1	Spirochaetes	Spirochaetia	Spirochaeta thermophila
tr|E1R695|E1R695_SPISS	1	Spirochaetes	Spirochaetia	Spirochaeta smaragdinae
tr|U1LTX9|U1LTX9_9GAMM	1	Proteobacteria	Gammaproteobacteria	Pseudoalteromonas rubra
tr|F5YJA1|F5YJA1_TREPZ	1	Spirochaetes	Spirochaetia	Treponema primitia
tr|S6D5Y1|S6D5Y1_ACEPA	1	Proteobacteria	Alphaproteobacteria	Acetobacter pasteurianus
tr|A0A060QGU1|A0A060QGU1_9PROT	1	Proteobacteria	Alphaproteobacteria	Asaia platycodi
tr|Q0BS79|Q0BS79_GRABC	1	Proteobacteria	Alphaproteobacteria	Granulibacter bethesdensis

tr|W7DWD6|W7DWD6_9PROT	1	Proteobacteria	Alphaproteobacteria	Commensalibacter sp. MX01
tr|A0A060Q9W3|A0A060Q9W3_9PROT	1	Proteobacteria	Alphaproteobacteria	Saccharibacter sp. AM169
tr|A0A011NGR7|A0A011NGR7_9PROT	1	Proteobacteria	Betaproteobacteria	Candidatus Accumulibacter sp. BA-92
tr|F3GK63|F3GK63_PSESJ	1	Proteobacteria	Gammaproteobacteria	Pseudomonas syringae
tr|D2PUA2|D2PUA2_KRIFD	1	Actinobacteria	Actinobacteria	Kribbella flavida
tr|V8TLZ7|V8TLZ7_9CHLA	1	Chlamydiae	Chlamydiia	Chlamydia pecorum
tr|Q5L5P3|Q5L5P3_CHLAB	1	Chlamydiae	Chlamydiia	Chlamydophila abortus
tr|F8L848|F8L848_SIMNZ	1	Chlamydiae	Chlamydiia	Simkania negevensis
tr|K2ED49|K2ED49_9BACT	1			uncultured bacterium
tr|A0A0C1HIS7|A0A0C1HIS7_9CHLA	1	Chlamydiae	Chlamydiia	Neochlamydia sp. EPS4
tr|A0A0C2YU48|A0A0C2YU48_MAGMG	1	Proteobacteria	Alphaproteobacteria	Magnetospirillum magnetotacticum
tr|Q2RQ88|Q2RQ88_RHORT	1	Proteobacteria	Alphaproteobacteria	Rhodospirillum rubrum
tr|C5JAQ5|C5JAQ5_9BACT	1			uncultured bacterium
tr|W6KBR3|W6KBR3_9PROT	1	Proteobacteria	Alphaproteobacteria	Magnetospira sp. QH-2
tr|A0A0B0HB63|A0A0B0HB63_SOVGS	1	Proteobacteria	Gammaproteobacteria	Solemya velum gill symbiont
tr|D5BR40|D5BR40_PUNMI	1	Proteobacteria	Alphaproteobacteria	Candidatus Puniceispirillum marinum
tr|G5ZYC3|G5ZYC3_9PROT	1	Proteobacteria	Alphaproteobacteria	SAR116 cluster alpha proteobacterium HIMB100
tr|F9UI97|F9UI97_9GAMM	1	Proteobacteria	Gammaproteobacteria	Thiocapsa marina
tr|L0R8C9|L0R8C9_9DELT	1	Proteobacteria	Deltaproteobacteria	Desulfovibrio hydrothermalis
tr|W5YCB9|W5YCB9_KOMXY	1	Proteobacteria	Alphaproteobacteria	Komagataeibacter xylinus
tr|A0A0B8NN74|A0A0B8NN74_9NOCA	1	Actinobacteria	Actinobacteria	Nocardia seriolae
tr|L8DGA8|L8DGA8_9NOCA	1	Actinobacteria	Actinobacteria	Rhodococcus sp. AW25M09
tr|G7GXB0|G7GXB0_9ACTO	1	Actinobacteria	Actinobacteria	Gordonia araii
tr|A0A089UGL1|A0A089UGL1_MYCAB	1	Actinobacteria	Actinobacteria	Mycobacterium abscessus
tr|G8RXX0|G8RXX0_MYCRN	1	Actinobacteria	Actinobacteria	Mycobacterium rhodesiae
tr|D6Z975|D6Z975_SEGRD	1	Actinobacteria	Actinobacteria	Segniliparus rotundus
tr|L7LDL3|L7LDL3_9ACTO	1	Actinobacteria	Actinobacteria	Gordonia hirsuta

tr|H6MY46|H6MY46_GORPV	1	Actinobacteria	Actinobacteria	Gordonia polyisoprenivorans
tr|H0QWP4|H0QWP4_9ACTO	1	Actinobacteria	Actinobacteria	Gordonia effusa
tr|H5UKZ0|H5UKZ0_9ACTO	1	Actinobacteria	Actinobacteria	Gordonia terrae
tr|D5UW68|D5UW68_TSUPD	1	Actinobacteria	Actinobacteria	Tsukamurella paurometabola
tr|B3QS87|B3QS87_CHLT3	1	Chlorobi	Chlorobia	Chloroherpeton thalassium
tr|Q029L2|Q029L2_SOLUE	1	Acidobacteria	Solibacteres	Candidatus Solibacter usitatus
tr|G2LE23|G2LE23_CHLTF	1	Acidobacteria		Candidatus Chloracidobacterium thermophilum
tr|L8D9B1|L8D9B1_9NOCA	1	Actinobacteria	Actinobacteria	Rhodococcus sp. AW25M09
tr|M3VHL4|M3VHL4_9ACTO	1	Actinobacteria	Actinobacteria	Gordonia paraffinivorans
tr|H0R661|H0R661_9ACTO	1	Actinobacteria	Actinobacteria	Gordonia effusa
tr|B1MHM4|B1MHM4_MYCA9	1	Actinobacteria	Actinobacteria	Mycobacterium abscessus
tr|A0A022LUR2|A0A022LUR2_9ACTO	1	Actinobacteria	Actinobacteria	Dietzia sp. UCD-THP
tr|L7L6L0|L7L6L0_9ACTO	1	Actinobacteria	Actinobacteria	Gordonia hirsuta
tr|A0A0A1DIU8|A0A0A1DIU8_NOCSI	1	Actinobacteria	Actinobacteria	Pimelobacter simplex
tr|E9UWP5|E9UWP5_9ACTO	1	Actinobacteria	Actinobacteria	Nocardioidaceae bacterium Broad-1
tr|H5X8F9|H5X8F9_9PSEU	1	Actinobacteria	Actinobacteria	Saccharomonospora marina
tr|I0P733|I0P733_MYCAB	1	Actinobacteria	Actinobacteria	Mycobacterium abscessus
tr|E2S872|E2S872_9ACTO	1	Actinobacteria	Actinobacteria	Aeromicrobium marinum
tr|H0RDR8|H0RDR8_9ACTO	1	Actinobacteria	Actinobacteria	Gordonia polyisoprenivorans
tr|H0R4X6|H0R4X6_9ACTO	1	Actinobacteria	Actinobacteria	Gordonia effusa
tr|A0A0B8NCM8|A0A0B8NCM8_9NOCA	1	Actinobacteria	Actinobacteria	Nocardia seriolae
tr|L7K4P6|L7K4P6_GORRU	1	Actinobacteria	Actinobacteria	Gordonia rubripertincta
tr|D5UYR7|D5UYR7_TSUPD	1	Actinobacteria	Actinobacteria	Tsukamurella paurometabola
tr|H6R8W4|H6R8W4_NOCCG	1	Actinobacteria	Actinobacteria	Nocardia cyriacigeorgica
tr|D5UW67|D5UW67_TSUPD	1	Actinobacteria	Actinobacteria	Tsukamurella paurometabola
tr|U5ELN3|U5ELN3_NOCAS	1	Actinobacteria	Actinobacteria	Nocardia asteroides
tr|L7LEC1|L7LEC1_9ACTO	1	Actinobacteria	Actinobacteria	Gordonia hirsuta

tr|L7LDJ2|L7LDJ2_9ACTO	1	Actinobacteria	Actinobacteria	Gordonia hirsuta
tr|D6Z974|D6Z974_SEGRD	1	Actinobacteria	Actinobacteria	Segniliparus rotundus
tr|V7KTT9|V7KTT9_MYCPC	1	Actinobacteria	Actinobacteria	Mycobacterium avium
tr|F6EN08|F6EN08_AMYSD	1	Actinobacteria	Actinobacteria	Amycolicicoccus subflavus
tr|L7KXK9|L7KXK9_9ACTO	1	Actinobacteria	Actinobacteria	Gordonia amicalis
tr|C6WN65|C6WN65_ACTMD	6	Actinobacteria	Actinobacteria	Actinosynnema mirum
tr|W8HEA5|W8HEA5_RHOOP	2	Actinobacteria	Actinobacteria	Rhodococcus opacus
tr|H0R406|H0R406_9ACTO	1	Actinobacteria	Actinobacteria	Gordonia effusa
tr|A0A081I1V3|A0A081I1V3_9MYCO	1	Actinobacteria	Actinobacteria	Mycobacterium sp. TKK-01-0059
tr|D5P5W1|D5P5W1_9MYCO	1	Actinobacteria	Actinobacteria	Mycobacterium parascrofulaceum
tr|G7H7F9|G7H7F9_9ACTO	1	Actinobacteria	Actinobacteria	Gordonia araii
tr|A0A0B8NIF5|A0A0B8NIF5_9NOCA	1	Actinobacteria	Actinobacteria	Nocardia seriolae
tr|J9S094|J9S094_9ACTO	1	Actinobacteria	Actinobacteria	Gordonia sp. KTR9
tr|K0VAU2|K0VAU2_MYCFO	1	Actinobacteria	Actinobacteria	Mycobacterium fortuitum
tr|F6EHS3|F6EHS3_AMYSD	1	Actinobacteria	Actinobacteria	Amycolicicoccus subflavus
tr|H0ITI6|H0ITI6_MYCAB	1	Actinobacteria	Actinobacteria	Mycobacterium abscessus
tr|H0QWG1|H0QWG1_9ACTO	1	Actinobacteria	Actinobacteria	Gordonia effusa
tr|H5U1P1|H5U1P1_9ACTO	1	Actinobacteria	Actinobacteria	Gordonia sputi
tr|H5UG91|H5UG91_9ACTO	1	Actinobacteria	Actinobacteria	Gordonia terrae
tr|H0RMK6|H0RMK6_9ACTO	1	Actinobacteria	Actinobacteria	Gordonia polyisoprenivorans
tr|B3SFA0|B3SFA0_TRIAD	37	Placozoa		Trichoplax adhaerens
tr|L7L1E9|L7L1E9_9ACTO	1	Actinobacteria	Actinobacteria	Gordonia amicalis
tr|R6PMH5|R6PMH5_9CLOT	1	Firmicutes	Clostridia	Clostridium sp. CAG:306
tr|R5K923|R5K923_9CLOT	1	Firmicutes	Clostridia	Clostridium sp. CAG:967
tr|R5SYZ8|R5SYZ8_9GAMM	1	Proteobacteria	Gammaproteobacteria	Acinetobacter sp. CAG:196
tr|R7MF15|R7MF15_9CLOT	1	Firmicutes	Clostridia	Clostridium sp. CAG:813
tr|K2EV59|K2EV59_9BACT	1			uncultured bacterium

tr|R5H5L0|R5H5L0_9SPIR	1	Spirochaetes	Spirochaetia	Brachyspira sp. CAG:484
tr|F7US12|F7US12_SYNYG	1	Cyanobacteria		Synechocystis sp. PCC 6803
tr|L8LQP9|L8LQP9_9CHRO	1	Cyanobacteria		Gloeocapsa sp. PCC 73106
tr|D8FZ94|D8FZ94_9CYAN	1	Cyanobacteria		[Oscillatoria] sp. PCC 6506
tr|I4INL4|I4INL4_MICAE	1	Cyanobacteria		Microcystis aeruginosa
tr|A0A073CN05|A0A073CN05_PLAAG	1	Cyanobacteria		Planktothrix agardhii
tr|A0A068MXK0|A0A068MXK0_SYNY4	1	Cyanobacteria		Synechocystis sp. PCC 6714
tr|K9YNG8|K9YNG8_CYASC	1	Cyanobacteria		Cyanobacterium stanieri
tr|K9Z8P8|K9Z8P8_CYAAP	1	Cyanobacteria		Cyanobacterium aponinum
tr|V5V315|V5V315_9CHRO	1	Cyanobacteria		Thermosynechococcus sp. NK55a
tr|U5QHE0|U5QHE0_9CYAN	1	Cyanobacteria	Gloeobacteria	Gloeobacter kilaueensis
tr|A0A061RFD7|A0A061RFD7_9CHLO	1	Chlorophyta	Chlorodendrophyceae	Tetraselmis sp. GSL018
tr|M4F3Z1|M4F3Z1_BRARP	23	Streptophyta		Brassica rapa
tr|K8F4W2|K8F4W2_9CHLO	1	Chlorophyta	Mamiellophyceae	Bathycoccus prasinos
tr|R7WDS0|R7WDS0_AEGTA	1	Streptophyta	Liliopsida	Aegilops tauschii
tr|I2PX81|I2PX81_9DELT	1	Proteobacteria	Deltaproteobacteria	Desulfovibrio sp. U5L
tr|U7D4Y0|U7D4Y0_9BACT	1	Fibrobacteres	Chitinivibrionia	Chitinivibrio alkaliphilus
tr|I7A3J2|I7A3J2_MELRP	1	Ignavibacteriae	Ignavibacteria	Melioribacter roseus
tr|B3QRY4|B3QRY4_CHLT3	1	Chlorobi	Chlorobia	Chloroherpeton thalassium
tr|G7VAB7|G7VAB7_THELD	1	Synergistetes	Synergistia	Thermovirga lienii
tr|B1ZUD3|B1ZUD3_OPITP	1	Verrucomicrobia	Opitutae	Opitutus terrae
tr|A0A061R3K3|A0A061R3K3_9CHLO	1	Chlorophyta	Chlorodendrophyceae	Tetraselmis sp. GSL018
tr|A0A0B2S1Y9|A0A0B2S1Y9_GLYSO	1	Streptophyta		Glycine soja
tr|M0VLR3|M0VLR3_HORVD	1	Streptophyta	Liliopsida	Hordeum vulgare
tr|D8TMP4|D8TMP4_VOLCA	1	Chlorophyta	Chlorophyceae	Volvox carteri
tr|I0AJV2|I0AJV2_IGNAJ	1	Ignavibacteriae	Ignavibacteria	Ignavibacterium album
tr|I4BAE9|I4BAE9_TURPD	1	Spirochaetes	Spirochaetia	Turneriella parva

tr|A0A084SZ26|A0A084SZ26_9DELT	1	Proteobacteria	Deltaproteobacteria	Cystobacter violaceus
tr|C0BMZ4|C0BMZ4_9BACT	1	Bacteroidetes	Flavobacteriia	Flavobacteria bacterium MS024-3C
tr|A0A060Q9R5|A0A060Q9R5_9PROT	1	Proteobacteria	Alphaproteobacteria	Saccharibacter sp. AM169
tr|E6Q2N8|E6Q2N8_9ZZZZ	1			mine drainage metagenome
tr|X1IP95|X1IP95_9ZZZZ	1			marine sediment metagenome
tr|B9XRW0|B9XRW0_PEDPL	1	Verrucomicrobia	Verrucomicrobiae	Pedosphaera parvula
tr|R7CU31|R7CU31_9FIRM	1	Firmicutes	Negativicutes	Dialister sp. CAG:357
tr|W1S137|W1S137_9SPHN	1	Proteobacteria	Alphaproteobacteria	Sphingobium sp. C100
tr|A0A0A6CX02|A0A0A6CX02_9SPHN	1	Proteobacteria	Alphaproteobacteria	Sphingomonas sp. Ant20
tr|A5PDE9|A5PDE9_9SPHN	1	Proteobacteria	Alphaproteobacteria	Erythrobacter sp. SD-21
tr|H0E7N6|H0E7N6_9ACTN	1	Actinobacteria	Actinobacteria	Patulibacter medicamentivorans
tr|A6CDE5|A6CDE5_9PLAN	1	Planctomycetes	Planctomycetia	Planctomyces maris
tr|X8F536|X8F536_MYCUL	1	Actinobacteria	Actinobacteria	Mycobacterium ulcerans
tr|A0A024JUZ3|A0A024JUZ3_9MYCO	1	Actinobacteria	Actinobacteria	Mycobacterium triplex
tr|E9USY5|E9USY5_9ACTO	1	Actinobacteria	Actinobacteria	Nocardioidaceae bacterium Broad-1
tr|A0A076MKP2|A0A076MKP2_AMYME	1	Actinobacteria	Actinobacteria	Amycolatopsis methanolica
tr|A0A0B5F9V1|A0A0B5F9V1_9ACTO	1	Actinobacteria	Actinobacteria	Streptomyces albus
tr|A0A076MT28|A0A076MT28_AMYME	1	Actinobacteria	Actinobacteria	Amycolatopsis methanolica
tr|A0A0B5F6P9|A0A0B5F6P9_9ACTO	1	Actinobacteria	Actinobacteria	Streptomyces albus
tr|H0EAN1|H0EAN1_9ACTN	1	Actinobacteria	Actinobacteria	Patulibacter medicamentivorans
tr|E9USY2|E9USY2_9ACTO	1	Actinobacteria	Actinobacteria	Nocardioidaceae bacterium Broad-1
tr|H0E6M7|H0E6M7_9ACTN	1	Actinobacteria	Actinobacteria	Patulibacter medicamentivorans
tr|H0E6M4|H0E6M4_9ACTN	1	Actinobacteria	Actinobacteria	Patulibacter medicamentivorans
tr|A0A0B5EZK5|A0A0B5EZK5_9ACTO	1	Actinobacteria	Actinobacteria	Streptomyces albus
tr|H0E5N7|H0E5N7_9ACTN	1	Actinobacteria	Actinobacteria	Patulibacter medicamentivorans
tr|A0A076N0G6|A0A076N0G6_AMYME	1	Actinobacteria	Actinobacteria	Amycolatopsis methanolica
tr|D3F0X6|D3F0X6_CONWI	1	Actinobacteria	Actinobacteria	Conexibacter woesei

tr|H0E5K5|H0E5K5_9ACTN	1	Actinobacteria	Actinobacteria	Patulibacter medicamentivorans
tr|X8C6V2|X8C6V2_MYCXE	1	Actinobacteria	Actinobacteria	Mycobacterium xenopi
tr|Q317Q4|Q317Q4_DESAG	1	Proteobacteria	Deltaproteobacteria	Desulfovibrio alaskensis
tr|T2G999|T2G999_DESGI	1	Proteobacteria	Deltaproteobacteria	Desulfovibrio gigas
tr|A0A068JLL7|A0A068JLL7_9DELT	1	Proteobacteria	Deltaproteobacteria	Desulfonatronum thiodismutans
tr|B8FKV6|B8FKV6_DESAA	1	Proteobacteria	Deltaproteobacteria	Desulfatibacillum alkenivorans
tr|D5UYS1|D5UYS1_TSUPD	1	Actinobacteria	Actinobacteria	Tsukamurella paurometabola
tr|G2PRP0|G2PRP0_MURRD	1	Bacteroidetes	Flavobacteriia	Muricauda ruestringensis
tr|H1Y660|H1Y660_9SPHI	1	Bacteroidetes	Sphingobacteriia	Mucilaginibacter paludis
tr|G8TD13|G8TD13_NIAKG	1	Bacteroidetes	Sphingobacteriia	Niastella koreensis
tr|A0A0A2N3S4|A0A0A2N3S4_9FLAO	1	Bacteroidetes	Flavobacteriia	Flavobacterium subsaxonicum
tr|K7Z2L4|K7Z2L4_BDEBC	1	Proteobacteria	Deltaproteobacteria	Bdellovibrio bacteriovorus
tr|D0LTW5|D0LTW5_HALO1	1	Proteobacteria	Deltaproteobacteria	Haliangium ochraceum
tr|R6APB1|R6APB1_9FIRM	1	Firmicutes	Negativicutes	Dialister sp. CAG:486
tr|H0E7N7|H0E7N7_9ACTN	1	Actinobacteria	Actinobacteria	Patulibacter medicamentivorans
tr|H0EAN0|H0EAN0_9ACTN	1	Actinobacteria	Actinobacteria	Patulibacter medicamentivorans
tr|H0E6M6|H0E6M6_9ACTN	1	Actinobacteria	Actinobacteria	Patulibacter medicamentivorans
tr|A0A076MU17|A0A076MU17_AMYME	1	Actinobacteria	Actinobacteria	Amycolatopsis methanolica
tr|A0A076N0H2|A0A076N0H2_AMYME	1	Actinobacteria	Actinobacteria	Amycolatopsis methanolica
tr|H0E8D5|H0E8D5_9ACTN	1	Actinobacteria	Actinobacteria	Patulibacter medicamentivorans
tr|H0E5Y4|H0E5Y4_9ACTN	1	Actinobacteria	Actinobacteria	Patulibacter medicamentivorans
tr|H0EAY5|H0EAY5_9ACTN	1	Actinobacteria	Actinobacteria	Patulibacter medicamentivorans
tr|F8AY49|F8AY49_FRADG	1	Actinobacteria	Actinobacteria	Frankia symbiont of Datisca glomerata
tr|A0A076MM50|A0A076MM50_AMYME	1	Actinobacteria	Actinobacteria	Amycolatopsis methanolica
tr|K5BP85|K5BP85_LEPME	1	Spirochaetes	Spirochaetia	Leptospira meyeri
tr|H2CAK7|H2CAK7_9LEPT	1	Spirochaetes	Spirochaetia	Leptonema illini
tr|N1WL36|N1WL36_9LEPT	1	Spirochaetes	Spirochaetia	Leptospira weilii

tr|R8ZSH3|R8ZSH3_9LEPT	1	Spirochaetes	Spirochaetia	Leptospira yanagawae
tr|F6EN12|F6EN12_AMYSD	1	Actinobacteria	Actinobacteria	Amycolicicoccus subflavus
tr|F6EHS6|F6EHS6_AMYSD	2	Actinobacteria	Actinobacteria	Amycolicicoccus subflavus
tr|D1A3I6|D1A3I6_THECD	1	Actinobacteria	Actinobacteria	Thermomonospora curvata
tr|A3ZZF3|A3ZZF3_9PLAN	1	Planctomycetes	Planctomycetia	Blastopirellula marina
tr|K2DZQ2|K2DZQ2_9BACT	1			uncultured bacterium
tr|D5UYR6|D5UYR6_TSUPD	1	Actinobacteria	Actinobacteria	Tsukamurella paurometabola
tr|I9IY71|I9IY71_MYCAB	1	Actinobacteria	Actinobacteria	Mycobacterium abscessus
tr|W5WX97|W5WX97_BDEBC	1	Proteobacteria	Deltaproteobacteria	Bdellovibrio bacteriovorus
tr|H0E5X9|H0E5X9_9ACTN	1	Actinobacteria	Actinobacteria	Patulibacter medicamentivorans
tr|I3IRA7|I3IRA7_9PLAN	1	Planctomycetes	Planctomycetia	planctomycete KSU-1
tr|L0DA47|L0DA47_SINAD	18	Planctomycetes	Planctomycetia	Singulisphaera acidiphila
tr|C0QWQ9|C0QWQ9_BRAHW	1	Spirochaetes	Spirochaetia	Brachyspira hyodysenteriae
tr|F8AY53|F8AY53_FRADG	1	Actinobacteria	Actinobacteria	Frankia symbiont of Datisca glomerata
tr|H0E9N4|H0E9N4_9ACTN	1	Actinobacteria	Actinobacteria	Patulibacter medicamentivorans
tr|H0E613|H0E613_9ACTN	1	Actinobacteria	Actinobacteria	Patulibacter medicamentivorans
tr|H0E056|H0E056_9ACTN	6	Actinobacteria	Actinobacteria	Patulibacter medicamentivorans
tr|F8AY52|F8AY52_FRADG	1	Actinobacteria	Actinobacteria	Frankia symbiont of Datisca glomerata
tr|H0E5Y2|H0E5Y2_9ACTN	1	Actinobacteria	Actinobacteria	Patulibacter medicamentivorans
tr|H0E4W7|H0E4W7_9ACTN	1	Actinobacteria	Actinobacteria	Patulibacter medicamentivorans
tr|H5X813|H5X813_9PSEU	1	Actinobacteria	Actinobacteria	Saccharomonospora marina
tr|J9Z1B9|J9Z1B9_9PROT	1	Proteobacteria	Alphaproteobacteria	alpha proteobacterium HIMB59
tr|H0E7H8|H0E7H8_9ACTN	1	Actinobacteria	Actinobacteria	Patulibacter medicamentivorans
tr|D3F0X7|D3F0X7_CONWI	1	Actinobacteria	Actinobacteria	Conexibacter woesei
tr|A0A076MX42|A0A076MX42_AMYME	1	Actinobacteria	Actinobacteria	Amycolatopsis methanolica
tr|D5SYW1|D5SYW1_PLAL2	1	Planctomycetes	Planctomycetia	Planctomyces limnophilus
tr|A0A068NM91|A0A068NM91_9BACT	1	Armatimonadetes		Fimbriimonas ginsengisoli

tr|B4D2R0|B4D2R0_9BACT	1	Verrucomicrobia	Spartobacteria	Chthoniobacter flavus
tr|C1AEH1|C1AEH1_GEMAT	1	Gemmatimonadetes	Gemmatimonadetes	Gemmatimonas aurantiaca
tr|D6YWR8|D6YWR8_WADCW	1	Chlamydiae	Chlamydiia	Waddlia chondrophila
tr|A0A090D362|A0A090D362_9CHLA	1	Chlamydiae	Chlamydiia	Criblamydia sequanensis
tr|A0A0C1JWL0|A0A0C1JWL0_9CHLA	1	Chlamydiae	Chlamydiia	Candidatus Protochlamydia amoebophila
tr|H0E8D6|H0E8D6_9ACTN	1	Actinobacteria	Actinobacteria	Patulibacter medicamentivorans
tr|H0E615|H0E615_9ACTN	1	Actinobacteria	Actinobacteria	Patulibacter medicamentivorans
tr|A0A076MU15|A0A076MU15_AMYME	2	Actinobacteria	Actinobacteria	Amycolatopsis methanolica
tr|H0E9M2|H0E9M2_9ACTN	1	Actinobacteria	Actinobacteria	Patulibacter medicamentivorans
tr|R6QJ33|R6QJ33_9CLOT	6	Firmicutes	Clostridia	Clostridium sp. CAG:306
tr|D2R1T9|D2R1T9_PIRSD	1	Planctomycetes	Planctomycetia	Pirellula staleyi
tr|D1AD46|D1AD46_THECD	2	Actinobacteria	Actinobacteria	Thermomonospora curvata
tr|A0A0C1DR60|A0A0C1DR60_9NOCA	1	Actinobacteria	Actinobacteria	Nocardia vulneris
tr|H0E5Y3|H0E5Y3_9ACTN	1	Actinobacteria	Actinobacteria	Patulibacter medicamentivorans
tr|H0E6M5|H0E6M5_9ACTN	1	Actinobacteria	Actinobacteria	Patulibacter medicamentivorans
tr|H0EAY6|H0EAY6_9ACTN	1	Actinobacteria	Actinobacteria	Patulibacter medicamentivorans
tr|H0E7H9|H0E7H9_9ACTN	1	Actinobacteria	Actinobacteria	Patulibacter medicamentivorans
tr|M8AXE3|M8AXE3_TRIUA	1	Streptophyta	Liliopsida	Triticum urartu
tr|F2NHU4|F2NHU4_DESAR	1	Proteobacteria	Deltaproteobacteria	Desulfobacca acetoxidans
tr|W7R1V4|W7R1V4_9ALTE	1	Proteobacteria	Gammaproteobacteria	Catenovulum agarivorans
tr|H0E5Y1|H0E5Y1_9ACTN	1	Actinobacteria	Actinobacteria	Patulibacter medicamentivorans
tr|R5H6Y8|R5H6Y8_9SPIR	1	Spirochaetes	Spirochaetia	Brachyspira sp. CAG:484
tr|R5DQ85|R5DQ85_9CLOT	1	Firmicutes	Clostridia	Clostridium sp. CAG:715
tr|B5JTN8|B5JTN8_9GAMM	1	Proteobacteria	Gammaproteobacteria	gamma proteobacterium HTCC5015
tr|X1BXX8|X1BXX8_9ZZZZ	1			marine sediment metagenome
tr|X8C7T7|X8C7T7_MYCXE	1	Actinobacteria	Actinobacteria	Mycobacterium xenopi
tr|J0P0C1|J0P0C1_9SPHI	1	Bacteroidetes	Sphingobacteriia	Saprospira grandis

tr|K0NE48|K0NE48_DESTT	1	Proteobacteria	Deltaproteobacteria	Desulfobacula toluolica
tr|S0FWQ6|S0FWQ6_9DELT	1	Proteobacteria	Deltaproteobacteria	Desulfotignum phosphitoxidans
tr|A8ZZZ7|A8ZZZ7_DESOH	1	Proteobacteria	Deltaproteobacteria	Desulfococcus oleovorans
tr|A0A0C1ZAS7|A0A0C1ZAS7_9DELT	6	Proteobacteria	Deltaproteobacteria	Enhygromyxa salina
tr|L0D9L0|L0D9L0_SINAD	1	Planctomycetes	Planctomycetia	Singulisphaera acidiphila
tr|E8R0Y9|E8R0Y9_ISOPI	1	Planctomycetes	Planctomycetia	Isosphaera pallida
tr|H5SCE0|H5SCE0_9BACT	1	Planctomycetes	Planctomycetia	uncultured planctomycete
tr|R7M8T4|R7M8T4_9CLOT	1	Firmicutes	Clostridia	Clostridium sp. CAG:813
tr|K2DY95|K2DY95_9BACT	6			uncultured bacterium
tr|W0RIJ3|W0RIJ3_9BACT	1	Gemmatimonadetes	Gemmatimonadetes	Gemmatimonadetes bacterium KBS708
tr|L0D8H7|L0D8H7_SINAD	1	Planctomycetes	Planctomycetia	Singulisphaera acidiphila
tr|E8QZ15|E8QZ15_ISOPI	1	Planctomycetes	Planctomycetia	Isosphaera pallida
tr|K2E2I3|K2E2I3_9BACT	1			uncultured bacterium
tr|A0A081C4Z6|A0A081C4Z6_9BACT	1			bacterium UASB270
tr|M5UNM7|M5UNM7_9PLAN	1	Planctomycetes	Planctomycetia	Rhodopirellula sallentina
tr|C9RQK3|C9RQK3_FIBSS	1	Fibrobacteres	Fibrobacteria	Fibrobacter succinogenes
tr|T1CFS9|T1CFS9_9ZZZZ	1			mine drainage metagenome
tr|X8C791|X8C791_MYCXE	1	Actinobacteria	Actinobacteria	Mycobacterium xenopi
tr|W6THL8|W6THL8_HOLOB	1	Proteobacteria	Alphaproteobacteria	Holospora obtusa
